# Fetuin-A/Albumin-Mineral Complexes Resembling Serum Calcium Granules and Putative Nanobacteria: Demonstration of a Dual Inhibition-Seeding Concept

**DOI:** 10.1371/journal.pone.0008058

**Published:** 2009-11-30

**Authors:** Cheng-Yeu Wu, Jan Martel, David Young, John D. Young

**Affiliations:** 1 Laboratory of Nanomaterials, Chang Gung University, Gueishan, Taiwan, Republic of China; 2 Research Center of Bacterial Pathogenesis, Chang Gung University, Gueishan, Taiwan, Republic of China; 3 Department of Biochemistry and Molecular Biology, Graduate Institute of Biomedical Sciences, Chang Gung University, Gueishan, Taiwan, Republic of China; 4 Department of Materials Science and Engineering, Massachusetts Institute of Technology, Cambridge, Massachusetts, United States of America; 5 Laboratory of Cellular Physiology and Immunology, The Rockefeller University, New York, New York, United States of America; 6 Biochemical Engineering Research Center, Mingchi University of Technology, Taipei, Taiwan, Republic of China; University of California Merced, United States of America

## Abstract

Serum-derived granulations and purported nanobacteria (NB) are pleomorphic apatite structures shown to resemble calcium granules widely distributed in nature. They appear to be assembled through a dual inhibitory-seeding mechanism involving proteinaceous factors, as determined by protease (trypsin and chymotrypsin) and heat inactivation studies. When inoculated into cell culture medium, the purified proteins fetuin-A and albumin fail to induce mineralization, but they will readily combine with exogenously added calcium and phosphate, even in submillimolar amounts, to form complexes that will undergo morphological transitions from nanoparticles to spindles, films, and aggregates. As a mineralization inhibitor, fetuin-A is much more potent than albumin, and it will only seed particles at higher mineral-to-protein concentrations. Both proteins display a bell-shaped, dose-dependent relationship, indicative of the same dual inhibitory-seeding mechanism seen with whole serum. As ascertained by both seeding experiments and gel electrophoresis, fetuin-A is not only more dominant but it appears to compete avidly for nanoparticle binding at the expense of albumin. The nanoparticles formed in the presence of fetuin-A are smaller than their albumin counterparts, and they have a greater tendency to display a multi-layered ring morphology. In comparison, the particles seeded by albumin appear mostly incomplete, with single walls. Chemically, spectroscopically, and morphologically, the protein-mineral particles resemble closely serum granules and NB. These particles are thus seen to undergo an amorphous to crystalline transformation, the kinetics and completeness of which depend on the protein-to-mineral ratios, with low ratios favoring faster conversion to crystals. Our results point to a dual inhibitory-seeding, de-repression model for the assembly of particles in supersaturated solutions like serum. The presence of proteins and other inhibitory factors tend to block apatite nuclei formation or to stabilize the nascent nuclei as amorphous or semi-crystalline spherical nanoparticles, until the same inhibitory influences are overwhelmed or de-repressed, whereby the apatite nuclei grow in size to coalesce into crystalline spindles and films—a mechanism that may explain not only the formation of calcium granules in nature but also normal or ectopic calcification in the body.

## Introduction

The experiments reported here represent the continuation of our studies on the mechanisms by which nanoparticles resembling the so-called nanobacteria (NB) are formed and by which anomalous ectopic calcification is triggered [1]–[3]. NB are supposed to represent exotic, slow growing, pleomorphic, sub-micrometer (50–500 nm) microorganisms coated with proteins and containing carbonate hydroxyapatite (HAP) [4]–[12]. These putative microorganisms are deemed identical to the spherical forms observed earlier by Folk in geological samples and which he called ‘nannobacteria’ [4], [13], [14]. The purported NB are ubiquitous, being found not only in soils [13], [14], water [15], [16], and air [17], but also in harsh and remote environments like meteorites [18], stratosphere [19], [20], and even interstellar dust [21]. They are supposedly associated with body fluids [4]–[12], body infusion products and vaccines [4], [22] and have been described as causative or aggravating agents of numerous diseases, especially extraskeletal calcifications ([4]–[12]; see also ref. [23] for a list of such ectopic calcifications with a potential link to NB). NB have thus been implicated in kidney stone formation, calcifications, and polycystic disorders [5], [8], [9], [11], [12], [24]–[27] as well as atherosclerosis and other cardiovascular calcifications [28]–[32]. Notably, NB have been characterized from the outset as infectious agents of disease [5]–[12], [23]. Thus, while NB are now referred also as calcifying nanoparticles (CNP)—a term that more appropriately acknowledges the microbiological and biochemical uncertainties and limitations associated with NB—they continue to be viewed and heralded as infectious and transmissible agents of anomalous ectopic calcifications [6], [7], [10], [26]–[32]. In fact, their infectious nature has been deemed of cardinal importance not only in the understanding of their complex and enigmatic biological cycle [8], [9] but also in providing a basis for justifying the use of antimicrobial therapy regimens to combat supposed NB infections [33]–[35]. As agents of transmissible infectivity, NB are said to fulfill either in part or completely the classic Koch's postulates [6]–[8], [28], [36]–[39]. On these same grounds, NB have been proposed as a potential global health hazard [16], [17], [40], [41]. NB are thus unusual in that they are seen by some [25] not only as primitive symbionts and precursors of biological life but also as infectious agents of disease—extraordinary claims in themselves that have led to intense debate and skepticism. An extensive list of these and other claims made by the NB proponents has been presented earlier [42].

These same views on NB have been refuted on both epistemological [43] and experimental [1]–[3], [44]–[46] grounds. Cisar et al. [44] have provided convincing evidence that challenges the organismic claims made earlier for NB, indicating instead that NB represent lifeless HAP complexed with organic compounds; furthermore, the authors have emphasized the role of lipids like phosphatidylinositol as nucleators of HAP that in turn can reproduce the many morphologies associated with NB. Raoult et al. [46] have reached the conclusion that NB are “mineralo fetuin” complexes that they have renamed as “nanons.” Both these groups have described a protein profile associated with the putative NB grown in serum-free conditions consisting of no more than a few major bands, the main one being fetuin-A, according to Raoult et al. [46]. This simple protein profile stands in marked contrast to the much more complex organismic protein profile that one would have expected for live bacteria and that had in fact been published by other groups [7], [26]. In Cisar et al. 's view [44], the proteins associated with NB grown from the various body fluids are most likely derived from the direct binding of proteins to the growing HAP. Moreover, Raoult et al. [46] have shown that the earlier antibodies marketed as specific for NB, rather than binding to foreign proteins of microorganismic origin, detect fetuin-A instead.

Our own studies have sought to reproduce the NB phenomenology in its entirety by means of a reductionist approach using simple calcium compounds like calcium carbonate as well as other competing ions to reconstitute particles that morphologically resemble NB [1]. Our results are significant in that they demonstrate that various calcium compounds may biomimetically resemble complex biological forms like NB—findings that are consistent with earlier observations made on geological samples enriched for calcite, carbonate, and silicate phases [47]–[50], all of which are capable of biomimetically resembling living organisms. These various studies raise caution against the use of morphological criteria *per se* as the sole basis for implicating biological or life-related activities. In our studies, the initially amorphous calcium particles were seen in the presence of serum to rapidly bind to proteins and ions, to acquire phosphate, to crystallize, and to nucleate further aggregation of HAP [2]. Serum was further shown to inhibit paradoxically the formation of NB-like precipitations, presumably through the binding to calcium and nascent apatite by calcification inhibitors present in the serum—a protective mechanism built-in likely to prevent unwanted calcification [2]. When saturated with calcium phosphate, these same calcification inhibitors—showing high affinities for calcium, nascent apatite, or perhaps both—are thought to turn into seeds for the nucleation of HAP, that upon further phase conversion and growth, end up mimicking and displaying the many peculiar characteristics of NB, including most notably their marked pleomorphism [2]. In our hands, the proteins associated with the NB scaffold were found to include albumin, complement components 3 and 4A, fetuin-A, and apolipoproteins A1 and B100 [2], [3]. This protein profile appears to mirror the very composition of the calcium and apatite binding proteins found in the serum that in turn had been used to culture and assemble NB; their relative abundance found in the NB scaffold appears to vary directly with their respective protein concentrations seen in the feeder serum as well as with their calcium and apatite binding affinities [2]. Accordingly, in the case of NB formed in the presence of other body fluids (saliva, urine, ascites, cerebrospinal fluid, pleural effusion, and synovial fluid), the bound proteins have also turned out to consist of the most abundant calcium-binding proteins found in these fluids, including various immunoglobulin G chains for saliva, and albumin and apolipoprotein A1 for the other body fluids [2]. The NB protein composition can be further modulated by changing or removing the source of proteins comprising the milieu of the nucleating NB, indicating that their presence in the NB scaffold may be circumstantially determined by their availability in the surrounding microenvironment and by their affinity for calcium and apatite [2]. Still in this context, our own exhaustive proteomic analyses of multiple putative NB specimens have revealed only common mammalian proteins that can be entirely explained by the source of serum, body fluid, or tissue used for culturing NB [2], [3], in marked contrast to a more exotic protein profile claimed earlier for NB and comprising of prokaryotic proteins like porins and complex peptidoglycans [5], [24] as well as the bacterial translation elongation factor Tu and the molecular chaperone GroEL [26]. Furthermore, our own immunoblotting experiments have revealed that both polyclonal and monoclonal antibodies, previously claimed to be specific for NB and used in numerous studies to affirm the presence of (and a pathological implication for) NB in human tissues, in fact bind strongly to both albumin and fetuin-A from the *same* as well as *across* species [2]. This surprising finding indicates that all the past immunodetection studies used to implicate a role for NB in human pathologies may very well have detected either albumin or fetuin-A, or a combination of both, derived not only from the very fetal bovine serum (FBS) used for the culture and demonstration of NB, but possibly also from the human tissue under investigation as well. This confusing and misleading scenario now needs to be taken into account when interpreting any past immunodetection study done to ascertain the presence of NB in human tissues.

Our earlier study indicates that the purported NB can be seen as consisting essentially of protein-mineral or organic-mineral complexes formed by means of a dual inhibition-seeding mechanism involving the binding of proteinaceous and other organic calcification inhibitors to calcium and nascent apatite minerals [2]. These inhibitors function presumably within a larger homeostatic cycle of calcium sequestration and clearance [2]. Subsequent experiments have demonstrated the formation of NB-resembling calcium granulations (also referred as calcium granules in the present study) within both fetal bovine and human sera that had been overloaded with either calcium, phosphate, or a combination of both; this granular formation appears to be the result of direct chemical binding between ionic and organic moieties and it precludes the need for any prolonged incubation under culture conditions [3] that earlier had been deemed necessary for the demonstration of NB [4], [5]. By means of simple binding between serum components and calcium and phosphate, we are now able to demonstrate a plethora of NB-like morphologies that include round, laminated particles, spindles, and even films [3]. This same sequestration of calcium in the form of calcium granules is known to occur throughout nature as part of a general cycle of calcium storage, retrieval, deposition, and disposal (see ref. [3] for a more detailed discussion; see Ryall's comprehensive and insightful review [51] on calcium granules, now known to be found in organisms spanning a wide range of phylogenetic complexities).

Against these developments, the NB phenomenology may be seen to have come full circle, with the NB-like precipitations representing no more than the simple outcome of a physiologically relevant homeostatic cycle designed by nature to deal with excess calcium and apatite. As such, and perhaps even ironically, given the controversies generated by this topic, NB-like formations are no less real and they do represent structures that are verifiable and demonstrable. However, they certainly do not seem to represent self-sustained lifeforms, primitive symbionts, or even transmissible and infectious disease agents as originally proposed. Precisely because such particles can be viewed now as comprising a part of the normal cycle of calcium movement and disposal, it is important to understand what exactly determines their assembly, where they are distributed, and whether they have any pathological role after all.

Here, we seek to dissect the role of serum proteins in the assembly of NB-like formations using the *in vitro* reconstitution methods established through our earlier studies [1]–[3]. In our mind, it is critical to establish whether purified proteins like fetuin-A or albumin are capable of replicating the entire NB phenomenology in the absence of other organic moieties (lipids, carbohydrates, and other proteins) that may also be present in the same serum, body fluids, and tissues that had earlier been used to “grow” NB. If so, we want to know whether the simple combination of minerals and proteins can give rise to both the morphological diversity (pleomorphism) and the distinct chemical signature deemed representative of NB-like formations as well as serum calcium granulations [1]–[3].

Both albumin and fetuin-A have been linked to NB-like formations [1]–[3], [46]. We had established earlier that both proteins are among the more abundant proteins found in NB-like particles cultured from FBS or human serum (HS) [1]–[3]. This observation not only reflects the high concentration of albumin and fetuin-A in the serum, but also the fact that these proteins bind avidly to the mineral phase of NB-like particles (see refs. [1]–[3] and the references therein). In our hands, preliminary experiments done with purified proteins indicate that albumin and, even more noticeably, fetuin-A inhibit apatite formation in the presence of excess calcium phosphate (e.g. at supersaturating concentrations) but this inhibition is only transient and it is followed by subsequent mineral precipitation [2]. However, it is not clear whether fetuin-A and albumin will seed apatite when added to a metastable solution exemplified by fresh culture medium, as we seek to verify here. Given the wealth of information accumulated on both albumin and fetuin-A insofar as their roles in calcium and apatite binding and calcification are concerned and their predominance among the proteins species linked to NB-like formations, we want to further establish whether the workings of these proteins alone can explain the entire NB biology, and if so, whether there are differences seen in the presence of either metastable or supersaturated calcium phosphate solutions. Since protein-mineral reactions of this kind may be involved in similar biomineralization processes in the body, we believe that a better understanding of this type of nanoparticle assembly may ultimately help address their pathophysiological role *in vivo*.

## Results and Discussion

### Slow and Spontaneous Formation of NB-Like Precipitations by Serum through a Dual Seeding-Inhibitory Mechanism

All previous NB studies have used a source of proteins like serum, followed by prolonged incubation in cell culture conditions in order to demonstrate the formation of NB-like particles [4]–[9]. This kind of slow culturing process gives at best marginal particle production over a period of several weeks, making the results difficult to quantify or interpret. To address these concerns, we have developed a simple culture methodology that allows for the quantitative and reproducible reconstitution of nanoparticles as well as the simultaneous comparison of a large number of samples [2], [3].

**Figure 1:**
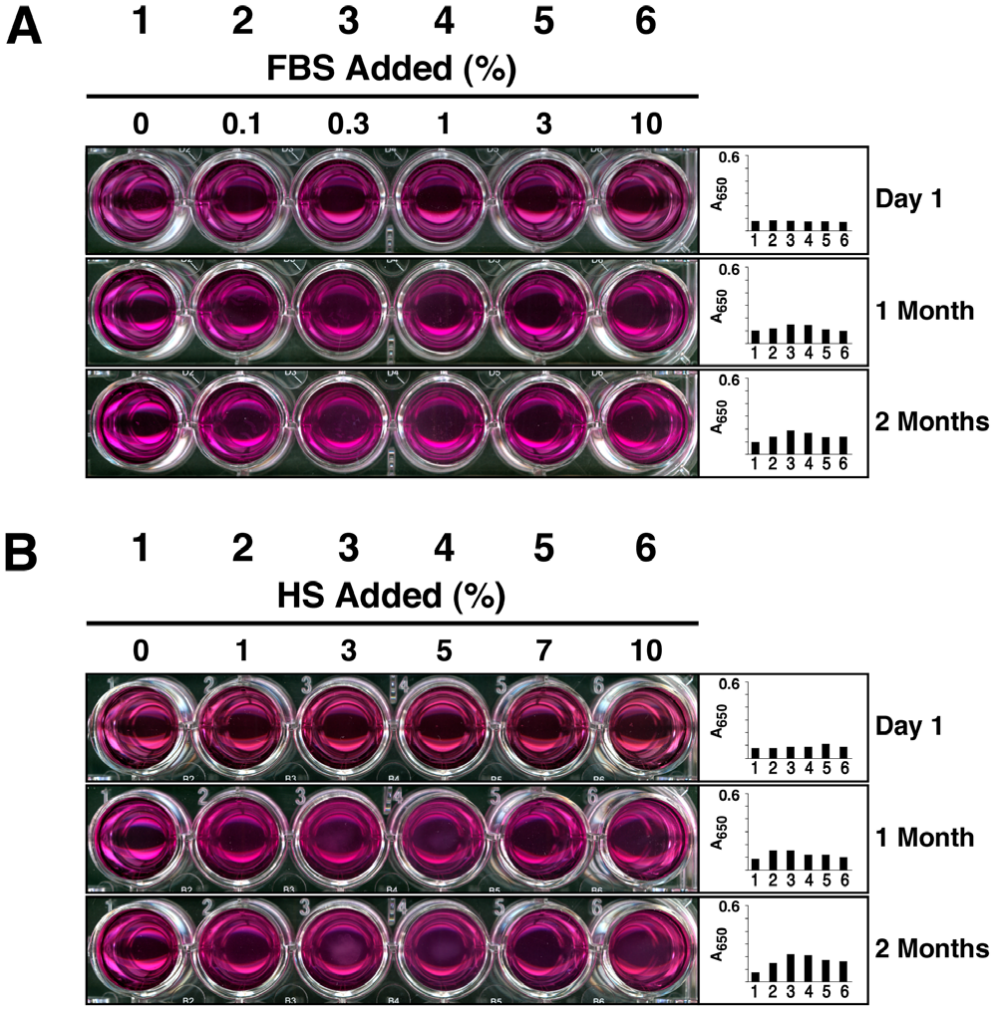
Slow formation of NB-like particles by serum inoculated into DMEM. (A) Formation of NB-like particles by FBS. FBS was inoculated into DMEM to the indicated % by volume. Visual inspection and A_650_ turbidity reading (insets) were performed within 1 hour following inoculation (top panel, “Day 1”) as well as after incubation at 37°C in cell culture conditions for “1 Month” (middle panel) or “2 Months” (bottom panel). Notice the gradual increase in turbidity observed with time as well as the bell-shaped increase in turbidity as a function of the amount of serum inoculated, with maximum turbidity reached at 0.3% FBS, while higher concentrations of FBS resulted in increasingly lower turbidities. (B) Formation of NB-like particles by HS, performed as described in (A), except that higher serum concentrations varying from 1 to 10% were used. Notice the bell-shaped increase in turbidity, with peak reached at 1-to-3% HS at “1 Month” and 3% at “2 Months.”

Figure 1 illustrates the phenomenon of NB-like particle formation using this culture system and in response to the simple inoculation of serum as a source of proteins and other organic compounds. Here, the formation of precipitable complexes resembling NB was monitored in 24-well plates containing Dulbecco's modified Eagle's medium (DMEM) by means of turbidity measurements as captured visually through photography or by means of light spectroscopy reading at a wavelength of 650 nm (A_650_). Turbidity was seen to gradually increase with the addition of either FBS or HS followed by incubation at 37°C for several weeks (Fig. 1). Notably, two types of dose-dependence could be discerned depending on the concentration of serum used. Addition of low concentrations of either FBS or HS produced a dose-dependent increase in turbidity while levels of serum exceeding 0.3% for FBS and 5% for HS generally gave an increasingly lower amount of precipitation or turbidity measurement (Fig. 1A and B, respectively).

This dual dose-dependent response could be illustrated further by fine-tuning the range of serum used. For FBS, we have found that the use of a 100-fold difference in the amount of serum inoculated, varying between 0.1 and 10%, generally encompassed the entire range of dose-dependence observed (Fig. 1A). That is, precipitation could be seen to increase steadily in proportion to the amount of serum added, but only up to 0.3%, as illustrated both by Figure 1A (well 3) shown here as well as by Figure 1A (well 2) shown earlier [2]. Above 0.3% FBS, turbidity decreased proportionally with the amount of serum inoculated, presumably due to calcification-inhibitory influences exerted by the serum (Fig. 1A, wells 4–6). While the peak precipitation seen with 0.3% FBS varied with the source and batch of serum used, a bell-shaped relationship was observed however with all of the FBS samples tested.

For HS, on the other hand, we have noticed generally a rightward shift of this same dose-dependent response when compared to FBS that nonetheless retained its modal or dual nature (Fig. 1B, compare also with Fig. 1A of ref. [2]). A 10-fold HS concentration range was used in the experiment illustrated in Figure 1B to illustrate this characteristic dual dose-dependence and to better highlight the decrease of turbidity associated with higher amounts of serum. Here, peak turbidity in DMEM was seen at 1-to-3% serum after 1 month, and 3% at the end of 2 months (Fig. 1B, middle and bottom rows). This slight rightward shift of the serum concentration associated with culture aging was consistently more noticeable with HS than with FBS. As seen earlier with FBS, while the different HS samples tested gave variable precipitation amounts as well as peak turbidities, the dual or bell-shaped dose-dependence was generally observed with all of the serum samples tested to date.

The bell-shaped serum-dependence for turbidity was interpreted earlier as supporting a dual seeding-inhibitory concept for the formation of NB in the presence of serum, in which calcification inhibitors are seen both as interceptors of calcification as well as seeds for the formation of NB-like calcium granules—a dual role that is modulated by the amounts of calcium and phosphate present in the medium [2], [3]. That is, according to this model, the same calcification inhibitors become calcification-seeding agents when overcome by excess calcium and phosphate. Still in line with this interpretation and in the context of our experiments, low amounts of serum tended to seed NB-like precipitations (due to calcium and phosphate exceeding the inhibitory activity of serum) while higher serum amounts (exceeding 0.3% for FBS and 3% for HS, as determined from the batches of serum tested here) became increasingly inhibitory. This same inhibition appeared to last for several weeks and was evident even after 2 months (Fig. 1A and B, bottom rows). With this prolonged incubation, however, there were signs of partial reversal of the inhibitory activity associated with the serum, which was generally more evident in the case of HS after 2 months (Fig. 1B, note the slight rightward shift for the peak turbidity as compared to the peak seen at 1 month; several other experiments, not shown, have given even more prominent rightward shifts of the peak turbidity as a result of prolonged incubation). That is, although higher amounts of HS still exerted inhibitory influences on the observed precipitation, this same inhibitory effect became much less apparent after an incubation of 2 months (Fig. 1B, compare the middle and bottom rows, wells 5 and 6). For FBS, on the other hand, the inhibitory patterns tended to always last longer, and in this particular experiment, inhibition could still be clearly seen after 2 months with FBS at 1% or higher concentrations (Fig. 1A, bottom row, wells 4–6). This consistent difference between FBS and HS supports the notion that the same calcification-inhibitory influences exerted by HS are probably more easily overcome with incubation than those associated with FBS. This same difference can be further explained by the significantly larger amounts of fetuin-A—one of the more potent calcification inhibitors—found in FBS as compared with HS (10–21 mg/ml in FBS vs. 0.7–0.8 mg/ml in HS; see more detailed discussion in ref. [2] along with the many reference citations therein).

It is further apparent from Figure 1 and from experiments reported elsewhere [2], [3] that the formation of NB-like precipitations is a slow process that takes at least several weeks to develop. The results seen here with both FBS and HS resulting in marginal and barely visible precipitations are in fact representative of hundreds of seeding experiments that we have conducted over a period of two years using a variety of serum batches and different culture conditions. This slow deposition of mineral complexes may explain the earlier lack of reproducible and quantifiable data associated with NB that may have in turn given rise to many erroneous interpretations in the past. Besides, we have noticed a progressive dehydration of the medium used during the course of the prolonged incubation needed for the demonstration of NB, a process that may take upwards to 2 months, and this gradual change in fluid conditions by itself can be shown to lead to nanoparticle precipitation if not properly controlled (data not shown). Nonetheless, at least in our hands, the gradual deposition of mineral complexes in DMEM in response to the presence of serum can indeed be verified as well as quantified.

### Role of Proteins in the Formation of NB-Like Calcium Particles: Effect of Proteases

Earlier results have shown that the inhibition of NB formation produced by high amounts of serum can be abolished with the protease trypsin [2]. Both FBS and HS that had been pre-treated with trypsin readily seeded NB-like formations in a dose-dependent fashion in the presence of exogenously added calcium and phosphate (see Fig. 10 in ref. [2]). That is, upon addition of 1 mM each of calcium and phosphate to DMEM to reach supersaturating levels, there was readily mineral precipitation; this same precipitation could be inhibited by serum in a dose-dependent manner, with inhibition correlating with the amount of serum present [2]. However, serum that had been pre-treated with 0.5% trypsin no longer remained inhibitory [2]. In fact, not only was it no longer inhibitory, this same trypsin-digested serum actually promoted additional seeding of NB-like precipitations in a dose-dependent manner [2]. Seeding seen with trypsinated serum under these conditions was more immediate, taking a few days, rather than several weeks, to complete [2]. These earlier findings support the accepted notion that calcification in the serum—itself considered a supersaturated solution with respect to calcium and phosphate—as well as in living tissues in general is in fact repressed by multiple inhibitory factors [52]–[58]. Our own experiments have indicated that these same inhibitory influences appeared to be at least partially proteinaceous in nature since they were sensitive to tryptic digestion [2]. A subsequent study showed further that treatment with either trypsin or chymotrypsin was capable of precipitating mineral complexes directly from the serum [3]; these same complexes in turn seeded NB-like precipitations upon transfer to fresh medium (see Fig. 12 in ref. [3]). These results demonstrate again the propensity for calcification to occur within serum, and that this propensity is inhibited by serum factors that are themselves sensitive, at least in part, to protease digestion [3]. Our results demonstrate further that the removal or de-repression of these same inhibitory influences appears to be sufficient to bring about mineral precipitation in the serum!

**Figure 2:**
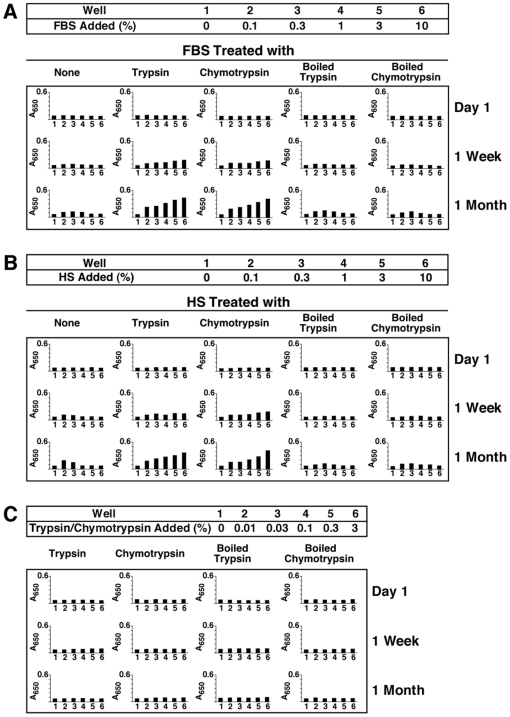
Formation of NB-like particles from protease-treated serum. (A) NB-like particles cultured from protease-treated FBS. Stock solutions of 5% (w/v) trypsin or chymotrypsin were diluted into FBS at the final concentration of 0.5% (v/v), followed by incubation at 37°C for 2 hours. After incubation, the two protease-treated FBS solutions were inoculated into DMEM (columns labeled as “Trypsin” or “Chymotrypsin”) to the concentrations indicated on the top heading, starting from 0% (e.g. untreated, well 1) up to 10% (well 6). FBS was also treated with trypsin or chymotrypsin that had been inactivated by heat at 95°C for 1 hour (“Boiled Trypsin” or “Boiled Chymotrypsin”). For each treatment, A_650_ turbidity readings were performed within 1 hour following inoculation (“Day 1”) and after incubation in cell culture conditions for “1 Week” or “1 Month”, as indicated on the right. Untreated FBS (“None” column) or FBS that was treated with either boiled trypsin or boiled chymotrypsin produced a low bell-shaped curve of turbidity increase after 1 month of incubation. In contrast, inoculation of FBS that was treated with trypsin or chymotrypsin resulted in a more prominent straight dose-dependent turbidity increase after 1 month of incubation. (B) NB-like particles cultured from protease-treated HS were obtained as in (A). Notice the straight dose-dependent increase in turbidity, noticeable at “1 Month,” while both untreated HS and pre-boiled HS produced a low bell-shaped turbidity response. (C) Control experiment, performed with both untreated and boiled proteases inoculated directly into DMEM at the indicated % (w/v). No increase in turbidity was noticed for these control treatments following incubation.

Given the importance of these preliminary results insofar as implicating a role for proteins in the assembly of NB-like particles, we repeated more carefully the trypsin digestion experiment in order to explore in greater detail the nature of the seeding-inhibition relationships associated with serum. This time, following tryptic digestion of both FBS and HS, inoculation of trypsinated serum into DMEM was done without any exogenous addition of calcium or phosphate. Although mineral precipitation seen under these conditions, e.g. in the absence of exogenously added precipitating ions, takes much longer to develop, it allows for the comparison between metastable and supersaturated medium conditions, a distinction which we deem critical in order to assign a definitive role for proteins in nanoparticle formation. Figure 2 shows typical results obtained with both FBS and HS that had been pre-treated with 0.5% trypsin for 2 hours at 37°C, after which the serum was inoculated into DMEM and further incubated for the indicated times. Under these conditions, there was a slow and gradual increase in turbidity associated with the trypsinated serum. This increase in turbidity was significantly higher than that seen with control serum in the same time period (compare 1-month readings of Fig. 2 versus that of Fig. 1; for brevity and ease of comparison, only A_650_ turbidity readings are shown in Fig. 2). Turbidity increases were seen with both trypsin-treated FBS and HS that were more prominent than those seen with control FBS and HS (Fig. 2A and B; compare “None” column with “Trypsin” column). DMEM inoculated with either trypsinated FBS or trypsinated HS failed to display a bell-shaped, dose-dependent precipitation (Fig. 2A and B; note the bell-shaped increase in turbidity evident with 0.1–0.3% of control, untreated FBS and HS that is absent in trypsinated serum). Instead, with both trypsinated FBS and HS, there was a serum dose-dependent increase in turbidity that gradually increased with time.

Similar results were obtained with the protease chymotrypsin tested exactly as trypsin (Fig. 2A and B). Accordingly, both FBS and HS that had been treated with 0.5% chymotrypsin for 2 hours at 37°C proceeded to seed NB-like precipitates slowly in DMEM in a progressive, dose-dependent manner without the bell-shaped curve seen with control, untreated serum (compare “Chymotrypsin” and “Trypsin” columns and these with untreated “None” column in Fig. 2A and B). In this respect, it should be noted that earlier experiments done with both FBS and HS treated with chymotrypsin also gave precipitations that were comparable to those obtained with trypsin [3]. Together, these results indicate that calcification-inhibitory factors in the serum were at least partially removed or neutralized by the proteases trypsin and chymotrypsin. Likewise, it can be concluded that these same inhibitory influences are, at least partially, proteinaceous in nature.

For control, DMEM was inoculated with either FBS or HS pre-treated with trypsin or chymotrypsin that had been heat-inactivated for 1 hour at 95°C (labeled as “Boiled Trypsin” or “Boiled Chymotrypsin” in Fig. 2A and B). We reasoned that this treatment provided a control not only for the presence of trypsin but also for the presence of normal serum in DMEM. As expected, inoculation of DMEM with either FBS or HS that had been pre-treated with boiled trypsin or boiled chymotrypsin produced much lower turbidity readings as compared with that obtained with either trypsinated FBS or trypsinated HS. In fact, the low turbidities obtained under these control conditions were comparable to those seen using untreated serum as depicted earlier in Figure 1 and in Figure 2A and B, seen under the “None” column. That is, in spite of the low turbidities obtained under these control conditions with boiled trypsin or boiled chymotrypsin, a dual dose-dependent response could still be discerned, exactly as seen earlier with untreated serum (Fig. 1 and Fig. 2A and B, “None” column). As additional controls, both untreated and heat-treated trypsin or chymotrypsin were added directly to DMEM to a final concentration of 0.01–3% and the inoculated wells were incubated for one month. No increase in turbidity was observed here as compared with the control background DMEM (Fig. 2C). Given the fact that serum samples pre-treated with 0.5% protease (trypsin or chymotrypsin) were subsequently added to DMEM to 0.1–10%, the protease concentrations used for these last control experiments would represent up to 60 times the amount of trypsin or chymotrypsin used under the study conditions described here. These results further indicate that the seeding effect seen in DMEM is closely associated with body fluids like serum and that this treatment cannot be replaced by the presence of proteases alone. Together, these control experiments demonstrate that the seeding effects seen with either trypsinated or chymotrypsinated serum are not the result of artifacts associated solely with the presence of proteases.

These findings clearly complement and support the earlier results obtained with trypsinated serum inoculated into DMEM that had been supplemented with additional calcium and phosphate [2]. There, adding exogenous calcium and phosphate to DMEM to supersaturating levels resulted in immediate precipitation, which in turn could be effectively inhibited by serum. However, serum that had been trypsinated was shown to be no longer inhibitory but, instead, seeded more NB than could be accounted by the addition of calcium and phosphate alone, with the resultant seeding correlating directly with the amount of trypsinated serum added [2]. Likewise, treatment of serum with either trypsin or chymotrypsin readily resulted in mineral precipitation that in turn could be used to further seed NB-like formations in fresh medium [3]. These results were interpreted as a release of inherent calcification-inhibitory pathways that appeared to be protease-sensitive. Here, in contrast, mineral precipitation was allowed to progress on its own in DMEM without the addition of exogenous precipitating ions. Under these conditions, it can still be seen that the same protease-treated serum also produced more precipitation than control serum; moreover, seeding no longer showed a dual response, indicating the dampening or release of serum-mediated inhibition produced by means of protease treatment. These results suggest that protease-sensitive proteins in the serum may account at least in part for both NB-like seeding and inhibition capabilities associated with normal serum, a fact that can be reproduced with solutions containing both metastable and supersaturating calcium concentrations. About the only difference seen here with the two types of calcium environment is that the calcification reaction is enhanced many fold in the presence of excess calcium under supersaturating conditions as compared with metastable calcium environments. On the other hand, the results shown here do not preclude the participation of other protease-insensitive or non-proteinaceous moieties (perhaps small peptides, glycoproteins, lipids, and carbohydrates) in this type of calcification reaction.

### Role of Proteins in the Formation of NB-Like Calcium Particles: Effect of Heat Treatment and Distinction between Metastable and Supersaturated Calcium Solutions

**Figure 3:**
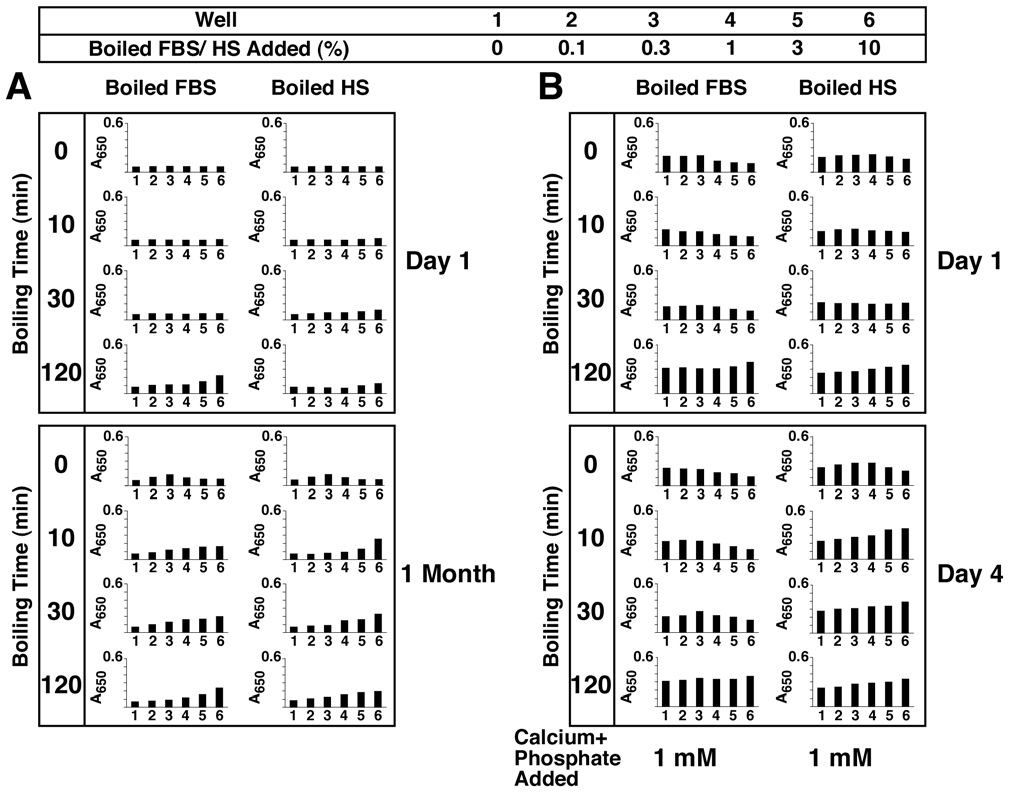
Formation of NB-like particles from boiled serum in metastable versus supersaturated medium. (A) Metastable medium: FBS and 25% HS were boiled at 95°C for the time indicated on the left panel (0, 10, 30, and 120 min). “0 min” refers to control, untreated serum. The boiled sera were then inoculated into DMEM to the concentrations shown on the top heading. (B) Supersaturated medium: both FBS and HS were treated as described in (A), except that 1 mM each of CaCl_2_ and NaH_2_PO_4_ (labeled as “Calcium+Phosphate Added”) was added following the inoculation of the boiled serum into DMEM. Note the marked differences in turbidity changes between the various panels shown in (A) versus (B). See the text for explanation and interpretation.

Other treatments were sought to verify the role of proteinaceous serum factors in creating (or inhibiting) NB-like particles in metastable solutions like DMEM. Heat treatment of proteins is commonly used as a way of denaturing proteins or inducing conformational changes. Figure 3 shows the results obtained with boiling FBS or HS followed by inoculation into DMEM. Heat treatment of membrane-filtered serum was done at 95°C for various periods of time (10, 30, and 120 min). While the boiled serum became more opalescent and occasionally showed white precipitations, the entire serum solution was used for inoculation without further processing. As can be seen, there is an increase in seeding by at least several fold as compared with non-boiled control serum (Fig. 3A). This increase in turbidity seen with serum that had been boiled for 10 or 30 minutes occurred gradually and was not the result of an immediate precipitation due to heat treatment of serum alone (note the low turbidity seen on “Day 1”). However, with longer boiling times (120 min), some immediate precipitations were seen on “Day 1,” which in this particular experiment were more noticeable with FBS as compared to the HS sample used (Fig. 2A). With even longer boiling times (overnight), mineral precipitation was obvious in the serum even prior to seeding (data not shown). Nonetheless, from Figure 3A, it can be seen that there was a slow and gradual increase in turbidity associated with the higher amounts of boiled serum used over the course of several weeks of incubation (compare “1 Month” and “Day 1” readings). However, as evidenced with protease-treated serum, the same bell-shaped, dose-dependent precipitation seen earlier with control, untreated serum was no longer apparent with serum that had been boiled. That is, it appears that the inhibition seen with higher amounts of serum (exceeding 1–3% in the case of FBS and HS) was somehow released through the boiling treatment.

To further verify whether there are differences seen between metastable versus supersaturated calcium and phosphate solutions with respect to the effects of boiled serum, we repeated the same seeding experiment with DMEM shown in Figure 3A, supplemented however with 1 mM calcium and phosphate. Mineral precipitation, along with an increase in turbidity, was immediate under these conditions (Fig. 3B, “Day 1”). Untreated, control serum (both FBS and HS) at higher concentrations (1% for FBS and 3% for HS) was capable of exerting immediate, partial inhibition of this same mineral precipitation (Fig. 3B, see the row “0 min Boiling Time” within the “Day 1” panel), an inhibition that could be sustained for several days (Fig. 3B, compare with “Day 4” panel). Thus, this precipitation showed a prominent bell-shaped, dual relationship as a function of the serum amount added, in line with the data published earlier [2].

In the presence of boiled serum, however, the same DMEM challenged with exogenous calcium and phosphate (1 mM) displayed a pattern of response that differed markedly from that seen with DMEM alone (e.g. without the presence of additional, exogenously added calcium and phosphate). As can be seen in Figure 3B, the addition of 1 mM calcium and phosphate to DMEM produced immediate precipitation associated with turbidity increase (wells 1, “Day 1”). Turbidity did not increase significantly thereafter (compare with “Day 4”). As noted before, the presence of control FBS or HS in DMEM resulted in partial inhibition of this same turbidity produced by 1 mM calcium and phosphate (seen from control rows identified by a boiling time of “0” min, Fig. 3B). Moreover, both FBS and HS that had been boiled for 10 and 30 minutes produced the same partial inhibition of turbidity immediately after seeding (“Day 1”). In the case of FBS, this same partial inhibition could still be seen on Day 4 (Fig. 3B, “Boiled FBS” column). For HS, on the other hand, this inhibition was released after 3 days, being replaced by a straight, dose-dependent change in turbidity that increased with the amount of HS present (Fig. 3B, “Boiled HS, Day 4”). Upon using either FBS or HS that had been boiled for 120 minutes, however, this same inhibition was immediately released and a dose-dependent increase in turbidity could be seen both on “Day 1” and “Day 4” (Fig. 3B). In fact, the addition of boiled serum produced an additive precipitation effect on top of the precipitation seen with the exogenous addition of 1 mM of calcium and phosphate alone that was in itself dose-dependent (Fig. 3B, more evident with all the boiled HS samples tested, but also seen with FBS that had been boiled for 120 min). Upon longer incubation (up to 1 month), a progressive increase of turbidity was observed with all serum treatments, resulting in the reversal or overcoming of the seeding-inhibition seen at the earlier time points (data not shown).

With respect to supersaturated calcium and phosphate solutions, these results would seem to indicate that the calcification-inhibitory influences seen associated with serum are only partially sensitive to heat exposure and that extensive boiling and denaturation are required to effect their inactivation. In this same context, our results indicate that, for FBS, the same calcification-inhibitory factors show a higher resistance to heat inactivation as compared with HS. Our findings also reveal that the “seeding” and “inhibitory” tendencies associated with serum can be clearly distinguished by the experimental set-up used here. Thus, in the case of metastable medium (DMEM), the “seeding” aspects of the boiled serum can be more readily revealed. On the other hand, with supersaturated calcium and phosphate solutions displaying high natural propensity to calcify, it is the “inhibitory” aspects of the serum that are more readily evident and that in turn are either partially or completely removed through boiling.

These results further indicate that the use of metastable and supersaturated calcium and phosphate solutions may allow for the elucidation of certain mechanistic aspects of nanoparticle physiology that would not have been possible by making use of only metastable conditions. Earlier, we used exogenous precipitating ions to enhance the biomineralization process many fold—a strategy that was adopted to amplify precipitation and to obtain more quantifiable and reproducible data [2], [3]. Here, we have noticed that in addition to enhancing the calcification process, the addition of small amounts of calcium and phosphate could be used as a tool to study the role of serum and proteins in the context of the dual inhibition-seeding model proposed here for nanoparticle assembly. It should be further noted that DMEM contains 1.8 mM calcium and 0.9 mM phosphate. The addition of up to 1 mM of calcium and phosphate, as we have done in the experiment depicted in Figure 3B, gives final concentrations of 2.8 mM calcium and 1.9 mM phosphate, resulting in an ion product of 5.32×10^−6^ M^2^, still below the cut-off value of 6×10^−6^ M^2^, above which any solution at physiological pH, ionic strength, and body temperature—serum in body conditions for example—has been found to precipitate spontaneously [56]. Yet, the ion product obtained with such low amounts of calcium and phosphate is sufficient to reveal important differences between this ionic environment and metastable solutions like DMEM. In fact, we have noticed that as little as 0.7 mM calcium and phosphate, and at times, as little as 0.3 mM calcium phosphate, inoculated into DMEM will result in gradual mineral precipitation, giving results that are similar to those seen here with 1 mM calcium and phosphate except for a slower kinetics (data not shown). We have noticed further that the level of precipitating ions required to induce mineral deposition was clearly dependent on the age of the medium, its pH, and other environmental factors, such as the frequency with which the plates were examined and how long they were left out at room temperature (unpublished observations). These results indicate that one can easily simulate supersaturated calcium and phosphate conditions with minimal additions of exogenous calcium and phosphate, enabling one to minimize the ionic perturbation of the medium being studied.

### Formation of Nanoparticles by Fetuin-A and Albumin in Metastable Versus Supersaturated Calcium and Phosphate Solutions

With respect to nanoparticle formation and calcification, our experiments clearly indicate that both inhibitory and seeding influences are seen to be associated with serum, and, furthermore, that at least some of these inhibitory influences can be removed through protease and heat treatment of the serum tested. These results would also suggest a critical role for proteins in this same process of mineral precipitation, perhaps by their initiating nanoparticle formation, itself probably representing an early step of the biomineralization process. This possibility prompted us to investigate more carefully the role of purified serum proteins in mediating nanoparticle formation.

For our protein studies, we chose to use albumin and fetuin-A not only because they had been shown to be involved in calcium nanoparticle formation [1]–[3], [46] but also because of extensive studies pointing to an active role for both proteins in regulating calcium and apatite homeostasis in the blood [56], [57], [59]–[68] as well as in mediating calcification reactions elsewhere in the body [69]–[77]. Fetuin-A, for instance, is a potent calcification inhibitor, known to bind strongly to both calcium and nascent apatite crystals. By itself, fetuin-A accounts for about half to one-third of the total serum inhibitory capacity directed against spontaneous apatite precipitation associated with supersaturated solutions of calcium and phosphate [57], [66]. As for albumin, according to Garnett and Dieppe, it “not only accounts for half of the protein concentration in serum, but also contributes half of the inhibitory activity [directed against apatite crystal growth and associated with] the high-molecular-mass fraction” [62]. Intriguingly, albumin and fetuin-A represent two of the most abundant non-collagenous proteins found in the bones. For instance, albumin is found at 0.7 mg/g of bone in bovines [78] whereas fetuin-A is usually found at 0.9 to 1 mg/g of bone in rats [79], bovines [78], and humans [80]. Both proteins are also found associated with protein-mineral complexes that form in the serum under conditions in which ectopic calcification is triggered. Such protein-mineral complexes have been shown for instance to form in the serum of animals treated with vitamin D, warfarin, etidronate, or adenine, all known to modulate or perturb calcium homeostasis and to induce calcification [69]–[72], [76]. Similarly, both albumin and fetuin-A are present in mineral complexes isolated from humans undergoing severe extra-skeletal calcification as seen in cases of calcifying peritonitis [74]. The concept that these proteins act as systemic calcification inhibitors is illustrated by the observation that fetuin-A-deficient mice spontaneously develop ectopic calcification when fed with a diet rich in mineral and vitamin D [67], [68]. On the other hand, evidence suggests that albumin may promote mineral formation in some experimental conditions, an observation that would confer to this protein a dual inhibition-nucleation role on nanoparticle formation. For instance, albumin at low concentrations (under 10 mg/ml) promote the deposition of calcium phosphate on collagen while higher concentrations (above 10 mg/ml) inhibit collagen mineralization [81], [82]. In addition, albumin has also been shown to promote the precipitation of calcium phosphate in metastable solutions when adsorbed onto HAP crystals [83], [84]. It seems important therefore to verify whether albumin and fetuin-A may lead directly to calcium nanoparticle formation under the culture conditions used to study NB and nanobacteria-like particles (NLP) in the past.

Through our earlier studies, we have shown that both bovine and human forms of albumin and fetuin-A are integral components of calcium nanoparticles formed from serum [1]–[3]. In fact, antibodies thought to be directed earlier against human NB [5], [8], [24], [26]–[29], [35], have been shown to cross-react across species against fetuin-A and albumin from both FBS and HS [1]–[3]. From our own antibody analysis, we have inferred earlier that both bovine and human antigens may be present in all the earlier NB studies done to establish the presence of nanobacterial antigens in human tissues; in those earlier studies, NB were supposedly “grown” in medium supplemented with untreated or gamma-irradiated FBS (described in more detail in refs. [2], [3], and the many references cited therein). Thus, for the purpose of demonstrating a role for proteins in the assembly of NB and NLP, we used both the bovine and human forms of albumin and fetuin-A in the current study. For brevity, however, only results pertaining to human serum albumin (HSA) and bovine serum fetuin-A (BSF) are shown in most experiments. These forms are not only more readily available in commercial quantities but they are used here to allow us to confirm that these proteins will bind strongly to nascent calcifying nanoparticles regardless of their species of origin. It should be noted that the use of the other two forms of these proteins (bovine serum albumin and human serum fetuin-A) have given comparable results.

**Figure 4:**
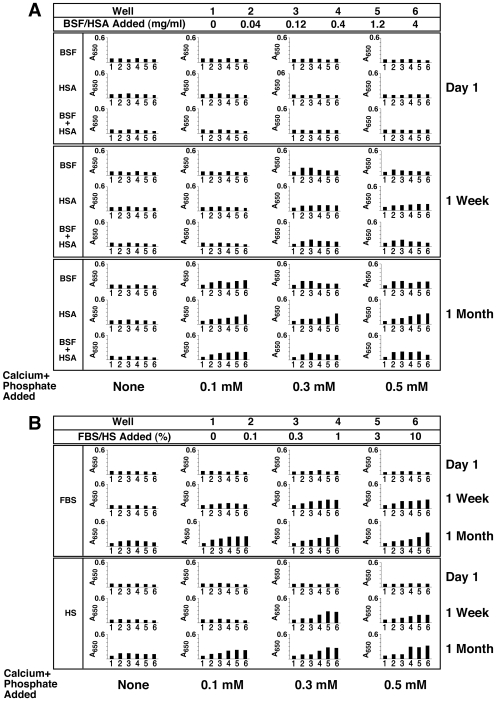
Seeding of NB-like particles by fetuin-A, albumin, and serum in metastable versus supersaturated medium. (A) Fetuin-A and albumin in both metastable and supersaturated media: bovine serum fetuin-A (BSF) and human serum albumin (HSA) were diluted into DMEM, separately or together, to the final concentrations indicated on the top. Where indicated, CaCl_2_ and NaH_2_PO_4_ were added to the final concentrations shown at the bottom (labeled as “Calcium+Phosphate Added”), followed by incubation in cell culture conditions for “1 Week” or “1 Month”, as indicated on the right of each panel. “None” refers to the absence of any exogenously added calcium and phosphate, that is, to metastable medium DMEM. Seeding of the proteins alone without additional ion input did not produce any turbidity change even following incubation for 1 month (“None” column). Low amounts of precipitation were noticed when precipitating ions were added (supersaturated solutions) that increased gradually over time through either bell-shaped (BSF, BSF and HSA) or straight, dose-dependent (HSA) relationships. (B) Serum in supersaturated medium: FBS (top panel) or HS (lower panel) were added to the final concentrations indicated on the top, followed by addition of precipitating reagents to the concentrations shown at the bottom. Addition of precipitating reagents at either 0.1, 0.3, or 0.5 mM into DMEM containing FBS or HS produced turbidities which increased steadily as seen after “1 Week” either through bell-shaped (0.1 mM calcium and phosphate) or straight, dose-dependent (0.3 and 0.5 mM calcium and phosphate) relationships. After further incubation, all treatments showed straight dose-dependent turbidities that continued to increase in a slow and gradual manner (“1 Month”).

Figure 4 (column marked by “None”) shows typical results obtained with inoculating either BSF, HSA, or both into DMEM and observing for turbidity changes over various periods of time (data shown up to 1 month). Surprisingly, no precipitation was seen with any of the three types of inoculation, with proteins added between 0.04 and 4 mg/ml and observed for extended periods of times of up to 2 months (data not shown). These results are representative of a total number of 15 separate experiments done over a course of two years by several independent researchers. Turbidity and precipitation that had been seen in some initial experiments were later traced to bacterial contamination associated with the protein solutions; after filtration through 0.2-µm membranes, proteins no longer produced precipitation in spite of prolonged incubation. To ensure that the results were not due to proteins being filtered out, the same solutions were inoculated without filtration but in the presence of 0.02–0.2% sodium azide added to the medium. No precipitation was seen under these conditions.

**Figure 5 pone-0008058-g005:**
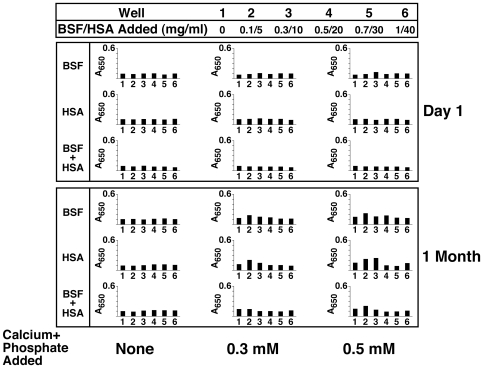
Effect of high concentrations of fetuin-A and albumin on the formation of NB-like particles in metastable versus supersaturated medium. Stock solutions of BSF and/or HSA were diluted into DMEM to the concentrations indicated on the top, so as to mimick the concentration range of these proteins seen in HS. When indicated, the precipitating reagents CaCl_2_ and NaH_2_PO_4_ were added to the concentrations shown at the bottom (labeled as “Calcium+Phosphate Added”). A_650_ turbidity readings were performed following inoculation (“Day 1”) and after incubation in cell culture conditions for “1 Month”, as indicated on the right. Seeding of the proteins alone without additional precipitating reagents did not produce any turbidity increase even following incubation for 1 month (“None” column). Notice the bell-shaped turbidity increases seen with 0.3 mM and 0.5 mM calcium and phosphate columns, observed at “1 Month,” but not “Day 1.”

In other experiments (Fig. 5), albumin was increased 10-fold up to 40 mg/ml so as to mirror its normal concentration in the serum, at 35–45 mg/ml in HS [92] and 23 mg/ml in FBS [93]. Likewise, BSF was tested up to 20 mg/ml in some experiments (not shown). Precipitation was not seen within this broad spectrum of protein concentrations. Precipitation was also not evident when proteins were tested against other cell culture media (Roswell Park Memorial Institute 1640 or RPMI-1640, F12 medium, medium 199, Glascow minimum essential medium, and Leibovitz L-15 medium, all obtained from Gibco; data not shown).

Since neither albumin nor fetuin-A could seed calcium nanoparticles in metastable solutions, inoculation was carried out in DMEM in the presence of small amounts of calcium and phosphate. Figure 4A shows results for three concentrations of calcium and phosphated added: 0.1 mM, 0.3 mM, and 0.5 mM. In the presence of 0.1 mM calcium and phosphate, a progressive, slow, dose-dependent turbidity increase was seen with subsequent addition of either BSF or HSA. In contrast, 0.1 mM calcium and phosphate alone, in the absence of proteins, did not produce any turbidity changes in the same period of observation, indicating that the turbidity seen with either HSA or BSF was not due to spontaneous or homogeneous mineral deposition associated with the presence of precipitating ions. The inoculation of both BSF and HSA in the same wells resulted in a bell-shaped turbidity increase, with peak turbidity induced by 1.2 mg/ml each of BSF and HSA added, and decreasing thereafter with larger doses of protein (4 mg/ml). It was obvious that the inoculation of both proteins together produced an additive inhibitory effect that reflected the summation of the individual effects associated with either BSF or HSA. In all three instances, however, the increase of turbidity was slow, taking at least two weeks to become noticeable.

In the presence of both 0.3 mM and 0.5 mM calcium and phosphate, precipitation could be discerned earlier and, by one week, noticeable increases in turbidity were measured (Fig. 4, “0.3 mM” and “0.5 mM” columns). On the other hand, adding calcium and phosphate alone at these levels produced no increase in turbidity during the same periods of time (up to 1 month, wells 1 in each column, Fig. 4A). In the presence of added calcium and phosphate (0.3 mM and more noticeably with 0.5 mM), a bell-shaped dose-dependence was clearly seen with BSF, but not with HSA. HSA alone produced a simple, dose-dependent increase in turbidity without any noticeable inhibition seen with higher protein concentrations. In the presence of both BSF and HSA, however, the same bell-shaped dose-dependence could also be discerned, seemingly translating into an additive inhibitory effect produced by the individual BSF and HSA moieties. These results indicate that, in addition to both BSF and HSA displaying seeding capability in the presence of small amounts of calcium and phosphate added, only BSF showed clear inhibitory tendencies in the protein concentrations tested here.

To correlate these findings with those seen with unfractionated whole serum, we repeated the same experiments by inoculating FBS or HS into DMEM containing various amounts of calcium and phosphate. Figure 4B demonstrates again the slow, bell-shaped seeding that is seen with either FBS or HS inoculated into DMEM alone in the absence of any externally added calcium and phosphate (“None” column). In the presence of 0.1 mM calcium and phosphate, however, a much more prominent turbidity increase could be seen with either FBS or HS that was not apparent with calcium and phosphate alone. Again, turbidity appeared to peak at 1% FBS and 0.3% HS after 1 week, and at 10% FBS and 3% HS after 1 month (Fig. 4B, “0.1 mM” column). In the presence of higher amounts of calcium and phosphate (0.3 mM and 0.5 mM), the turbidity increase was enhanced, and by one week, precipitation had nearly reached maximal levels (Fig. 4B). It should be noted that, by one week, peak turbidity was seen at 3% FBS or HS, while 10% FBS or HS gave lower turbidities, indicative of a bell-shaped dose-dependence. However, with longer incubations, up to 1 month, some of this same inhibition seen at higher doses of serum appeared to have been subdued or overcome (Fig. 4B). Together, these results indicate a similar type of seeding-inhibition relationship seen with the whole serum that can be mimicked at least in part by the two purified serum proteins albumin and fetuin-A.

That albumin and fetuin-A should differ in their effects on mineral precipitation (Fig. 4A) may be explained by their differential affinities for calcium and apatite. In fact, compared to albumin, fetuin-A is known to bind much more strongly to calcium. For instance, fetuin-A was described to have 6 calcium-binding sites, with one of them being associated with a binding constant of 0.95×10^−4^ M in the case of BSF and 1.42×10^−4^ M for HSF [65]. On the other hand, bovine serum albumin (BSA) also has multiple binding sites, but they are associated with a higher calcium-binding constant of 7×10^−3^ M [65]. Fetuin-A is also known to have unique apatite-binding sites ([94]; reviewed also by Jahnen-Dechent et al. in ref. [75]). In earlier studies on nanoparticle formation, it had also been shown that, compared to albumin, fetuin-A is a much more potent inhibitor of calcification [2]. This inhibition, however, appears to be transient, being overcome gradually through incubation, a phenomenon that seems to correlate with what has been seen with whole serum, as shown here and elsewhere [2].

In addition to the inherent differences in calcium and apatite-binding affinities between fetuin-A and albumin, it is also known that, compared to fetuin-A, albumin is found in much higher amounts in HS. At 35–45 mg/ml in HS [92], albumin is 50–56x more abundant than fetuin-A (0.7–0.8 mg/ml [95]; see also ref. [2]). In FBS, on the other hand and as noted earlier, albumin is reportedly found at 23 mg/ml [93] while fetuin-A has been measured at 10–21 mg/ml [96]. The differences in the concentrations of these two proteins in HS versus FBS as well as the differences in their respective affinities for calcium and apatite indicate that fetuin-A and albumin may exert markedly different seeding or inhibitory influences insofar as nanoparticle formation is concerned.

To address these inherent differences between fetuin-A and albumin, we repeated the same seeding experiments shown in Figure 4A, however this time using higher concentrations of albumin (up to 40 mg/ml). We reasoned that increasing the amounts of albumin used would mirror more closely the normal concentrations of albumin found in both HS and FBS. Figure 5 shows one such series of experiments done in the presence of 0.3 mM and 0.5 mM calcium and phosphate. Either fetuin-A or albumin at the concentrations tested (BSF up to 1 mg/ml; HSA up to 40 mg/ml) produced similar bell-shaped, dose-dependent seedings indicative of seeding-inhibition seen with the higher doses of albumin used. In this particular experiment, BSF and HSA concentrations above 0.1 mg/ml and 5 mg/ml, respectively, gave progressively lower turbidities. When added together, fetuin-A and albumin produced additive inhibitory effects (Fig. 5). Again, in the absence of externally added calcium and phosphate, neither fetuin-A (BSF, 0.1–1 mg/ml) nor albumin (HSA, 5–40 mg/ml) produced any noticeable precipitation after 1 month. Adding the two proteins together (BSF up to 1 mg/ml, HSA up to 40 mg/ml), without any externally added ions, also failed to produce any precipitation (Fig. 5).

**Figure 6 pone-0008058-g006:**
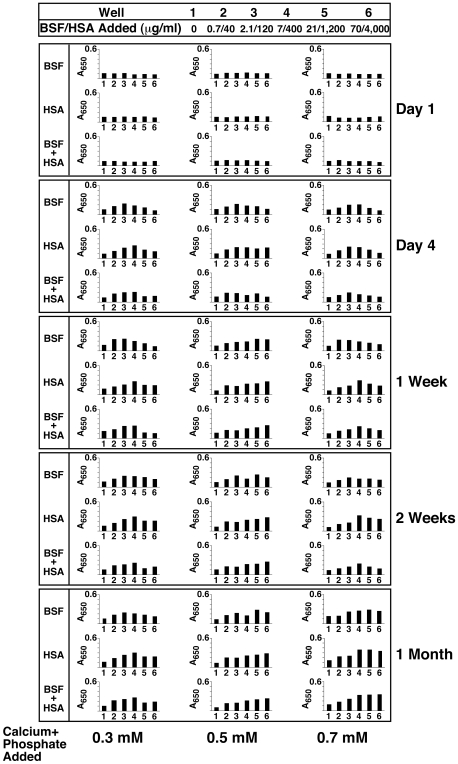
Seeding of NB-like particles by fetuin-A and albumin at low concentrations in supersaturated ionic solutions. Stock solutions of BSF and/or HSA were diluted into DMEM to the relatively low concentrations shown on the top. The precipitating reagents CaCl_2_ and NaH_2_PO_4_ were then added at either 0.3, 0.5, or 0.7 mM, as indicated at the bottom (labeled as “Calcium+Phosphate Added”). Turbidity was monitored by A_650_ turbidity reading following inoculation (“Day 1”) and after incubation in cell culture conditions for the time indicated on the right side of each panel. Bell-shaped curves of precipitation were noticed at “Day 4” for the three different combinations of proteins as well as the three different concentrations of added precipitating reagents. Precipitation in these conditions increased in a time-dependent manner with further incubation. When 0.3 mM of precipitating reagents was used, bell-shaped increases in turbidity were seen for the three protein combinations after 1 month of incubation. With calcium and phosphate added to 0.5 and 0.7 mM, the initial bell-shaped increase in turbidity observed at “Day 4” was seen to shift to the right at “1 Month” reading.

The seeding-inhibitory relationships displayed by both fetuin-A and albumin varied with the protein lots as well as the concentrations tested, which gave a significant margin of error. Nonetheless, the basic seeding and inhibitory influences delineated by these proteins remained unchanged. For instance, Figure 6 illustrates another experiment in which BSF was adjusted between 0.7–70 µg/ml and HSA between 0.04–4 mg/ml. In the presence of 0.3 mM, 0.5 mM, and 0.7 mM calcium and phosphate, these protein ranges gave prominent bell-shaped, dose-dependent increases in turbidity. Turbidity increases could be seen within 4 days of incubation and increased only slightly up to 1 month (Fig. 6). With BSF, at 4 days, turbidity increased with BSF concentrations up to 2.1 µg/ml, decreasing thereafter. Likewise, for HSA, turbidity peaked between 120 to 400 µg/ml of protein concentration and similarly decreased with additional protein input. The protein concentrations seen to produce peak turbidities in this experiment are surely different from those seen elsewhere using different lots of proteins (compare Fig. 6 with 4A). Remarkably, in the experiments depicted in Figure 6, as little as 0.7 µg/ml of BSF was sufficient to induce increased mineral precipitation in the presence of submillimolar amounts of calcium and phosphate (0.3 mM, 0.5 mM, and 0.7 mM; see wells 2 in all 3 columns of Fig. 6).

The lack of any noticeable inhibitory effect on mineral precipitation seen with low doses of fetuin-A (0.7–2.1 µg/ml of BSF) further confirms the notion proposed here that, at low concentrations of fetuin-A, this same calcification inhibitor binds avidly to nucleating apatite complexes, but it is eventually overwhelmed by excess calcium and phosphate, at which point it becomes a “nidus” for further calcification. In fact, as seen in Figure 6, the peak turbidity can be seen to shift progressively toward the right following an increase in the calcium and phosphate concentrations added (compare the various columns). Thus, in the presence of 0.3 mM calcium and phosphate, as little as 2.1 µg/ml of BSF produced peak turbidity, whereas with 0.7 mM calcium and phosphate, the peak turbidity was seen with BSF at 21 µg/ml, a result seemingly confirming the workings of an optimal stoichiometric balance between fetuin-A and its mineral-seeding capability as a function of the total amount of precipitating ions present in any given medium. Beyond these peak concentration levels of turbidity, inhibitory influences could be clearly discerned in both these experiments (see, for instance, wells 4–6 in the 0.3 mM calcium and phosphate column and well 6 in the 0.7 mM calcium and phosphate column).

Assuming further a molecular weight of 48 kDa for BSF, it can be calculated that at 0.7 mM of calcium and phosphate ions, the optimal turbidity produced by 21 µg/ml, or 0.44 µM, corresponds to the binding of 58 apatite units by each BSF molecule. The number of apatite crystals bound to each protein molecule was estimated by dividing the number of phosphate ions present in the well producing maximal precipitation by the number of protein molecules present in the same well as described in the *Materials and Methods*. Phosphate ions were considered to be the limiting factor in the formation of apatite crystals since they were present in lower amounts than calcium ions. We assumed further that every phosphate ion would be part of a crystal unit of apatite under these conditions and that each crystal unit would contain an average of 63 phosphate ions based on earlier estimations [94]. On the other hand, by using an average number of 105 calcium ions for each apatite unit [94] as the basis of our calculation, an estimate of 54 apatite units bound to each BSF molecule was obtained. Either way, the calculations given here correspond at best to only rough approximations since we did not attempt to define more precisely the dose-dependence relationship for BSF insofar as mineral precipitation is concerned. In general, however, our calculated number supports the earlier estimates made by Heiss et al. [94], who established that each molecule of BSF could bind to 10 apatite units for early soluble calciprotein particles (CPPs) or to around 22 apatite units for their so-called “precipitating late” CPPs.

Similar stoichiometric relationships could be defined for albumin, with peak turbidities seen around 0.4 mg/ml (5.88 µM assuming a molecular weight of 68 kDa for albumin) in the presence of 0.7 mM of calcium and phosphate, which would correspond to a stoichiometric binding ratio of 4 apatite units per molecule of albumin. Compared to fetuin-A then, the binding affinity of albumin for apatite is some 14-fold lower. Compared however to the protein concentrations that produce maximal turbidity/precipitation here in the presence of 0.7 mM calcium and phosphate, both proteins are known to be present in the serum at levels that are at least 100-fold higher. Given their predominance in the serum, both proteins are expected to exert a strong inhibitory influence on mineral growth and precipitation even under conditions when the serum is loaded with excess calcium and phosphate. In fact, our own earlier study [3] had already revealed the need for an unusually high amount of calcium or phosphate added to the serum in order for mineral precipitation to ensue.

The turbidity profiles seen in Figure 6 could be sustained largely unchanged for several weeks of incubation except for small shifts in dose-dependence seen with longer incubation times (Fig. 6, see “1 Month” reading). It should also be noted that that these same experiments showed a stronger inhibitory effect associated with albumin as compared with earlier results (Fig. 4A). Nonetheless, both seeding and inhibitory influences are readily apparent for both proteins from the turbidity data shown here. As before, the presence of both fetuin-A and albumin in the same culture wells appeared to produce additive inhibitory effects.

### Heat Treatment of Fetuin-A and Albumin as a Means to Induce Conformational Changes and to Trigger the Seeding of Minerals: Differences Seen between Metastable and Supersaturated Calcium and Phosphate Solutions

The seeding effects seen with both fetuin-A and albumin, manifested only in the presence of exogenously added calcium and phosphate, but not with metastable medium, were initially puzzling. The results would seem to indicate that the calcium and phosphate ions found in the DMEM may be stably complexed with other ionic and organic moieties, such that the added presence of fetuin-A and/or albumin is probably not sufficient to perturb this equilibrium. Viewed from a different perspective, it can also be inferred that the calcium (1.8 mM) and phosphate (0.9 mM) concentrations present in DMEM are not sufficient to saturate either fetuin-A or albumin, however little amount of either protein is added, such that the calcification-inhibitory potential associated with either one of these proteins might be overcome. Apparently, in the presence of additional calcium and phosphate ions added to the medium, there is now the binding of these excess free precipitating ions by these two proteins that, upon reaching saturation, may become the nuclei or nidi for the growth of apatite crystals.

To confirm this notion and to further correlate the results obtained for fetuin-A and albumin with those obtained earlier for whole serum with respect to nanoparticle formation, we next subjected both proteins to heat treatment for different lengths of time, following the same protocol used earlier with whole serum. We reasoned first that there is a fundamental difference seen with metastable and supersaturated calcium and phosphate solutions insofar as the mineral-seeding potential of fetuin-A and albumin is concerned that may be related to the conformational states of these protein molecules. We reasoned further that harsh treatments like boiling may induce the conformational changes required to unfold fetuin-A or albumin that in turn may allow either one or both to induce mineralization in a metastable medium like DMEM.

**Figure 7:**
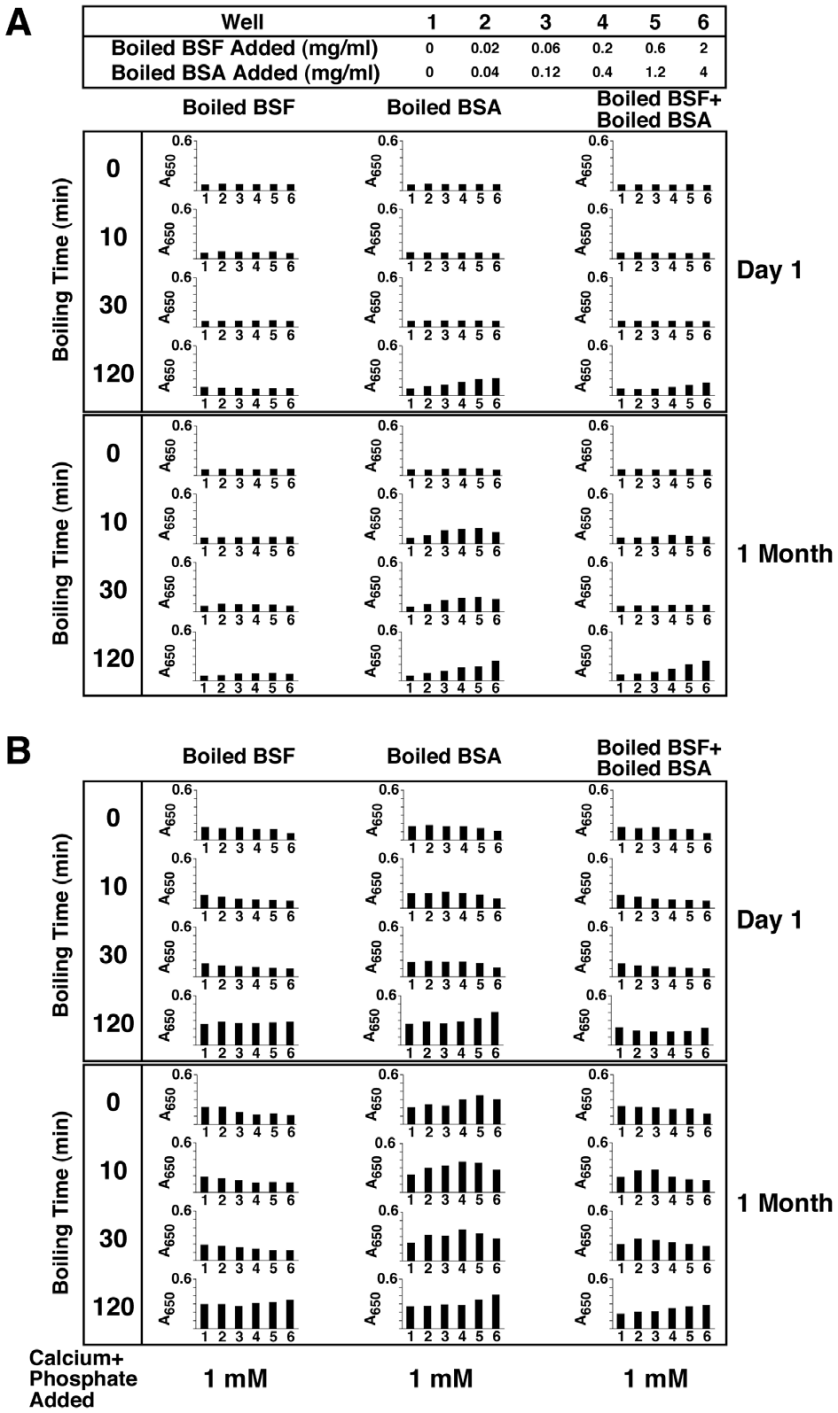
Seeding of NB-like particles by boiled fetuin-A and albumin in metastable versus supersaturated medium. (A) Metastable medium: BSF and bovine serum albumin (BSA) were boiled for the time indicated on the left side. The boiled protein solutions were then diluted into DMEM, separately or together, to the final concentrations indicated on the top. A_650_ was then monitored following inoculation (“Day 1”) and after incubation in cell culture conditions for 1 month, as indicated on the right. Inoculation of either untreated BSF or BSF that had been boiled for either 10, 30, or 120 min did not result in any turbidity changes after 1 month of incubation. In contrast, pre-boiled BSA produced either bell-shaped or linear turbidity changes depending on the amount of boiling time. When added together, pre-boiled BSF and BSA produced additive effects. (B) Supersaturated medium: BSF and BSA were added to medium as in (A) that was also inoculated with 1 mM each of CaCl_2_ and NaH_2_PO_4_. Notice the various patterns of turbidity changes associated with the three protein combinations. See the text for further details.

In Figure 7, both BSF and BSA were boiled for either 10, 30, or 120 min, followed by inoculation into DMEM in the absence (Fig. 7A) or presence (Fig. 7B) of exogenously added calcium and phosphate. Up to 2 mg/ml of BSF and 4 mg/ml of BSA were used for the experiments described here. As can be seen from Figure 7A, BSF that had been boiled for either 10, 30, or 120 min did not give any precipitation when inoculated into DMEM alone, in the absence of any added calcium or phosphate. In the same series of experiments, it could also be noticed that the addition of control, untreated BSF to DMEM also did not produce any precipitation (Fig. 7A, row labeled as “0 min Boiling Time”), in line with the earlier experiments that failed to detect any precipitation under these conditions (compare with the data shown in Figs. 4A and 5). On the other hand, pre-boiled BSA produced precipitation that could be readily detected following 1 month of incubation (Fig. 7A, “Boiled BSA” column). Precipitation appeared to be a function of the boiling time, with a 120-minute boiling treatment resulting in an immediate dose-dependent precipitation detectable on the first day following inoculation, presumably due to drastic protein denaturation. In fact, following this prolonged heat treatment (120 min), the BSA suspension became turbid and appeared to contain fine aggregates. After the first day, the turbidity of the inoculated medium did not seem to increase significantly when measured again after 1 month (Fig. 7A). On the other hand, boiling BSA for 10 or 30 minutes did not appear to create any turbidity increase, as evidenced by the turbidity measurements collected on the first day following inoculation (Fig. 7A). Turbidity however increased gradually over a period of 1 month, following a bell-shaped, dose-dependent curve, similar to what had been seen earlier with pre-boiled FBS (compare with Fig. 3A). That is, turbidity increased up to 0.4 mg/ml of BSA and decreased thereafter with higher protein concentrations. With both whole FBS and HS, we had also seen earlier evidence of protein denaturation with prolonged boiling for 120 minutes; a turbidity increase could also be seen readily on the first day following inoculation (Fig. 3A).

When inoculated together, BSF and BSA that had been pre-boiled for either 10 or 30 minutes no longer produced any precipitation even when observed after 1 month of incubation, suggesting in inhibitory influences exerted predominantly by BSF at the expense of BSA (Fig. 7A, “Boiled BSF+Boiled BSA” column). On the other hand, the addition of BSF and BSA that had been pre-boiled for 120 minutes also gave a dose-dependent increase in turbidity on the first day following incubation into DMEM, presumably due to the same insolubility of boiled BSA seen earlier. It should be further noted that the addition of both control and untreated BSF and BSA did not produce any turbidity change, in line with earlier results shown in Figures 4A and 5.

Together, these results indicate that BSF that had been boiled extensively retained its inhibitory effects on mineral precipitation in a metastable medium like DMEM, while pre-boiled BSA appeared to seed mineral growth. The seeding induced by pre-boiled BSA was replaced however by an inhibitory effect that was seen at higher BSA concentrations exceeding 1.2 mg/ml (results seen with BSA pre-boiled for 10 and 30 in). However, upon prolonged boiling for 120 minutes, BSA lost both its slow mineral-seeding as well as inhibitory potential.

In the presence of added calcium and phosphate, however, the following interesting observations were obtained (Fig. 7B). Firstly, with the addition of 1 mM calcium and phosphate alone, precipitation appeared in DMEM immediately and gradually increased for several days, after which it remained unchanged for up to 1 month (Fig. 7B, only “1 Month” reading shown). In fact, the readings obtained for “Day 4,” “Day 7,” and “1 Month” were virtually indistinguishable (data shown only for 1 month). The addition of control, untreated BSF produced a noticeable dose-dependent, inhibitory effect on this same precipitation that remained also unchanged for a period of observation of up to 1 month (Fig. 7B, row referred as “0 min Boiling Time”). Surprisingly, the inoculation of BSF that had been pre-boiled for 10 or 30 minutes resulted in a similar stable, dose-dependent inhibitory effect that also remained unchanged for at least 1 month. No additional precipitation that could be attributed to control or pre-boiled BSF was found. In contrast, BSF that had been pre-boiled for 120 minutes displayed markedly different behavior. Precipitation was enhanced in a dose-dependent manner by this boiled BSF that synergized with the precipitation produced by the addition of 1 mM calcium and phosphate. Apparently, the calcification-inhibitory effects attributed to BSF were at least partially removed by this extensive boiling.

Pre-boiled BSA produced marked different results. Firstly, both control, untreated BSA and BSA that had been pre-boiled for 10 or 30 minutes were shown to produce an immediate inhibitory effect on the precipitation mediated by 1 mM calcium and phosphate added to DMEM (Fig. 7B, see the “Boiled BSA” column in the “Day 1” panel). Precipitation then increased gradually over the course of the next 3 days (not shown). With additional incubation, the initial inhibitory effects seen associated with both untreated and pre-boiled BSA gave way to a marked dose-dependent increase that peaked at 1.2 mg/ml BSA (Fig. 7B). Pre-boiled BSA produced higher levels of turbidity as compared with control, untreated BSA (Fig. 7B). Still, in all 3 instances (untreated BSA, or BSA that had been pre-boiled for 10 or 30 min), a somewhat inhibitory effect was seen at a BSA concentration of 4 mg/ml. In contrast, BSA that had been pre-boiled for 120 minutes also showed an immediate precipitation on “Day 1” that synergized with the precipitation induced by 1 mM calcium and phosphate alone; this same precipitation did not increase thereafter (compare “Day 1” and “1 Month” panels in Fig. 7B), indicating that, as before, the turbidity seen associated with this form of boiled BSA most likely resulted from protein denaturation, and this denaturation may have removed both the seeding and inhibitory potentials of this protein.

Finally, adding both pre-boiled BSF and BSA to supersaturated DMEM containing 1 mM calcium and phosphate produced the same additive inhibitory effects seen earlier with this type of protein mixture (Fig. 7B, compare with similar column in Fig. 7A). As before, it appears that the presence of BSF, be it untreated or pre-boiled, markedly reduced the seeding potential attributed to BSA alone.

Our results indicate that, compared to albumin, fetuin-A is more resistant to boiling and that it is predominantly a calcification inhibitor even in the presence of exogenously added calcium and phosphate (e.g. in supersaturated calcium solutions). Thus, even upon prolonged incubation for at least 1 month, these same inhibitory effects seen with pre-boiled fetuin-A against supersaturated solutions remained intact and appeared to predominate over any seeding tendencies. Albumin, on the other hand, was shown here to be less inhibitory on mineralization and it also appeared to be more sensitive to boiling. Thus, boiled albumin seeded minerals spontaneously, in the absence of exogenously added calcium and phosphate. However, it is clear that both albumin and fetuin-A, upon being boiled up to 30 minutes, retained their respective mineralization-inhibitory capabilities, a property that was lost upon more extensive boiling (120 min), at which point, seeding prevailed.

From our data, it can be further concluded that we have been able to induce calcium nanoparticle seeding in a metastable medium like DMEM with boiled albumin, but not with fetuin-A. It should be pointed out that our initial intention was to use boiling as a tool to unfold or denature both proteins so as to demonstrate their mineral seeding capabilities in the context of a metastable medium. However, in spite of repeated efforts to unfold or denature fetuin-A using boiling as well as other treatments detailed below, we have failed to induce mineral seeding with fetuin-A under metastable conditions.

For instance, we attempted to immobilize either albumin or fetuin-A, or both, onto various plastic substrates, including 24-well plates and 96-well ELISA plates made of polystyrene or polyvinylidine fluoride (PVDF) membranes. Earlier studies had shown that several proteins in solution tended to inhibit the precipitation of calcium phosphate whereas the same proteins promoted the nucleation of calcium phosphate minerals when they were adsorbed onto a solid substrate [97]–[100]. This dual effect was attributed to the possibility that the protein in solution could cover entirely the nascent minerals, thus masking their growing sites and thereby inhibiting crystal growth [99], [101]. On the other hand, adsorption onto a substrate would prevent the protein from covering the growing crystals, but would instead expose chemical groups needed for the formation of the first crystal nucleus [99], [101]. In the case of albumin, this protein was shown to induce collagen mineralization when present in solution at low concentrations whereas higher concentrations inhibited mineral formation [81], [82]. Albumin also promoted the mineralization of HAP when this mineral was coated with the protein and immersed in metastable solutions [83], [84]. In contrast to these studies, other reports have shown that albumin would essentially inhibit precipitation of calcium phosphate irrespective of whether it was adsorbed onto a substrate or remained free in solution [102]–[112]. From these observations, it thus remained unclear how these proteins would behave when immobilized under conditions known to favor the growth of the putative NB in culture.

In order to verify the effect of immobilized albumin and fetuin-A on the formation of mineral nanoparticles, we attempted to immobilize both proteins onto a hard surface with the intention of inducing the conformational changes required to induce mineral formation. Immobilization was performed by either drying the proteins onto the plates overnight, by incubating the protein solutions overnight at 4°C, or by using cross-linking reagents like poly-lysine or octadecyltrichlorosilane (OTS) to maximize the immobilization of the proteins on the plate. A wide range of protein concentrations ranging between 20 µg/ml to 40 mg/ml was used. Coating was ascertained by washing the plates subsequently with water or HEPES buffer and staining the plates for protein (*Materials and Methods*). The wells coated with proteins were put in contact with DMEM and incubated for several weeks in cell culture conditions, similar to the culture of NB. In spite of repeated attempts, none of these treatments resulted in precipitation of particles directly from DMEM, even after prolonged incubation up to 1 month and monitoring with both A_650_ readings and direct microscopic examination using phase-contrast microscopy (data not shown). From these observations, it appeared that the presence of fixed fetuin-A or albumin was insufficient to induce the formation of mineral nanoparticles in metastable solutions. Since albumin, but not fetuin-A, that had been boiled could indeed induce mineral formation in metastable DMEM, while neither one was able to induce mineralization when fixed onto solid substrates, it is unclear whether our negative results here are due to the particular experimental conditions used. More experiments are clearly needed before a definitive conclusion can be reached regarding the seeding capability of either protein, especially in the case of fetuin-A.

### Association of Fetuin-A and Albumin with the Mineral Phase of Nanoparticles

The seeding experiments shown here, comparing either untreated or boiled fetuin-A versus albumin in the presence of small submillimolar amounts of calcium and phosphate added to DMEM, demonstrate remarkable differences in dose-dependence, with fetuin-A showing much greater inhibitory activity than albumin. Along the same vein of observation, seeding appeared to be triggered at much lower doses of fetuin-A as compared to albumin. A functional inhibition assay developed in an earlier study also showed a 15-fold difference in inhibition between BSF and HSA (0.3 µM versus 6 µM, respectively) when the seeding experiment was carried out in aqueous solutions in the presence of 3 mM calcium-carbonate-phosphate ions (ref. [2]; see Fig. 19 of that study), a result that approximates the 14-fold difference seen with seeding experiments done in DMEM in the present study (Figs. 5–7). In that same earlier study [2], both BSF and HSA were seen to bind to mineral complexes in a stoichiometric manner that could be demonstrated by means of sodium dodecyl sulfate polyacrylamide gel electrophoresis (SDS-PAGE; see Fig. 21 in ref. [2]). It is noteworthy that BSF and HSA that had been trapped by precipitating minerals gave similar intensities when analyzed by SDS-PAGE in spite of a 10-fold difference in their concentrations used, indicating again a much greater affinity of these mineral complexes for fetuin-A as compared to albumin (see Fig. 21 of ref. [2]). These results point to a hierarchy of protein binding by nascent apatite complexes that in turn can be predicted by the binding affinities of the individual proteins toward calcium and phosphate. Furthermore, they suggest that proteins present during particle formation may tentatively become an integral part of calcifying nanoparticles, a notion that had been advocated earlier to explain the biology of NB ([1]–[3], [46]; see also refs. [113], [114] for related studies).

**Figure 8:**
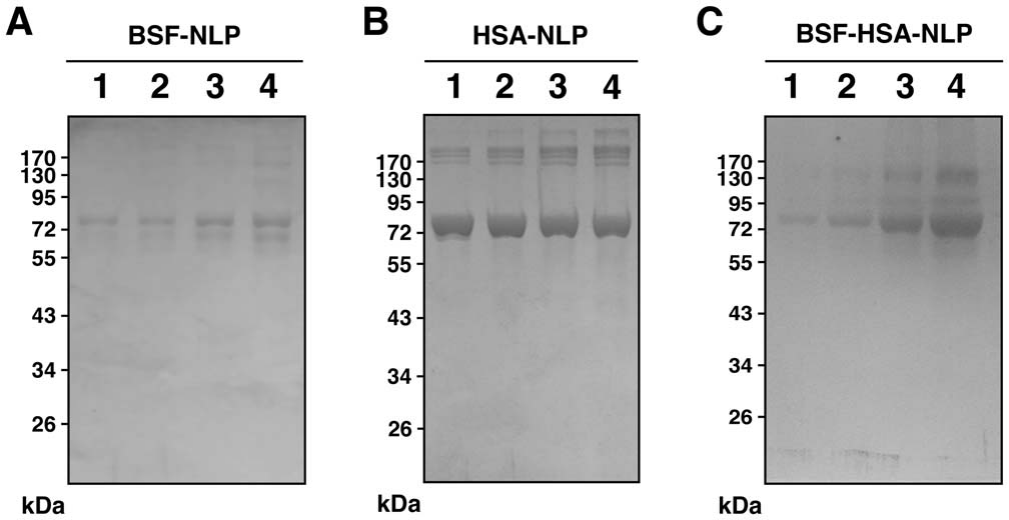
Differences in the binding affinities between fetuin-A and albumin toward NB-like particles as revealed by SDS-PAGE. NB-like particles were prepared from 3 mM each of CaCl_2_ and NaH_2_PO_4_ in 1 ml of DMEM containing either BSF, HSA, or both proteins at the following concentrations: 20 µg/ml, 40 µg/ml, 80 µg/ml, or 160 µg/ml of BSF, corresponding respectively to lanes 1–4 in (A); 0.2 mg/ml, 0.4 mg/ml, 0.8 mg/ml, and 1.6 mg/ml of HSA, respectively, lanes 1–4 in (B); or both proteins at these same concentrations for lanes 1–4 in (C), respectively. Following incubation in cell culture conditions for 1 month, the NB-like particles were pelleted by centrifugation, washed twice in HEPES buffer, resuspended in 50 µl of 50 mM EDTA, and processed for SDS-PAGE as described in the *Materials and Methods*. In the case of BSF-NLP shown in (A), fetuin-A appeared as a major band slightly above 72 kDa (lane 1), with additional bands of higher and lower molecular weights noticeable at higher protein concentrations (lanes 2 to 4). In HSA-NLP (B), albumin formed a major band of strong intensity at 72 kDa. Note a higher molecular band above the 170 kDa marker that appears to increase steadily from lanes 1 through 4, while the 72 kDa band in the 4 lanes appears to decrease slightly in intensity from left to right. In the case of BSF-HSA-NLP (C), note the increase in the intensity of bands at 72 kDa and at a higher molecular weight. The bands of higher molecular weights may be due to altered migration or aggregation of the purified proteins used.

To further ascertain that fetuin-A and albumin bind to calcium phosphate in a stoichiometric fashion and that these two proteins interact with each other by competing for apatite binding, we performed the following SDS-PAGE analysis. Specifically, we sought to use SDS-PAGE to demonstrate the marked differences in binding affinities between these proteins and apatite. Mineral complexes were formed in the presence of BSF, HSA, or both in DMEM inoculated with 3 mM calcium and phosphate as before; they were then washed and subjected to SDS-PAGE (Fig. 8A–C; see also *Materials and Methods*). To facilitate referencing of the nanoparticles studied, we used in the Figure caption the acronym NLP, preceded by the name of the particular protein(s) used to form them (BSF, HSA, or both, described respectively in Figure 8A, B, and C). The NLP samples studied here were obtained by diluting fetuin-A and/or albumin into DMEM at the final concentrations of 20–160 µg/ml for BSF and/or 0.2–1.6 mg/ml for HSA, followed by the addition of the calcium and phosphate ions to a final concentration of 3 mM. Incubation was done in cell culture conditions for 1 month, during which time mineral precipitates appeared gradually that were then sedimented, washed, and processed for gel electrophoresis and Coomassie blue staining.

In the case of BSF-NLP, an increase in protein band intensity was seen in direct proportion to the amount of BSF present in the incubation medium leading to mineral precipitation (Fig. 8A). Fetuin-A appeared initially as a single band slightly above 72 kDa (Fig. 8A, lane 1), but additional higher and lower bands could be distinguished when higher amounts of protein-mineral precipitate were electrophoresed under the same conditions (Fig. 8A, lanes 2–4). It should be pointed out that even though fetuin-A has a predicted molecular weight of 48 kDa, it is known to migrate to a higher molecular weight under denaturing and reducing conditions similar to what were used here, an observation that had been attributed earlier to the heavy glycosylation of this protein [115]. Furthermore, we have found that the migration of fetuin-A may vary with the commercial source of the lot as well as with the particular electrophoresis conditions used (for example, see Fig. 14 of ref. [2] for comparison). Notably, the higher molecular weight bands seen associated with fetuin-A (Fig. 8A, lane 4) were probably the result of aggregation in the presence of apatite, especially since control, untreated fetuin-A failed to show them (data not shown).

In the case of HSA-NLP, obtained using a 10-fold higher concentration of protein as compared to BSF-NLP (0.2–1.6 mg/ml for HSA vs. 20–160 µg/ml for BSF), all albumin-mineral samples analyzed showed a major band of 72 kDa. A dose-dependent increase in the intensity of this major band was not apparent, indicating that saturating doses of albumin must have been used here. On the other hand, with an increase in albumin input used for the formation of HSA-NLP, there was a noticeable dose-dependent increase of higher molecular weight bands (over 150 kDa) that appeared to correspond to aggregated/polymerized protein species (Fig. 8B, lanes 1–4). In fact, we chose to use these saturating amounts of albumin in order to assess more clearly the inhibition produced by fetuin-A, as follows.

Figure 8C depicts the gel profile of various amounts of BSF-HSA-NLP formed in the presence of both BSF and HSA in the same amounts described above. There was a noticeable decrease in band intensity as compared with Figure 8B, indicating that albumin incorporation into the mineral complex had been significantly reduced. Since albumin had been used in excess in these experiments and exceeded the amount of fetuin-A by at least 10 fold, these results indicate clearly that the binding of fetuin-A to the mineral particles was not only significantly stronger than that seen for albumin, but that its binding to apatite appeared to compete off or inhibit the simultaneous binding by albumin. Nonetheless, it is also clear that both BSF and HSA appeared to have been incorporated into the particles, since the gel pattern seen with BSF-HSA-NLP appeared to incorporate characteristics of both BSF-NLP and HSA-NLP (compare the 3 gel profiles shown in Fig. 8). However, it is obvious from inspecting this gel profile that albumin only appeared to incorporate into the same mineral complexes at the higher doses used (0.8 and 1.6 mg/ml, lanes 3 and 4, Fig. 8C), whereas at the lower doses (0.2 and 0.4 mg/ml, lanes 1 and 2, Fig. 8C), only fetuin-A binding appeared to be more noticeable. Note also that in the presence of fetuin-A and albumin, the higher molecular weight bands appeared to migrate differently from those obtained with either protein alone—a result that perhaps suggests in further interactions between the two proteins when they were present together in the same complex. Given however that the two proteins migrated with overlapping molecular weights, the interpretation given here must be viewed with caution and must be considered along with the bulk of the functional data presented in the earlier sections in this study and elsewhere [2].

The data presented here show further that, on the one hand, proteins like fetuin-A and albumin appear to inhibit the formation of calcium nanoparticles, and, yet, they eventually incorporate into the same mineral nanoparticles that they attempt to inhibit in the first place. Moreover, our results clearly indicate that there is indeed a hierarchy in terms of preferential incorporation of proteins into the nanoparticle scaffold, a preference that is dictated by the differential binding affinities displayed by the proteins themselves. Fetuin-A, for one, binds more strongly to apatite minerals than albumin [2], [74], and thus it is a much more potent inhibitor of mineralization that in turn seems to interfere with concomitant albumin incorporation. Finally, these binding (inhibitory) processes appear to be transient, and, upon prolonged incubation, there is a gradual overcoming of this inhibition that yields to particle growth and seeding.

### Protein-Mineral Nanoparticles Show Morphological Resemblance to Both Serum Granules and Cultured NB

**Figure 9:**
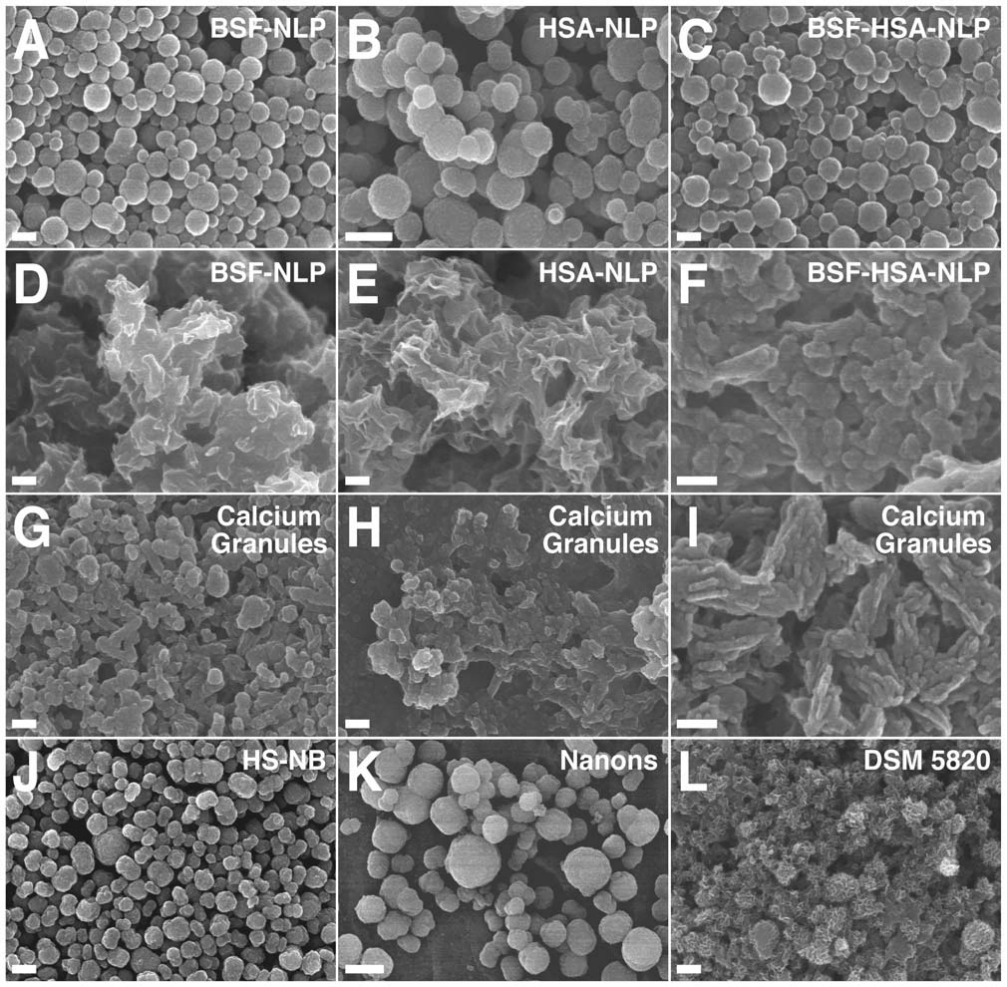
Protein-mineral nanoparticles seeded by fetuin-A and albumin show morphological resemblance to NB and calcium granules when observed by SEM. Protein-mineral nanoparticles were prepared by adding 0.3 mM of CaCl_2_ and NaH_2_PO_4_ to DMEM containing BSF at 2.1 µg/ml (A and D, labeled as “BSF-NLP”), HSA at 120 µg/ml (B and E, “HSA-NLP”), or both BSF and HSA at these same concentrations (C and F, “BSF-HSA-NLP”), followed by incubation for either 3 days (A–C) or 1 month (D–F) in cell culture conditions and preparation of the particles for TEM, as described in the *Materials and Methods*. Protein-mineral nanoparticles containing BSF and/or HSA showed a round morphology when observed after 3 days (A–C), but they tended to coalesce to form films or aggregates when incubated for a longer period of 1 month (D–F). Note the presence of structures resembling cells undergoing cell division in (B). Calcium granules were prepared from the addition of either CaCl_2_ (G), NaH_2_PO_4_ (H), or a combination of both (I) to FBS, followed by overnight incubation, centrifugation, and the washing steps described in the *Materials and Methods*. Calcium granules showed variable morphologies, consisting of either round particles (G) or film/aggregate-like structures (H and I). The morphologies of both the protein-mineral nanoparticles and the calcium granules were similar to NB obtained from 10% HS (J, “HS-NB”) or the NB strains “Nanons” (K) and “DSM 5820” (L) which were both maintained in 10% FBS. These NB samples revealed either round particles (J and K) or more crystalline structures harboring elongated crystal projections and aggregates (L). Scale bars: 100 nm (F, I); 200 nm (A, C–E, G, H, J, L); 400 nm (B); 1 µm (K).

In order to verify whether the mineral nanoparticles formed in the presence of fetuin-A or albumin in DMEM supplemented with exogenous calcium and phosphate (supersaturated solutions) represent particles similar to the purported NB as well as the earlier calcium granules found in the serum [1]–[3], we used a series of electron microscopy techniques with high-resolution capabilities. NLP representative of the experiments described in this study (Figs. 4–6) were prepared by adding 0.3 mM each of calcium and phosphate to DMEM containing either BSF at 2.1 µg/ml (Fig. 9A and D, labeled as “BSF-NLP”), HSA at 120 µg/ml (Fig. 9B and E, “HSA-NLP”), or both BSF and HSA in these same concentrations (Fig. 9C and F, “BSF-HSA-NLP”), followed by incubation in cell culture conditions. These protein concentrations were selected to match maximal mineral precipitation as evidenced earlier from experiments depicted in Figure 6 (0.3 mM column). Samples were then prepared for scanning electron microscopy (SEM) as described in the *Materials and Methods*.

Mineral particles seeded by fetuin-A and/or albumin retained round morphologies that could be seen after 3 days of incubation (Fig. 9A–C; data not shown for shorter incubations). Examined after 3 days of incubation, the particles prepared in the presence of fetuin-A and/or albumin were comparable while showing great heterogeneity in sizes, varying between 50 to 1,000 nm in diameter (Fig. 9A–C). However, we noticed that shorter incubation periods revealed that the fetuin-A particles were in fact smaller in size. Thus, by means of dynamic laser scattering (DLS), we have seen that BSF-NLP freshly formed in the presence of 2 mg/ml of BSF in 1 mM of calcium and phosphate in DMEM gave an average size of 123 nm vs. an average size of 479 nm for HSA at 2 mg/ml incubated under similar conditions (data not shown). In contrast, in the presence of 1 mM calcium and phosphate added to DMEM but in the absence of proteins, the particle size obtained immediately after mixture averaged 1,631 nm. Particle size observed under these conditions was clearly a function of incubation time and by 24 hours at room temperature, the particle sizes of BSF-NLP averaged 227 nm, HSA-NLP 720 nm, and NLP-devoid-of-proteins 4,810 nm (unpublished observations).

With further incubation, particle sizes grew further and while the fetuin-A-particles appeared to lag behind in size as compared with albumin-particles, this difference became less noticeable, and by the 3^rd^ day of incubation, their sizes became somewhat comparable. Moreover, with the passage of time, particles were seen to fuse together, forming structures that resemble dividing bacteria. However, contrary to the earlier assumptions that these formations might represent *dividing* bacteria [4], [5], [7]–[9], it appears more plausible that they form from the growth of two independent mineral particles that *fuse* and *coalesce* as they continue to expand in size. Alternatively, a fully formed particle with rounded shape may presumably also serve as a template for the growth of a second particle and result in formations that may resemble dividing microorganisms.

When particles incubated for the longer period of 1 month were observed by SEM, we noticed that the round mineral nanoparticles had converted into more crystalline films and mattresses, harboring spindles and projection-prone crystals, at which point round particles could either no longer be distinguished or were rare by comparison (Fig. 9D–F, note the differences in morphologies with the corresponding particles shown in Fig. 9A–C).

Particles formed under different mineral:protein ratios showed distinct features, with smaller sizes being seen at higher protein concentrations while films and aggregates predominated with higher mineral-to-protein ratios—with the latter conditions favoring a significantly faster particle-to-film conversion (data not shown).

We compared the morphology of the particles seeded in the presence of proteins with the calcium granules obtained earlier from the serum [3]. These calcium granules were prepared by challenging FBS with either 48 mM CaCl_2_ (Fig. 9G) or 24 mM Na_2_HPO_4_ (Fig. 9H), or, still, with both calcium and phosphate to a final concentration of 2 mM each (Fig. 9I). These calcium granules were shown earlier to display morphologies and chemical compositions identical to the purported NB described in the literature, allowing for the possibility that they may represent universal structures found throughout nature and with potential role in pathogenesis if overtly accumulated in the body [3]. As shown in Figure 9G–I, these same calcium granules showed variable morphologies depending on the amounts of ions added or the time of incubation used, with initial serum precipitations appearing mostly as round nanoparticles with calcium loading (Fig. 9G), and showing more crystalline appearance when either phosphate or calcium and phosphate were added (Fig. 9H and I, respectively), all of which appeared indistinguishable from the protein-mineral complexes studied here. Thus, following incubation, there was a gradual conversion of round particles to spindle and film shapes, a conversion that was evident with both protein-mineral complexes and serum granules.

In order to further ascertain that these protein-mineral particles were similar to the NB described earlier, we used “NB strains” obtained and cultured from several different sources. Thus, “HS-NB” were obtained from healthy human blood by diluting whole serum in DMEM at 10% and incubating the solution in cell culture conditions for two months (Fig. 9J). We also used other cultures of NB obtained initially from 10% FBS and that had been extensively characterized by other authors [5], [8], [9]. In fact, the first “strain” of NB, obtained from FBS, was deposited by Kajander and his colleagues, the discoverers of NB, with the German Collection of Microorganisms (referred as “DSM 5820” in the figures presented in the present study) while another one represented a culture initially named *Nanobacterium* sp. Seralab 901045 by Ciftcioglu and Kajander [85] and later renamed by Raoult et al. as “nanons” [46]. Both “strains” had been maintained in 10% FBS prior to being processed for SEM. NB obtained from 10% HS (Fig. 9J) or the NB strains “Nanons” (Fig. 9K) and “DSM 5820” (Fig. 9L), showed morphologies that varied from round to spindle and film shapes, accompanied by varying degrees of crystallinity. For instance, while the HS-NB showed predominantly smooth round particle shapes, “nanons” were larger and showed surface crystalline ridges, and “DSM 5820” had a mixture of irregular particles and spindles with more irregular crystalline projections. It should be noted however that the particle morphologies seen here could be easily modulated by the frequency of serial passages as well as the amount of inoculated serum. In general, the older cultures appeared more crystalline and film-like while the frequently passaged cultures showed predominantly round particles. From these studies, it can be concluded that, from a morphological point-of-view, the protein-mineral complexes assembled from purified components are virtually indistinguishable from the serum granules and the so-called NB that had been characterized earlier.

**Figure 10:**
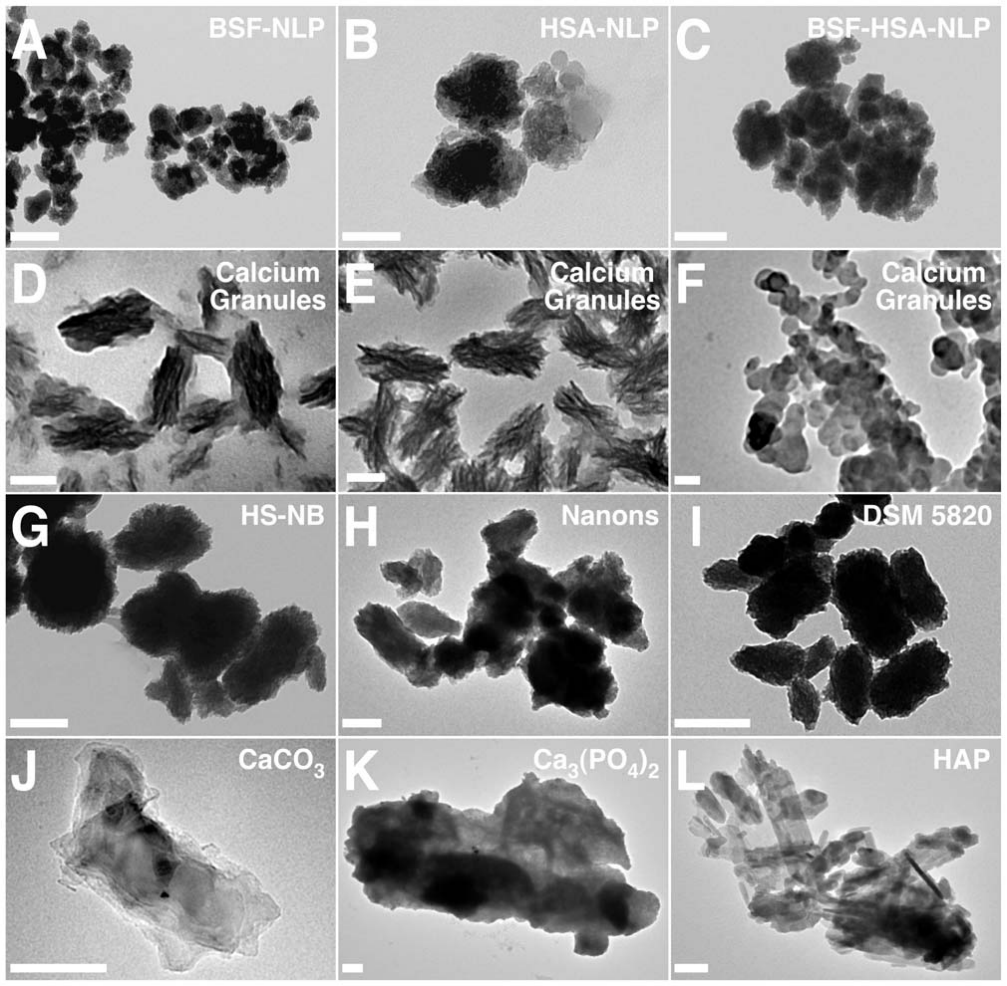
Protein-mineral nanoparticles containing fetuin-A and albumin resemble NB and calcium granules as revealed by TEM. Protein-mineral nanoparticles were prepared as described in Fig. 9, from solutions containing either BSF (A), HSA (B), or both proteins (C), followed by the addition of 0.3 mM each of CaCl_2_ and NaH_2_PO_4_, and incubation for 1 month in cell culture conditions. The samples were then prepared for TEM without fixation or staining. The various protein-seeded particles showed small, rounded formations that tended to aggregate (A–C). The samples of calcium granules prepared from addition of either CaCl_2_ (D), NaH_2_PO_4_ (E), or a combination of both (F) to FBS (see *Materials and Methods*) showed stacks of crystalline spindles and stacks forming ellipsoid structures (D and E), while other granules appeared mostly as round aggregated particles (F). The morphologies of the protein-mineral nanoparticles and the calcium granules were similar to NB obtained from either 10% HS (G, “HS-NB”) or the two NB strains “Nanons” (H) and “DSM 5820” (I) which were both obtained from 10% FBS. In this case, the NB samples consisted of rounded particles with rough surfaces and variable sizes (G–I). Commercially available CaCO_3_ (J), Ca_3_(PO_4_)_2_ (K), and HAP (L) incubated in DMEM for 1 hour showed mainly large, crystalline, monolithic structures. Scale bars: 50 nm (A, F); 100 nm (B, D, E, G–J, L); 200 nm (C, K).

A morphological resemblance between the various structures studied here could be further ascertained by transmission electron microscopy (TEM). Under TEM, BSF-NLP revealed small aggregated particles with rounded shapes. The surface of the particles obtained after 3 days of incubation appeared more crystalline under TEM than when examined earlier by SEM (Fig. 10, compare the surface of the particles shown after 3 days of incubation, which seemed to be irregular and of different electron densities under TEM in Fig. 10A–C whereas the same particles appear to have a smoother surface under SEM in Fig. 9A–C). By analogy, in a recent study on the mineralization of fin from zebrafish in which the exact same sample area was examined by both TEM and SEM [116], the authors also noticed a greater tendency of TEM images of mineral deposits to reveal a more crystalline nature as compared with SEM, similar to what we have also observed with our own NB/NLP samples.

Under TEM, calcium granules showed a higher propensity to form spindles (Fig. 10D–F) as compared to protein-based NLP (Fig. 10A–C). In comparison, the morphologies of both the BSF-NLP and HSA-NLP were remarkably similar to those of NB obtained from either 10% HS (Fig. 10G, “HS-NB”) or the two NB strains “Nanons” (Fig. 10H) and “DSM 5820” (Fig. 10I). Like the protein-NLP, all NB specimens showed rough surfaces with crystalline appearance. NB that had been obtained shortly after serial passages were comparable in sizes and surface characteristics to the protein-mineral complexes studied here and ranged between 20 to 500 nm (Fig. 10G–I, compare the sizes of the particles in Fig. 10A–C).

In support of these observations, the earlier electron diffraction patterns obtained in conjunction with TEM of calcium granules and the purported NB showed a predominance of polycrystalline structures compatible with the presence of amorphous aggregates undergoing varying degrees of crystallization [2], [3]. Virtually identical patterns were obtained with the protein-mineral complexes studied here (data not shown). That is, polycrystalline materials with relatively low degrees of crystallinity could be seen to be associated with BSF-NLP, HSA-NLP, and BSF-HSA-NLP (data not shown).

These results indicate that the simultaneous amorphous-crystalline attributes of NLP appear to represent examples of a wide spectrum of possibilities within the chemical composition repertoire associated with these mineral aggregates. Our results would indicate that the amorphous nature of these particles is enhanced by the presence of proteinaceous factors and possibly other organic compounds found in the serum. Regardless, there appears to be a gradual and irreversible progression or conversion from amorphous to crystalline phases within the same calcified samples. Given the repeated findings of both amorphous and crystalline phases in our samples, along with a wide array of intermediate states, our interpretation appears to offer a reasonable explanation for the assembly of calcifying nanoparticles both *in vitro* and *in vivo*. In fact, the recent breakthrough study using zebrafish fin as a model for biomineralization does point in fact to this same conclusion for mineralization processes seen in living tissues [116].

As additional controls, commercial compounds of CaCO_3_ (Fig. 10J), Ca_3_(PO_4_)_2_ (Fig. 10K), and HAP (Fig. 10L) were similarly incubated in DMEM prior to their being processed for TEM (only data for 1 hour incubation shown here; longer, overnight incubations resulted in a predominance of crystalline, monolithic structures). This additional incubation in DMEM was done to ensure that these control compounds would be subjected to the same culture conditions used here, with the exception that neither whole serum nor serum proteins were present. It should be further noted that DMEM is known to contain a wealth of ions, amino acids, and other compounds needed for the sustenance of cultured cells, exemplifying an environment that is most likely to be found *in vivo*; in turn, the charged and organic substrates contained in DMEM may very well modulate the assembly of nanoparticles! Control samples prepared in this manner (e.g. in the presence of DMEM) showed morphologies that were different from the various protein-mineral complexes, calcium granules, and NB samples shown. Irregular aggregates, platelets, and larger masses of crystals were seen that were devoid however of smaller round nanoparticles (Fig. 10J–L). From these experiments, it would appear that, if round nanoparticles were present in such preparations of commercial compounds, they must be short-lived compared to the other samples examined. It is also possible that in the absence of proteins and other organic compounds found in the serum, the round particles rapidly collapse or coalesce to form larger platelets, films, and aggregates. In fact, earlier studies done by Nylen, Eanes, and Termine had shown that calcium and phosphate solutions, in the absence of any organic compounds, undergo rapid conversion from round particles to platelets and then to larger aggregates within a few hours [117], [118].

### Thin-Section Electron Microscopy of Fetuin-A and Albumin-Containing Nanoparticles Reveal Unique Morphologies

Next, thin-sections of the protein-mineral particles were prepared in order to compare their internal structures with those of the NB-like formations and calcium granules characterized earlier. Protein-mineral particles were prepared by using BSF and HSA as well as precipitating ions at concentrations that favored maximal precipitation as described earlier in Figure 6. In this case, calcium and phosphate were added each at 0.3 mM to DMEM containing either BSF at 2.1 µg/ml, HSA at 120 µg/ml, or both BSF and HSA at these same concentrations, followed by incubation in cell culture conditions and preparation of thin-sections as described in the *Materials and Methods*.

**Figure 11:**
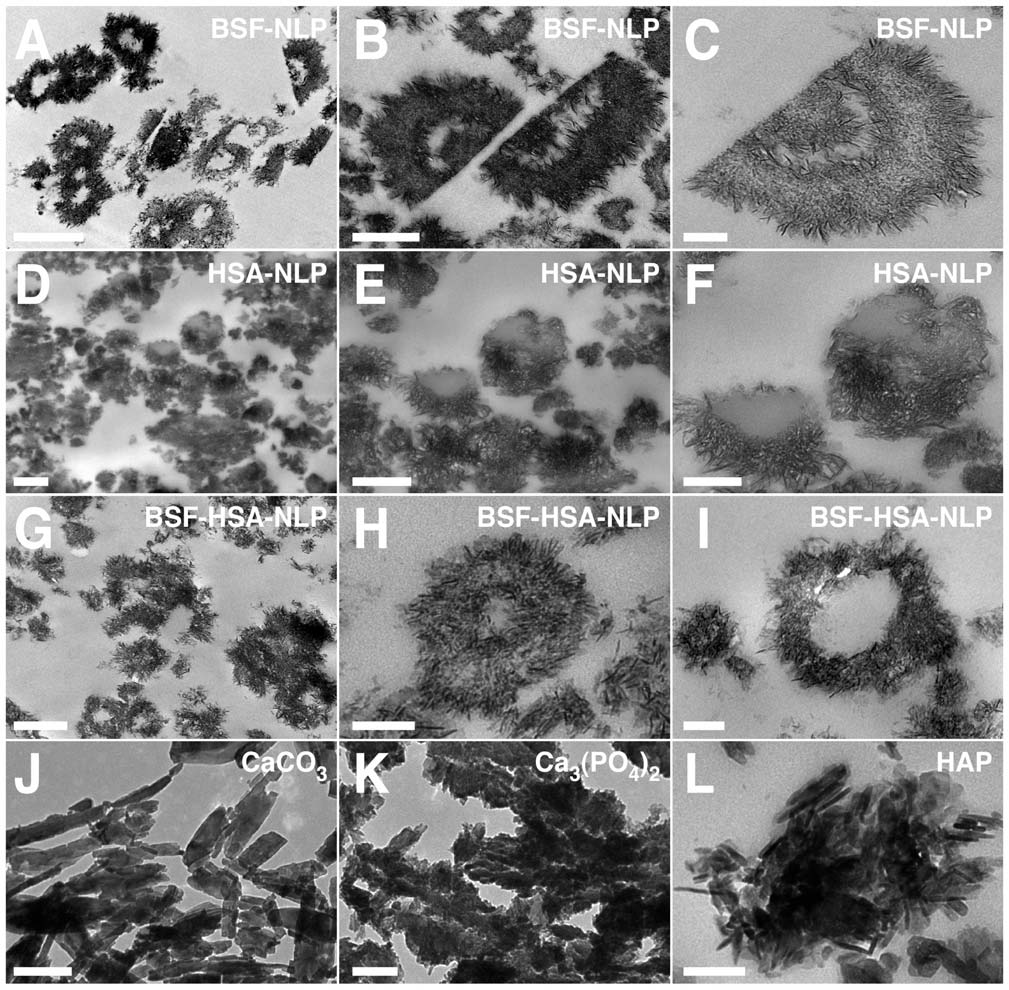
Thin-sections of protein-mineral nanoparticles seeded by fetuin-A or albumin show distinct morphologies. Protein-mineral nanoparticles were prepared as described in Fig. 6, by diluting either BSF at 2.1 µg/ml (A–C), HSA at 120 µg/ml (D–F), or both proteins at these same concentrations (G–I) into DMEM and then adding 0.3 mM each of CaCl_2_ and NaH_2_PO_4_, followed by incubation in cell culture conditions for 1 month. Thin-sections were prepared without fixation or staining, as described in the *Materials and Methods*. BSF-NLP (A–C) resembled multi-layered laminations with alternate electron densities. HSA-NLP (D–F) appeared mostly as incompletely sealed, single-layered formations. The BSF-HSA-NLP particles had rough surfaces covered with elongated crystal projections (G), with some structures appearing either as multi-layered (H) or single-layered (I). Control, commercial grades of CaCO_3_ (J), Ca_3_(PO_4_)_2_ (K), and HAP (L) were incubated in DMEM as in Fig. 10. These controls showed mainly monolithic platelets or aggregates of crystalline formations that contrasted with the round nanoparticles shown above. Scale bars: 100 nm (H, I, L); 200 nm (C, K); 250 nm (F); 500 nm (B, D, E, G, J); 1 µm (A).

As shown in Figure 11, large BSF-NLP around 1 µm in diameter tended to show variegated, multi-layered structures with alternating electron dense and pervious laminations resembling at times “bull's eyes” or “tree-age-ring-like” morphology typical of NB [3], [5], [27] as well as calcium granules found in the serum [3] and throughout nature (see the galleries of granule pictures assembled by Ryall [51]). In this case, the dark rings appeared to consist mainly of crystalline material (Fig. 11B and C). In fact, dark, crystalline needle-like projections could also be seen to protrude transversally from either the surface or from the core interior (see a magnified image in Fig. 11C). Like the many calcium granules found in nature [51], the alternating electron dense and pervious layers found here can also be interpreted as representing intercalated layers of mineral and organic matrices, or alternatively, they may represent distinct stages of nucleation and growth. That is, the multi-ring formations may represent the result of several successive cycles of growth-and-inhibition of the apatite crystals formed in the presence of organic material, in this case fetuin-A. Not surprisingly, inhibitors of calcification like osteopontin, as well as other proteins like alpha-anti-trypsin, were shown to be present at the mineral-protein interface within the multi-layer particles found in kidney tissues [119]–[125].

When albumin was used instead to seed mineral particles in supersaturated solutions, the particles showed morphologies that were significantly different from those of the fetuin-A-NLP seen earlier. Noticeably, HSA-NLP were less likely to form multi-layered structures. Instead, they tended to adopt morphologies resembling incomplete, single-layered rings consisting of electron-dense spindles or needle-like projections surrounding a core material that appeared more electron-pervious (Fig. 11D–F). Assuming that the outer, dark, electron-dense layer represents a mixture of organic and mineral interphases, as interpreted from the vast literature on calcium granules [51], [119]–[126], it is possible that the incomplete rings seen associated with HSA-NLP may represent incomplete enclosure or inhibition of nascent apatite! That is, albumin, being a much less potent inhibitor of mineralization than fetuin-A, may not be capable of inhibiting fully the nucleation of nascent apatite crystals, and this in turn can be translated further into the predominance of incomplete rings seen in Figure 11D–F. Assuming that this reasoning is correct, it can be further inferred that, during the process of nanoparticle assembly, the presence of organic inhibitors actually helps sustain the rounded shapes of nanoparticles, thereby delaying or repressing their conversion into larger aggregates and films.

In comparison, nanoparticles that had been assembled in the presence of both fetuin-A and albumin (Fig. 11G–I) assumed a variety of shapes, including multi-layered formations (Fig. 11G) and single-walled rings (Fig. 11I). Intriguingly, the incomplete rings seen earlier with HSA-NLP were not found among the BSF-HSA-NLP samples. As before, electron-dense, crystalline, needle-like projections could be seen protruding from the rings (Fig. 11H and I). It should be noted that we used combinations of BSF and HSA here at concentrations that favored maximal mineral precipitation as seen in Figure 6, indicating that these images may correspond in fact to optimal protein-mineral interactions that lead to mineralization.

As controls, commercially available CaCO_3_ (Fig. 11J), Ca_3_(PO_4_)_2_ (Fig. 11K), and HAP (Fig. 11L), were each diluted and incubated in DMEM for various amounts of time, and processed for thin-section TEM (only data for 1 hour incubation shown here). As can be seen from Figure 11J-L, these same controls appeared as large homogeneous and monolithic crystalline formations, lacking the characteristic round and multi-layered morphologies linked otherwise to the protein-mineral nanoparticles. Longer incubations of these control compounds in DMEM resulted in further homogenization and consolidation of the aggregates (not shown). These results further indicate that the ring-like structures, be them complete and multi-layered, or incomplete and single-walled, may correspond to organic-mineral interphases that are in turn absent when formed in protein-free medium that had been supplemented with precipitating minerals.

### Chemical Composition of Nanoparticles Formed by Fetuin-A and Albumin in Supersaturated Ionic Solutions

To further study the chemical composition and characteristics of the protein-mineral nanoparticles formed in DMEM containing supersaturated amounts of precipitating ions, we used several types of spectroscopy. Earlier studies had shown that both NB-like specimens as well as serum calcium granules could be characterized as carbonate HAP ([2], [3]; see also the earlier study by Kajander and Ciftcioglu [5]), a type of mineral similar to that found in bones as well as in ectopic calcification [127], [128]. We sought therefore to verify whether nanoparticles containing either fetuin-A or albumin might reveal these same chemical characteristics so as to demonstrate that such simple protein-mineral complexes may indeed reproduce all the structural characteristics attributed to NB and calcium granules.

**Figure 12:**
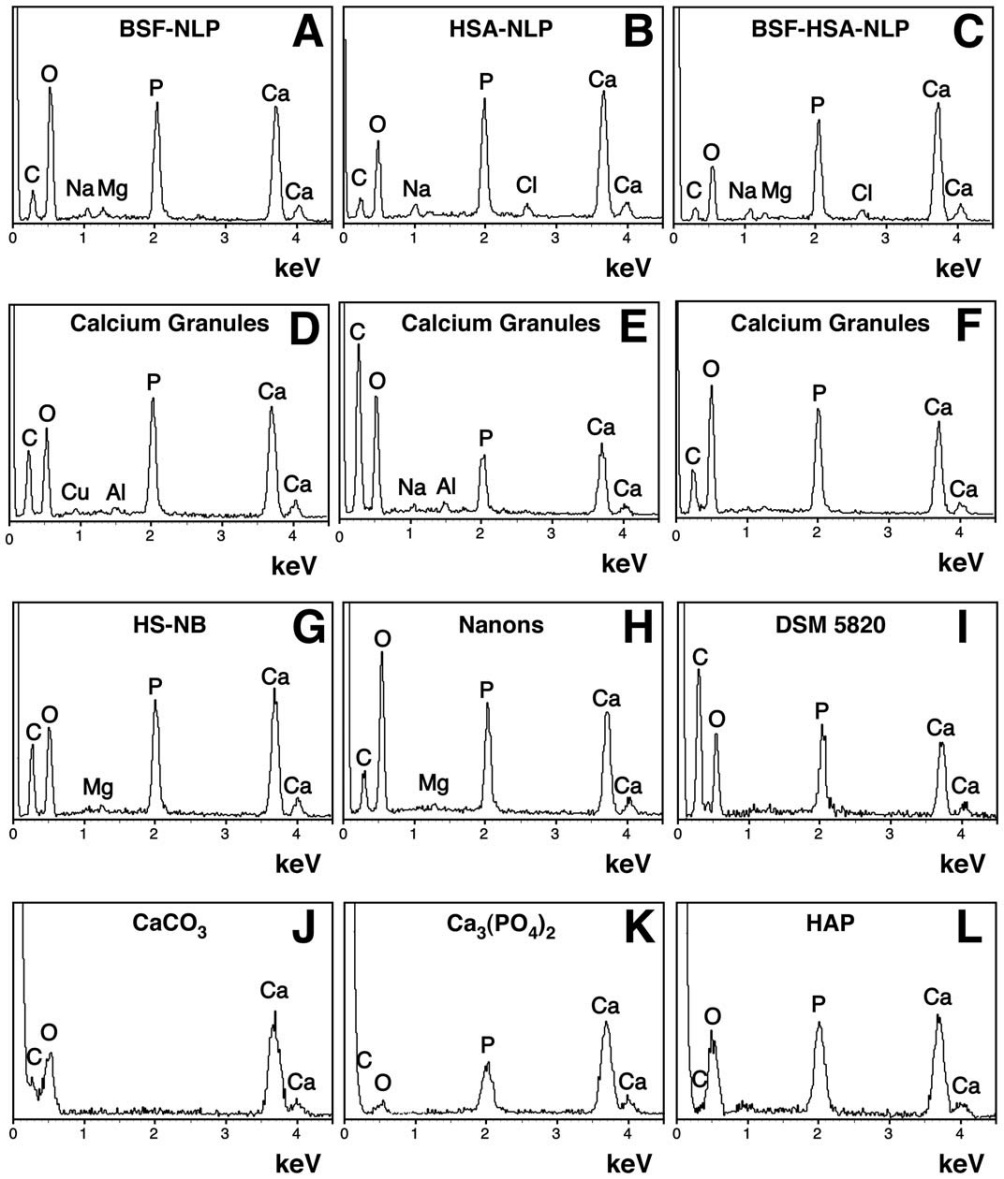
Energy-dispersive X-ray spectroscopy of protein-mineral nanoparticles shows elemental compositions indistinguishable from those of calcium granules and NB. Protein-mineral nanoparticles were obtained as in Fig. 9, from solutions containing BSF (A), HSA (B), or both (C), to which 0.3 mM each of CaCl_2_ and NaH_2_PO_4_ was added, followed by incubation in cell culture conditions for 1 month. EDX spectra were also obtained for calcium granules prepared by adding either CaCl_2_ (D), NaH_2_PO_4_ (E), or a combination of both (F) to FBS, followed by overnight incubation, centrifugation, and washing, as described in the *Materials and Methods*. NB were cultured from 10% HS (G, “HS-NB”) or from 10% FBS (H and I, corresponding to “Nanons” and “DSM 5820”, respectively). In these specimens, major peaks of carbon, oxygen, calcium, and phosphorus were noted, concordant with the presence of a calcium phosphate mineral containing carbonate. The three controls CaCO_3_ (J), Ca_3_(PO_4_)_2_ (K), and HAP (L), diluted and washed in double-distilled water, were shown for comparison. The following Ca/P ratios were obtained: (A) 1.37; (B) 1.53; (C) 1.6; (D) 1.32; (E) 1.48; (F) 1.14; (G) 1.48; (H) 1.4; (I) 1.27; (K) 2.54; and (L) 1.48. Phosphorus was not detected in the CaCO_3_ samples shown in (J).

We first used energy-dispersive X-ray spectroscopy (EDX) in order to determine the elemental composition of the mineral phase associated with the protein-containing particles. As seen in Figure 12, the mineral nanoparticles seeded by BSF and/or HSA (labeled as “BSF-NLP,” “HSA-NLP,” and “BSF-HSA-NLP”) mainly showed peaks of calcium (Ca) and phosphorus (P) (Fig. 12A–C, compare their signals with those obtained for the control samples of CaCO_3_, Ca_3_(PO_4_)_2_, and HAP shown in Fig. 12J, K, and L, respectively).

Peaks corresponding to carbon (C) and oxygen (O) were also observed in the protein-mineral particles, indicative of the presence of carbonate; in fact, in the experiments shown in Figure 12A–C, the oxygen:carbon ratios obtained for these three samples varied between 2.41 to 3.23, close to the ratio of 3 expected for carbonate. The intensities of the C and O peaks also varied greatly with the sample tested as well as with the aging within the same sample. Thus, consistently, we found that fresh samples of BSF-NLP or HSA-NLP contained more carbonate, while aged samples (after 1 week) contained predominantly calcium and phosphate—results that are consistent with earlier findings made on NLP and granules formed in the presence of whole serum [2], [3].

Occasionally, elements like sodium (Na), magnesium (Mg), and chlorine (Cl) were also detected in low amounts (Fig. 12A–C). As such, they can probably be interpreted as minor constituents of the protein-mineral particles, or, alternatively, as unrelated contaminants.

Overall, the EDX spectra obtained for the protein-mineral particles (Fig. 12A–C) were indistinguishable from those of calcium granules prepared in FBS (Fig. 12D–F) or the various NB controls cultured from either 10% HS or 10% FBS (Fig. 12G–I). That is, the main elements present in these samples also consisted of calcium and phosphorus, and to a lesser extent carbonate (Fig 12D–I). The calcium:phosphorus (Ca:P) ratios obtained for the protein-mineral particles varied between 1.37 to 1.60 (Fig. 12A–C) and were similar to the ones found for calcium granules and NB (shown in Fig. 12D–I, with Ca:P ratios varying between 1.14 to 1.48; see also refs. [3], [5]). Carbon and oxygen peaks (e.g. carbonate) as well as minor elements corresponding to copper (Cu) and aluminum (Al) were also noted at times in these samples (Fig. 12D–I).

As additional controls, we also included various chemically defined and commercially available compounds that were incubated and washed in DMEM (Fig. 12J–L). This treatment resulted in a slight widening of the signal peaks as compared to those obtained with the compounds dissolved in water (data not shown; compare with data shown in refs. [2], [3]), but the peak signal strengths were otherwise largely comparable.

**Figure 13:**
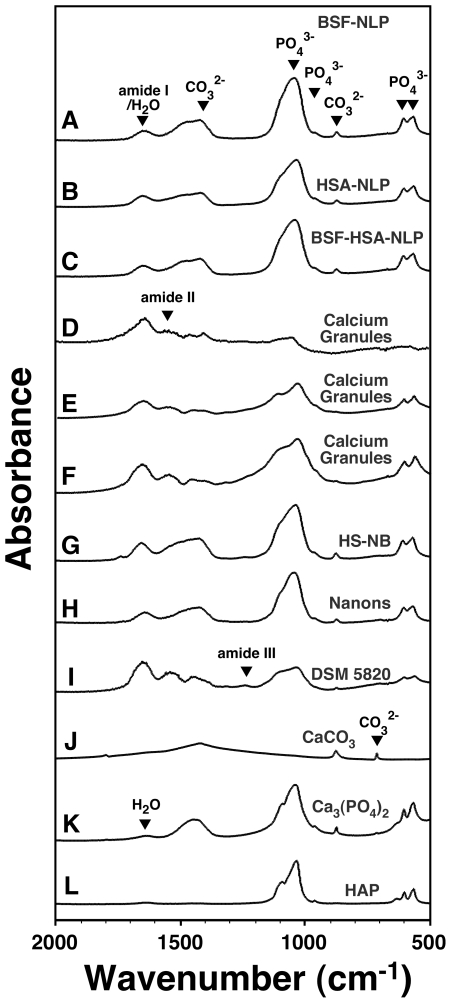
Fourier-transformed infrared spectroscopy of protein-mineral nanoparticles reveals the presence of both carbonate and phosphate. Protein-mineral nanoparticles were obtained as described in Fig. 9, by diluting BSF (A), HSA (B), or both proteins (C) into DMEM, then adding 0.3 mM each of CaCl_2_ and NaH_2_PO_4_, and incubating the solutions in cell culture conditions for 1 month. Calcium granules were prepared by adding either CaCl_2_ (D) or NaH_2_PO_4_ (E) into FBS, or a combination of both CaCl_2_ and NaH_2_PO_4_ (F) into HS, as described in the *Materials and Methods*. NB were cultured from 10% HS (G, “HS-NB”) or 10% FBS (H and I, corresponding to “Nanons” and “DSM 5820”, respectively). The FTIR spectra of the protein-mineral nanoparticles revealed peaks similar to both calcium granules and NB as shown by the presence of phosphate peaks at 575 cm^−1^, 605 cm^−1^, 960 cm^−1^, and 1,000–1,150 cm^−1^ as well as carbonate peaks at 875 cm^−1^ and 1,400–1,430 cm^−1^. Some of the peaks corresponding to phosphate or carbonate were not detected in a few calcium granule samples such as the one shown in (D). In the various nanoparticle samples presented here, peaks corresponding to amide I, II, and III at 1,660 cm^−1^, 1,550 cm^−1^, and 1,250 cm^−1^, respectively, were observed and were attributed to the presence of serum proteins. Spectra for the controls CaCO_3_ (J), Ca_3_(PO_4_)_2_ (K), and HAP (L), diluted and washed in double-distilled water, were included as controls. Residual water (H_2_O) was observed at 1,650 cm^−1^ in some controls prepared in the absence of proteins (K and L); this peak could also have contributed to the intensity of the amide I peak seen at 1,660 cm^−1^ in the other samples shown.

Next, we used Fourier-transformed infrared spectroscopy (FTIR) in order to identify the main functional groups found in the protein-mineral particles, with a view toward assessing the similarities between these particles and the previously characterized calcium granules and NB. For the particles prepared in supersaturated solutions containing BSF and/or HSA, the FTIR spectra showed mainly peaks corresponding to phosphate and carbonate (Fig. 13A–C, compare the signals obtained with those seen in the controls of CaCO_3_, Ca_3_(PO_4_)_2_, and HAP shown in Fig, 13J, K, and L, respectively). Phosphate peaks were observed at wavelengths of 575 cm^−1^, 605 cm^−1^, 960 cm^−1^, and 1,000–1,150 cm^−1^ (Fig. 13A–C; see also refs. [129], [130]). Carbonate groups, on the other hand, were detected at 875 cm^−1^ and near 1,400–1,430 cm^−1^ (Fig. 13A–C; see also refs. [131], [132]). Some peaks seen in the controls were rarely observed in the protein-mineral particles. For example, note the presence of a carbonate peak at 650 cm^−1^ in the CaCO_3_ control shown in Figure 13J that was absent in the protein-mineral particles shown in Figure 13A–C.

In addition to the phosphate and carbonate signals, a peak corresponding presumably to the chemical bonds found in proteins were also detected in the protein-mineral particles. Thus, a peak corresponding to amide I was noticed at 1,660 cm^−1^ (Fig. 13A–C; see refs. [127], [133]). However, we also observed that the same peak corresponding to amide I at 1,660 cm^−1^ might also overlap with a peak corresponding to water that was found at 1,650 cm^−1^ in the control specimens prepared in the absence of proteins (Fig. 13K and L; see also refs. [134], [135]). We noticed that the peaks corresponding to amide II and III at 1,550 cm^−1^ and 1,250 cm^−1^, respectively, were usually not present in the protein-mineral particles, possibly due to the relatively low amount of proteins in these samples (Fig. 13A–C, see also refs. [127], [133]).

The FTIR spectra obtained for the protein-mineral particles (Fig. 13A–C) were similar to those of calcium granules prepared in serum (Fig. 13D–F) or the NB controls cultured from either 10% HS (Fig. 13G) or 10% FBS (Fig. 13H and I). That is, the spectra obtained for these samples consistently showed major peaks of phosphate and carbonate along with peaks corresponding to amide bonds found in proteins. Still, minor differences were noted between these samples. For instance, some samples of calcium granules such as the one shown in Figure 13D appeared to lack peaks of phosphate at 575 cm^−1^, 605 cm^−1^, and 960 cm^−1^ and a peak corresponding to carbonate also went missing at 875 cm^−1^. The phosphate peak observed for this particular sample of calcium granules at 1,000–1,150 cm^−1^ was also of lower intensity (Fig. 13D). In addition, both the calcium granules and NB specimens generally displayed a more prominent amide I peak as compared to the protein-mineral particles (compare the intensity of the amide I peak at 1,660 cm^−1^ in A–C with the same peak shown in D–I). This difference in the intensity of the amide I peak may simply reflect the fact that the calcium granules or NB were either prepared or seeded with whole serum while the protein-mineral particles were assembled in lower amounts of purified proteins. Consistent with this interpretation, the calcium granules and NB specimens displayed additional peaks corresponding respectively to amide II at 1,550 cm^−1^ (Fig. 13D–F, G, and I) and amide III at 1,250 cm^−1^ (Fig. 13I) that were not readily noticed with the protein-mineral samples.

For the control compounds, we also noted the presence of carbonate peaks associated with the calcium phosphate sample (Fig. 13K, where carbonate peaks can be seen at 875 cm^−1^ and 1,400–1,430 cm^−1^). These peaks might be due to the prolonged contact with ambient CO_2_ from air. In spite of these differences, it can be concluded that the protein-mineral particles studied here by FTIR can largely mimic both calcium granules and NB preparations, indicating close if not identical structural relationships between all these three types of entities.

**Figure 14:**
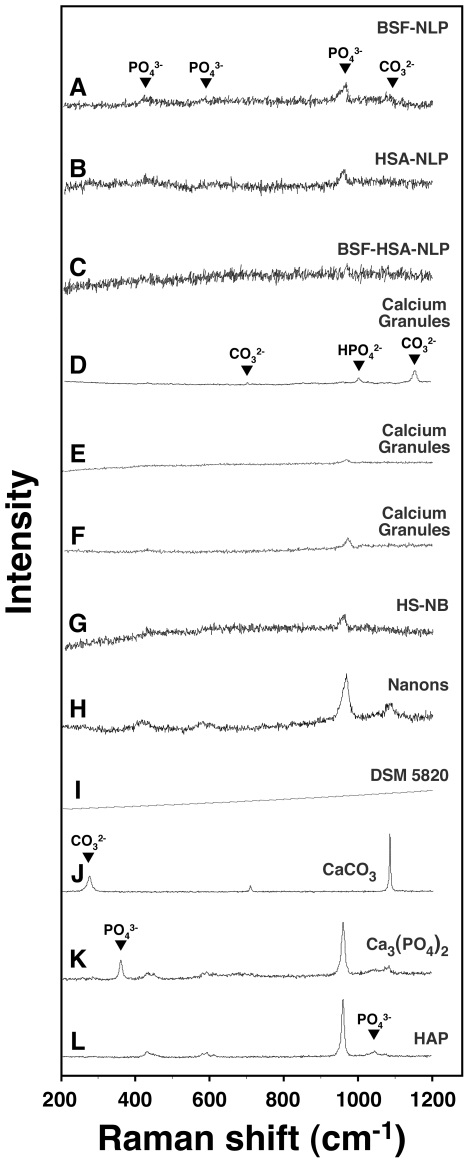
Micro-Raman spectroscopy of protein-mineral nanoparticles shows chemical compositions similar to those of calcium granules and NB. Protein-mineral nanoparticles were prepared as in Fig. 9, by adding 0.3 mM each of CaCl_2_ and NaH_2_PO_4_ to DMEM containing BSF (A), HSA (B), or both proteins (C), followed by incubation in cell culture conditions for 1 month and processing for micro-Raman spectroscopy. Calcium granules were obtained by adding CaCl_2_ (D), NaH_2_PO_4_ (E), or both (F) to FBS, followed by overnight incubation and preparation for micro-Raman spectroscopy as described in the *Materials and Methods*. Micro-Raman spectra were also acquired for NB that were initially cultured from 10% HS (G, “HS-NB”) or 10% FBS (H and I, “Nanons” and “DSM 5820”, respectively). These nanoparticle samples showed phosphate groups at 361 cm^−1^, 440 cm^−1^, 581 cm^−1^, 962 cm^−1^, 1,002 cm^−1^ (HPO_4_^2−^), and 1,048 cm^−1^ and carbonate moieties at 280 cm^−1^, 712 cm^−1^, 1,080 cm^−1^, and 1,150 cm^−1^. The protein-mineral nanoparticles mainly showed peaks of phosphate and lower peaks of carbonate (A–C) while the calcium granules (D–F) and NB (G–I) samples showed carbonate and phosphate peaks of variable intensities. The three controls CaCO_3_ (J), Ca_3_(PO_4_)_2_ (K), and HAP (L), diluted and washed in double-distilled water, were included for comparison.

We confirmed the presence of the major functional groups seen here with FTIR analysis by performing micro-Raman spectroscopy. As can be seen from the micro-Raman spectra shown in Figure 14A–C, the mineral particles prepared in supersaturated solutions containing fetuin-A and/or albumin revealed mainly peaks of phosphate at 440 cm^−1^, 581 cm^−1^, and 962 cm^−1^ as well as carbonate peaks at 1,080 cm^−1^. However, unlike the FTIR spectra and compared to the signals obtained for the control compounds (Fig. 14J–L), there was a clear dampening of signals seen with all the specimens of protein-NLP, calcium granules, and NB—a result that could be due to the presence (and interference) of proteins in the particle scaffold, a possibility that was pointed out earlier [3]. That is, the green laser used to study the Raman scattering effect makes the proteins fluoresce and such fluorescence usually dampens the signal that can be detected from the sample, thereby producing a high background and a low signal-to-noise ratio [127]. Possibly for this same reason, the phosphate and carbonate peaks were often found absent or markedly reduced from the micro-Raman spectra obtained from the various protein-NLP samples, as illustrated by Figure 14B and C, where there is the absence of the carbonate peak at 1,080 cm^−1^ (Fig. 14B) or of any substantial peak (Fig. 14C). Furthermore, some peaks that were present in the controls were rarely seen in the protein-NLP samples (BSF-NLP, HSA-NLP or BSF-HSA-NLP) such as the carbonate peaks seen in at 280 cm^−1^ and 712 cm^−1^ in CaCO_3_ (Fig. 14J) or the phosphate peak seen at 361 cm^−1^ in the Ca_3_(PO_4_)_2_ control (Fig. 14K). As seen earlier during the FTIR analysis, we also noticed that the Ca_3_(PO_4_)_2_ control contained small impurities of carbonate that may have originated from prolonged contact with CO_2_ from air (Fig. 14K, showing a small carbonate peak seen at 1,080 cm^−1^). In the case of calcium granules and NB specimens prepared from serum, these samples also produced variable phosphate and carbonate signals depending on the serum lots used (Fig. 9D–I, see also ref. [3]). For instance, calcium granules prepared in FBS, following the addition of calcium, produced small peaks of HPO_4_^2−^ and carbonate that were rarely seen in the controls (Fig. 14D, see refs. [136], [137] where the peaks found at 1,002 cm^−1^ and 1,150 cm^−1^ were attributed respectively to HPO_4_^2−^ and carbonate). In addition, a few NB specimens like “DSM 5820” did not produce any noticeable signal (Fig. 14G).

In spite of a significant dampening of signals seen here and together with the EDX and FTIR analyses, it can still be inferred that the micro-Raman data show that the nanoparticles prepared in supersaturated solutions containing BSF and/or HSA have a chemical composition largely similar to that of both calcium granules and NB.

**Figure 15:**
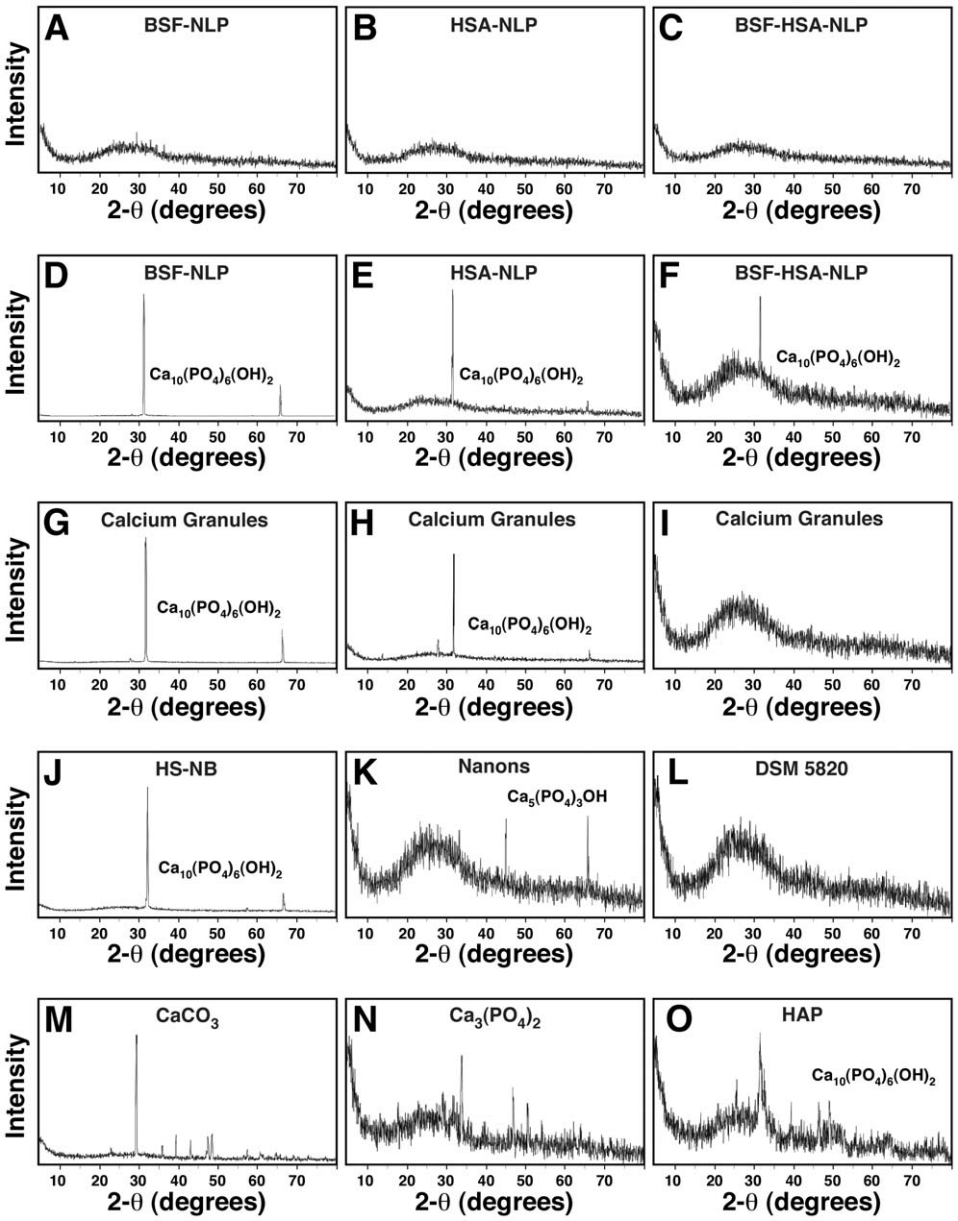
Powder X-ray diffraction spectra of protein-mineral nanoparticles reveal both amorphous and crystalline patterns. Protein-mineral nanoparticles were obtained as described in Fig. 9, by diluting the proteins, separately (A and B) or together (C) into DMEM, followed by addition of the precipitating reagents CaCl_2_ and NaH_2_PO_4_ each to a final concentration of 0.3 mM and incubating the solutions in cell culture conditions for 1 month. The XRD spectra obtained for the protein-mineral nanoparticles obtained after 1 week of incubation represented mainly amorphous patterns as seen by the absence of diffraction peaks (A–C). Longer incubation of 1 month produced protein-mineral nanoparticles with crystalline peaks corresponding to HAP crystals (D–F, Ca_10_(PO_4_)_6_(OH)_2_). XRD spectra were also obtained for calcium granules that were prepared by adding either CaCl_2_ (G) or NaH_2_PO_4_ (H) into FBS or both CaCl_2_ and NaH_2_PO_4_ into HS (I), followed by sample preparation as described in the *Materials and Methods*. Peaks corresponding to Ca_10_(PO_4_)_6_(OH)_2_ were obtained for both calcium granules prepared in FBS (G and H) while the ones prepared in HS usually gave amorphous patterns (I). XRD spectra showing the presence of HAP crystals (J), a calcium phosphate compound (K, Ca_5_(PO_4_)_3_OH), or an amorphous pattern (L) were also acquired for NB cultured in 10% HS for 1 month (J, “HS-NB”) or in 10% FBS for 1 month (K, “Nanons”) or 1 week (L, “DSM 5820”). Commercial grades of CaCO_3_ (M), Ca_3_(PO_4_)_2_ (N), and HAP (O), used as controls, were diluted and washed in double-distilled water.

To complete the chemical analysis of the protein-mineral nanoparticles, we used powder X-ray diffraction (XRD) spectroscopy to study the nature of the crystals found in these samples. For the protein-nanoparticles obtained from supersaturated solutions (BSF-NLP, HSA-NLP and BSF-HSA-NLP), no peak was obtained from the XRD analysis (Fig. 15A–C), indicating a predominance of amorphous phases here. Precipitation of calcium phosphate is usually described to proceed from an amorphous precursor phase which slowly transforms into various calcium phosphate compounds of increased crystalline complexity, ending with the crystalline apatite, which is the more thermodynamically stable end-product [138]. This transition does in fact also occur with protein-NLP, and, upon prolonged incubation or by using lower protein-to-calcium-phosphate ratios, several protein-NLP samples have been shown to progress to crystalline patterns, including the display of classic Ca_10_ apatite signals (Fig. 15D–F). Thus, in the case of these particles, the intensity of the peaks could be modulated by the amount of calcium, phosphate, and proteins added as well as the length of incubation. In general, the spectra will shift more rapidly toward the crystalline phases in the presence of higher amounts of precipitating ions, longer incubations, and lower amounts of proteins. It is likely that the presence of proteins in association with the mineral phase promotes the formation of an amorphous mineral phase that either blocks or slows the transition to apatite, a concept that has been advocated in the past by Mann and other researchers [58].

In support of this notion, the calcium granules prepared in serum in the presence of high concentrations of calcium and phosphate ions also showed variable levels of crystallinity depending on the amount of serum present as well as the amounts of calcium and phosphate added (Fig. 15G–I). Of particular interest is the fact that calcium granules prepared from FBS often displayed crystalline peaks of Ca_10_ apatite, as indicated by the prominent peak at 31.8 degrees on the 2-θ scale (Fig. 15G and H), comparable to that seen with the commercially available HAP powder used for comparison (Fig. 15O), while the calcium granules prepared in HS showed a similar propensity to remain amorphous (Fig. 15I; see also ref. [3] for similar data). This may be attributed to higher protein concentrations associated with HS (averaging 60 mg/ml) compared to FBS (32 mg/ml; see *Materials and Methods*). While the FTIR and micro-Raman analyses suggest that carbonate should be present in the mineral phase of the particles, the signal for carbonate was not detected in our specimens (Fig. 15A–C and data not shown). This result might be due to the absence of this particular diffraction crystalline plane in the samples examined, or alternatively due to the predominance of the HAP signal.

For comparison, it should be noted that the various NB specimens tested also displayed variable amorphous and crystalline patterns (Fig. 15J–L). For example, in one experiment, NB that had been cultured from 10% HS showed complete conversion to the Ca_10_ apatite compound under the conditions tested (Fig. 15J) while other samples of “Nanons” and “DSM 5820” showed either low peaks of calcium phosphate crystals (Ca_5_(PO_4_)_3_OH in Fig. 15K) or only amorphous signals (Fig. 15L). The commercially available controls of CaCO_3_, Ca_3_(PO_4_)_2_, and HAP that had been incubated in DMEM were also included for comparison (Fig. 14M–O).

The presence of both amorphous and crystalline patterns in all the samples seen, including each of the protein-mineral complexes examined, reveals the possibility of a continuous progression from amorphous to crystalline dispositions that in turn can be modulated by the types and amounts of proteins and minerals present. In addition, both the presence of excess proteins (either whole serum or purified proteins like fetuin-A or albumin) and certain competing ions (magnesium or carbonate, data not shown; see also ref. [2]) are now known to result in smaller particle sizes. In fact, the XRD analysis of small mineral nanoparticles is often complicated by the fact that only large crystals over a certain dimension tend to diffract X-rays [139], [140]. Thus, it is not entirely clear whether the nanoparticles studied here are truly amorphous during at least portions of their development or perhaps comprise of small crystals that fail to diffract X-rays.

### Conclusion and Future Perspectives

The results presented in this study further confirm a dual inhibitory-seeding role for serum insofar as the formation of NB-like calcifying particles is concerned, a notion that was first advanced through our earlier studies [2], [3] but that can now be extended to encompass the effects of serum-associated proteinaceous factors and at least those of the two main serum proteins albumin and fetuin-A. That is, calcification-inhibitory factors such as albumin and fetuin-A are seemingly capable of combining with calcium and phosphate to form amorphous nanoparticles, thereby repressing them from progressing further onto crystalline phases that appear in turn as spindles, aggregates, and biofilms. It is only when these same inhibitors are overwhelmed by excess calcium phosphate that mineral seeding will then occur. Since serum and other body fluids are already supersaturated with respect to calcium and phosphate, our results support fully the notion that it is really the de-repression of this same predominantly inhibitory state that results in the seeding of mineral crystals [52]–[58].

Our focus on serum and serum-derived factors as an experimental platform to study the broader issue of nanoparticle assembly can be seemingly justified by both the physiological relevance of this system to biomineralization as well as the known fact that serum has been used in the past as the body fluid of choice for the culture of the putative NB [4], [5], [7]–[9]. These same earlier studies had shown that when FBS and adult HS were inoculated into DMEM at a final concentration of 10%, followed by incubation in cell culture conditions for several weeks, structures described as mineralized NB could be observed in most samples in the form of a white precipitate adherent to the cell culture flask [4], [5], [7]–[9]. Given that this precipitate was absent in DMEM alone incubated in the same conditions, it was concluded that the serum provided the source of putative NB [4], [5], [7]–[9]. Our studies indicate that, in retrospect, the levels of serum used (generally 10% and at times 5%) for the bulk of NB studies were essentially and paradoxically inhibitory on NB formation. In our own studies, as little as 0.1% serum triggered mineral precipitation while the dose-dependent precipitation produced by serum followed typically a bell-shaped curve, with maximal precipitation achieved at 1–3% serum and with FBS generally giving peak precipitations at lower concentrations as compared to HS. The prominent inhibitory effect on mineral seeding shown by serum can also explain now the slow time-course of mineral precipitation associated with the entire NB phenomenology—generally taking several weeks to months for noticeable precipitation to develop. When these observations are factored in, it is now possible to view NB from an entirely different perspective, as follows. In spite of the fact that NB are lifeless mineralo-organic entities, they are nonetheless real and verifiable, and they may in fact represent a general mechanism of calcium homeostasis used throughout nature [2], [3].

It is with these perspectives in mind that we sought to dissect the putative serum factor(s) that might account for the inhibitory-seeding attributes associated with serum. It is not clear for example whether the calcification inhibitors and nucleators found in the serum represent the same exact entities. This latter question is all the more relevant when one takes into account that, presumably, it is the presence of prevailing inhibitory influences that keep supersaturated solutions like serum from calcifying spontaneously [52]–[58]. This is also to say that releasing such inhibitory influences, as accomplished through protease or heat treatment, may be sufficient to trigger calcification in supersaturated solutions. According to Mann, whereas biomineralization can be construed as a result of phase transformation from amorphous to crystalline apatite phases, “‘promotion’ of various phases and polymorphs does not occur in the literal sense, but that the mineralization pathway is controlled through intermittent release of a system under chemical repression” [58].

In fact, our studies point to a definitive role for proteinaceous (e.g. protease and heat sensitive) factors in the inhibition of calcification reactions associated with both bovine and human serum. Indeed, once de-repressed from this same inhibition, be it through protease or heat treatment of serum, it appears that this same supersaturated solution, e.g. serum, will naturally nucleate and precipitate minerals. Accordingly, this same chain reaction—*de-repression of inhibition, followed by calcification*—can be enhanced several fold by the addition of small amounts of calcium and phosphate (0.3–1 mM).

In turn, the addition of these small amounts of precipitating ions (calcium and phosphate) to metastable culture solutions like DMEM has helped unravel attributes associated with protein-linked calcification that are seemingly repressed or subdued in metastable solutions. For instance, fetuin-A and/or albumin inoculated into DMEM alone fail to produce any mineral precipitation. On the other hand, mineral deposition is seen when submillimolar amounts of calcium and phosphate are also added concomitantly, and these ions can be effective even at the low ion concentration levels that normally result in negligible or no precipitation on their own. This synergistic interaction seen between the proteins fetuin-A/albumin and precipitating ions, resulting in apatite nucleation and deposition, further confirms the propensity of these proteins to behave as calcification inhibitors. It is only when the calcium-phosphate equilibrium is perturbed and the apatite-binding capacities of these proteins are somehow overwhelmed through calcium and phosphate loading that mineralization is finally triggered.

The fact that the small amounts of precipitating ions added do not result in any significant calcification on their own but that they do in the presence of fetuin-A and/or albumin, suggests further that the calcium or apatite-binding sites on these proteins may not only bind to excess calcium or apatite, but that they may somehow anchor and bridge the apatite chains so as to allow them to grow in size, thereby forming in effect the *nidi* required for crystallization. Thus, fetuin-A, itself a much stronger binder of apatite than albumin ([74]; see also our apatite-binding calculations discussed earlier), displays not only stronger inhibition of calcification as seen in our experiments, but it clearly synergizes with precipitating ions to produce calcification; however, this seeding effect is seen at much lower protein-to-ion concentration ratios as compared with albumin (Figs. 4–7). In other words, both seeding and inhibition of NB-like particles appear to represent in fact two sides of the same coin, with inhibition seen as the predominant state that nonetheless gives in to seeding when it is somehow de-repressed.

In this sense also it would be reasonable to expect that a stronger calcification inhibitor like fetuin-A, by it binding more avidly to apatite, would be much more likely to be tightly associated with nascent apatite nuclei as compared with other more weakly apatite-binding proteins like albumin—a concept that has been confirmed and extended through the elegant work on primary and secondary CPPs done by Heiss and his co-workers [73], [74], [94], [141]. Our own morphological data indicate that the fetuin-A-mediated mineral particles are more likely to look as multi-walled sealed rings whereas albumin-particles are more prone to resemble incomplete single-walled rings. Presumably, fetuin-A, by it being a more avid apatite binder or inhibitor, is more likely to cover an apatite nucleus entirely, thereby blocking its access to further growth, whereas weaker inhibitors (albumin) tend to produce an incomplete seal, which may facilitate the further seeding and growth of apatite crystals. Nonetheless, the results shown here and elsewhere [2], [3], [73], [74], [94], [141] demonstrate that any such inhibition, be it by a potent inhibitor like fetuin-A or a weaker inhibitor like albumin, is only transient at best and, that, eventually, there is a progressive and irreversible transformation of the round particles to spindles and these to films and aggregates. In full agreement with this notion and as shown by our own spectroscopic and ultrastructural data, when fetuin-A and/or albumin particles are fully developed, they end up becoming virtually indistinguishable from the purported NB or calcium granules grown out of whole serum and other more complex body fluids.

Still in this respect, it should be noted that, to date, all NB-related studies have been done in the presence of body fluids or tissue homogenates inoculated into culture medium (reviewed in refs. [2], [43]). This procedure may very well have introduced small amounts of calcium and phosphate, not to mention inorganic and organic modulators with calcification-inhibitory-and-seeding properties, a situation that may be comparable to the conditions used here with purified proteins, in which addition of exogenous precipitating ions is also required for calcification to develop.

Proteins are present in high amounts in the serum and many of them have been shown to bind to NB-like particles or granules derived from the serum [2], [3]. As far as biomineralization is concerned, proteins are known to modulate the formation, morphology, orientation, and growth of the apatite crystals found in the mineralized tissues of living organisms (see the excellent reviews on this topic in refs. [99], [100], [142]). Given their binding affinities for calcium and apatite, certain proteins may interact with the apatite crystals during the mineralization process and as such they are expected to be enriched in mineralized tissues like bones and teeth. Several questions however remain to be addressed, including whether the same inhibitory-seeding role in calcification seen here with serum and the serum proteins fetuin-A and albumin has been documented elsewhere in the literature. It is also not clear whether the seeding or inhibitory attributes associated with a protein may not in fact be a function of the protein's conformational state, as we presumed to be the case when attempting to unfold fetuin-A and albumin through heat and immobilization treatments described in this study.

As it turns out, both inhibitory and seeding roles have been ascribed to a number of mineralization-related proteins. Table 1 lists the many proteins implicated in mineralization in vertebrates along with their tissue distribution and their (seeding and/or inhibitory) effects on mineralization as seen both *in vitro* and *in vivo*. To address further the role of a protein's conformational state in effecting either inhibition or seeding, we further segregated the *in vitro* experiments referenced in Table 1 into proteins that had been adsorbed onto solid substrates or dissolved in solution. Our summary table confirms that, depending on the conditions used for the studies, e.g. whether the proteins are adsorbed onto substrates or are dissolved in solution, several proteins present in the serum like albumin, fibrinogen, and vitronectin have actually been shown to exhibit both seeding and inhibitory roles *in vitro*. That is, as expected from our own heat treatment studies, these same calcium and apatite binding proteins when adsorbed onto solid substrates express nucleating or seeding tendencies while their counterparts in solution tend to be inhibitory!

**Table 1:** Proteins associated with mineralization in vertebrates along with their tissue distribution, their effect on mineralization *in vitro*, and their potential role *in vivo*.

Protein	Tissue Distribution	Effect *in vitro*, Adsorbeda,b	Effect *in vitro*, Dissolved^c^	Possible Role *in vivo* d	Ref.
Aggrecan (proteoglycan)	Cartilage		Inhibitor		[143]-[145]
Albumin	Blood, body fluids, bone	Inhibitor/nucleator; no effect	Inhibitor/nucleator	Inhibitor	This study [2], [62]–[64], [81]–[84], [102]–[112], [146]–[149]
Amelogenin	Enamel, bone, others	No effect	Inhibitor; no effect		[109], [150]–[153]
Biglycan (proteoglycan)	Bone, connective tissues, teeth	Inhibitor/nucleator	Inhibitor		[102], [154]–[159]
Bone acidic glycoprotein-75	Bone, connective tissues, teeth	Nucleator		Nucleator	[142], [160], [161]
Bone sialoprotein	Bone, dentin	Nucleator	Inhibitor; no effect	Nucleator*	[142], [162]–[170]
Chondrocalcin	Cartilage, retina	No effect	No effect		[165]
Collagen type I	Bone, cartilage, dermis, others		No effect	Structure	[99]
Decorin (proteoglycan)	Bone, connective tissues, teeth	Inhibitor/nucleator; no effect	Inhibitor; no effect		[102], [154]–[159]
Dentin matrix protein-1	Bone, dentin, kidney, others	Nucleator		Nucleator*	[142], [148], [171]–[174]
Dentin phosphophoryn	Dentin	Inhibitor/nucleator	Inhibitor	Nucleator	[99], [142], [165], [175]–[178]
Dentin sialoprotein	Dentin	Inhibitor/nucleator		Nucleator	[178], [179]
Fetuin-A (α2-HS-glycoprotein)	Blood, body fluids, bone	No effect	Inhibitor/nucleator	Inhibitor*	This study [2], [56], [57], [66]–[68], [180], [181]
Fibrinogen	Blood	Nucleator	Inhibitor		[104], [110], [182]
Fibronectin	Blood, connective tissues		Inhibitor/nucleator		[106], [107]
Lithostatin	Pancreas, pancreatic secretion		Inhibitor	Inhibitor**	[56], [183]–[185]
Matrix gla protein	Arteries, bone, cartilage			Inhibitor*	[56], [186], [187]
Osteocalcin (bone gla protein)	Blood, bone, cartilage, teeth	Nucleator; no effect	Inhibitor		[102], [165], [188]–[190]
Osteonectin	Bone, dentin, others	Inhibitor/nucleator; no effect	Inhibitor		[165], [191]–[194]
Osteopontin	Arteries, bone, kidney, others	Inhibitor/no effect	Inhibitor	Inhibitor*	[142], [162], [164], [165], [195], [196]
Prothrombin fragment-1	Blood, urine		Inhibitor	Inhibitor***	[197]
Statherin	Saliva		Inhibitor	Inhibitor	[56], [198]–[200]
Tamm-Horsfall protein	Kidney, urine		Inhibitor	Inhibitor***	[201]–[203]
Uropontin	Kidney, urine		Inhibitor	Inhibitor***	[56], [204]
Vitronectin	Blood, bone		Inhibitor/nucleator		[106]

a The effect of the proteins on mineralization was studied *in vitro* following their adsorption onto solid substrates such as agarose beads, agarose gels, or collagen fibrils.

b The term “Nucleator” refers to a protein which is able to induce mineral formation in a metastable solution where precipitation does not occur spontaneously. An “Inhibitor” consists of a protein which has the ability to delay or prevent mineral formation.

c The effect of various proteins on mineralization was also studied following dissolution of each protein into a liquid buffer.

d The word “Structure” refers to a protein which does not appear to induce or inhibit mineral formation by itself, but which is known to be important for the disposition and the arrangement of minerals formed *in vivo*.

*The potential role of each protein during mineralization *in vivo* was proposed based on gene deletion studies in laboratory animals when available; in this case, the proteins were denoted with a single asterisk. A role for the other proteins shown was proposed based on limited functional studies giving sometimes divergent results; the role of these proteins should therefore be considered with strong reservation.

**Inhibition of pancreatic stones of calcium carbonate.

***Inhibition of kidney stones of calcium oxalate or calcium phosphate.

This Table was adapted and modified from the review by Benesch et al. [100].

We have also noticed that these dual seeding-inhibitory tendencies seen *in vitro* with some proteins may not in fact correlate with their net mineralizing effect seen *in vivo* (Table 1). As for the *in vivo* role of proteins presented in Table 1, a definitive assignment could only be derived from the phenotype of mice that were engineered to lack the gene coding for the protein in question, as already done in the case of dentin matrix protein-1, bone sialoprotein, fetuin-A, matrix gla protein, and osteopontin (see the proteins marked with a single asterisk in Table 1 and the corresponding references therein). However, in the absence of gene knockout data, a potential role for some of the proteins was proposed by the respective authors based on limited functional studies and thus must be viewed with caution. Based on such analyses, for example, albumin has been deemed to be a inhibitor of mineralization *in vivo* [56], [62], and yet this protein can display either inhibitory or nucleating properties *in vitro* depending on the conditions used (Table 1). As for fetuin-A, on the other hand, we were not able to find any study documenting the effect of the adsorbed protein; our own experiments reported here, however, demonstrate that fetuin-A can also behave as a nucleator when subjected simultaneously to heat treatment as well as to challenge with submillimolar amounts of calcium and phosphate.

Surprisingly, few studies to date have addressed the possibility of fetuin-A representing a nucleator of calcium phosphate minerals. In a recent report [141], the early stage of CPP formation was studied based on a dynamic small-angle X-ray scattering analysis. It was observed that fetuin-A at various concentrations (1, 5, or 15 µM) added to 20 mM calcium and 12 mM phosphate ions did not in fact act as a nucleating agent of CPPs, but it essentially inhibited their formation by “shielding” nascent mineral nuclei [141]. Given that relatively high concentrations of calcium and phosphate ions were used in these experiments, it is not clear whether they are in fact representative of the slow and spontaneous formation of NB grown in metastable solutions or of NLP assembled in metastable solutions that had been challenged with submillimolar amounts of precipitating ions, both of which were meticulously addressed here. Other studies have reported that by fetuin-A was effective only in delaying mineral formation temporarily [71], [205]. For instance, when fetuin-A was present at 5 mg/ml in solutions containing 4 mM of calcium and phosphate, precipitation in the form of a fetuin-A-mineral complex appeared only after 4 to 5 days of incubation at room temperature [71], [205]. When calcium and phosphate ions were present at 5 mM in these same conditions, the incubation time was shortened to 20 to 24 hours [71], [205]. These latter observations clearly support our hypothesis of a dual inhibitory-seeding role for fetuin-A.

The dichotomy seen between inhibitory and seeding tendencies as a function of protein solubility (conformational state) is not in fact limited to serum proteins. Thus, Table 1 illustrates that several proteins associated with bones or teeth have also been found to possess a dual seeding and inhibitory role on mineralization, including biglycan, bone sialoprotein, decorin, dentin phosphophoryn, osteocalcin, and osteonectin. These proteins also show marked propensity to act as apatite crystal nucleators when adsorbed *in vitro*, consistent with the assumption that, once bound to collagen fibrils, they may unfold and help nucleate the first crystals required for collagen mineralization *in vivo* [99], [100]. Given the variabilities in seeding versus inhibition seen with the same proteins under different conformational states, it would appear that designating a protein as inhibitor or nucleator can be rather arbitrary. We contend that, in principle, any proteinaceous inhibitor of mineralization can be induced to nucleate under conditions that result in a conformational change in its structure.

With regards then to the differences seen between adsorbed and soluble proteins and still in the context of Table 1, it would appear that apatite-binding proteins tend to act as nucleators of calcification while the same proteins free in solution tend to act as inhibitors, a notion that has also been advanced in several earlier studies [97]–[100]. Presumably, an adsorbed protein may provide binding sites that recruit and bridge ions so as to position them in a specific tridimensional configuration needed to form a mineral nucleus [101]. On the other hand, when free in solution, the protein may still bind to precipitating ions, but it somehow may either prevent them from forming a mineral nucleus or coat the nascent crystal, thereby blocking any growing sites and preventing further growth [99], [101].

For our own experiments, we also attempted to immobilize albumin and fetuin-A onto various substrates and monitored their calcification effects in metastable solutions. Despite repeated trials, we were unable to directly seed mineral nanoparticles under the conditions used. While two earlier studies had reported that albumin could promote the formation and growth of apatite crystals when adsorbed to collagen [81], [82] or to apatite itself, using ceramics comprising of commercial apatite and calcium phosphate [83], [84], it is not clear how these results relate to our own observations given the differences in experimental conditions used. For one, the nature of the substrate used here could have played a role in the absence of effect observed. It is also not clear how the effect of albumin can be ascertained from its immobilization onto apatite or calcium phosphate crystals, which are known to promote the nucleation of other identical crystals by secondary nucleation [118]. In addition, the concentrations of calcium and phosphate used could also have played a role in the different responses obtained. Additional experiments are needed to reconcile these differences.

It should also be noted that although we did not obtain calcification in a metastable medium (DMEM) using either immobilized fetuin-A or immobilized albumin, we were however able to demonstrate seeding with heat-treated albumin (Fig. 7). Heat-treated fetuin-A, on the other hand, failed to seed under the same conditions. The only seeding seen with fetuin-A (also seen with albumin) was in the presence of submillimolar amounts of calcium and phosphate added simultaneously to DMEM. This property may be attributed to the potent inhibitory effect on apatite formation associated with fetuin-A. Albumin, by comparison, being a much less effective inhibitor of apatite nucleation [74], [94], has shown a greater tendency to mineralize under the conditions studied.

Viewed from a different perspective, the differential display of inhibitory versus seeding tendencies may depend on the stoichiometric relationship between the number of protein molecules versus the number of precipitating ions available in any given environment. Thus, our results indicate that, for any calcium or apatite binding protein and for given amounts of calcium and phosphate present in a medium, the protein concentration should certainly influence its effect on mineralization. This relationship can be illustrated by the significant differences seen with the amounts of fetuin-A versus albumin needed to trigger calcification (Figs. 4–7). That is, fetuin-A is such an effective binder of apatite that it will seed apatite minerals not only when the protein-to-mineral ratios are low compared to that seen with albumin but also only with the concomitant addition of submillimolar amounts of calcium and phosphate. Based on these considerations, and extrapolating further to body conditions, fetuin-A should behave as a potent inhibitor of calcification at levels found in the body fluids, while it will nucleate at low protein concentrations only in the presence of excess calcium and phosphate. This is not surprising since according to our calculations, each bovine fetuin-A molecule binds to 54–58 apatite units while each human albumin molecule binds to 4 apatite units. In fact, due to these same stoichiometric considerations, low concentrations of calcium or apatite binding proteins have been generally associated with nucleation of mineral formation whereas high concentrations are known to usually delay or inhibit this same process [100]. In fact, according to Boskey, any macromolecule that can bind and coat nascent crystals will inhibit crystal growth when present in high concentrations in solution [206].

Proteins that inhibit mineral formation are thought to play an important role by preventing spontaneous calcification of the serum and the extracellular fluids [52], [54], [56]. This role is critical given the high natural propensity of these supersaturated extracellular fluids in vertebrates to calcify [52]–[58]. Direct evidence for the role of these proteins in preventing calcification *in vivo* comes from the observations that mice designed to lack any one of the calcification inhibitors like fetuin-A, matrix gla protein, or osteopontin are prone to ectopic calcification and to the debilitating effects associated with this process [67], [68], [186], [196].

While most proteins that can bind to apatite crystals can be seen as inhibitors of mineralization, several proteins listed in Table 1 have been shown to be able to initiate mineral formation as well. Proteins like bone sialoprotein, biglycan, and decorin interact with the collagen fibrils and are thought to initiate the formation of the first mineral nucleus required for collagen mineralization [99], [142]. Collagen fibrils are often described to play a structural role by forming a receptacle which can delineate the main area destined to become mineralized in bones [99], [100]. Proteins can initiate mineralization by lowering the activation energy required for the formation of the first mineral nucleus in metastable solutions which usually does not occur spontaneously [101]. Given that the extracellular fluids are saturated in calcium and phosphate ions [52]–[58], the formation of mineral nuclei is thought to be sufficient to initiate mineralization, which in turn can proceed further on its own by the deposition of calcium and phosphate ions onto the nascent nuclei. It is further assumed that an effective mineral nucleator should provide an array of functional groups, particularly carbonate groups as well as phosphorylated residues, which possess high affinity for calcium and phosphate ions [101], [142]. Accordingly, several studies have suggested that proteins present in the serum may also act as nucleators of calcification. Strong evidence for the presence of a putative nucleator(s) in the serum comes from the observation that demineralized bones incubated in DMEM containing as low as 1.5% serum can remineralize following incubation for a few days [207]–[209]. This remineralization did not proceed if the demineralized bones were incubated in DMEM alone for the same period of time [207]–[209]. The factor responsible for this remineralization, which remains unidentified, was attributed to one or more protein(s) with a molecular weight between 55–150 kDa [207], [209] and which required dephosphorylation by alkaline phosphatase to become activated [210]. At first sight, this suggestion might appear paradoxical given that various systems including proteins are present in the serum to prevent calcification at any given time. Nevertheless, the observation that blood is continually in contact with bones through the so-called basic multicellular unit and bone-remodeling compartment—two anatomical structures that are associated with remodeling of cortical and cancellous bones, respectively [211], [212]—leads to the possibility that a protein secreted by osteoblasts to initiate mineralization in bones might end up circulating in the blood at low concentrations [208], [209]. Were this scenario to occur *in vivo*, it is likely that spontaneous calcification of blood would be prevented as long as the other major inhibitory systems remain in place [208], [209]. Whether or not this putative nucleator(s) plays a role in the formation of NB in cell culture conditions is still unclear.

Still with regards to fetuin-A and albumin, it should be noted that we selected these two proteins for our demonstration of a dual inhibitory-seeding model for protein-mediated mineralization since they represent the two main proteins found in association with NB-like particles [1]–[3], [46]. Their effect on mineral formation has been studied extensively in the past mainly because they are present in high amounts in mineralized tissues. These findings have long suggested that the two proteins might play a role in the mineralization process [56], [57], [81], [82]. Alternatively, the binding of these two proteins to the mineral phase of bones may simply reflect their high affinity for both calcium phosphate and apatite [94]. In support of this alternative view and as noted earlier, proteins from blood are in constant contact with bones and this could account for the gradual enrichment of albumin and fetuin-A in mineralized tissues. Both albumin and fetuin-A are also found at high concentrations in the serum used to culture NB. In fact, albumin represents the main protein found in both the fetal bovine and adult human sera while fetuin-A is more abundant in FBS than in HS [92]–[96]. Both proteins can inhibit or delay the precipitation of calcium phosphate *in vitro* [2], [3], [62]–[64], [66], [110], [111] and both bovine fetuin-A and human albumin have been shown to account for a significant portion of the calcification-inhibitory effect associated with each serum [62], [66]. In fact, earlier studies made on the inhibitory capacity of human serum showed that two-thirds of the inhibitory potential of the serum studied was due to proteins and other macromolecules of high molecular weights while the other one-third of the inhibitory capacity could be attributed to compounds of low molecular weights [62]. Further observations made on albumin-depleted serum indicated that albumin accounted for about half of the inhibitory effect of the high molecular weight fraction present in the serum [62].

Similarly, when fetuin-A-depleted FBS was incubated in DMEM in cell culture conditions, it was reported that a precipitate of calcium phosphate would form in the bottom of the flask within 6 days of incubation [205]. It thus appeared that the removal of fetuin-A was sufficient to induce mineral precipitation from DMEM under these conditions. Given that the DMEM used in this study was also supplemented with phosphate to a final concentration of 2 mM [205], further experiments are needed to evaluate whether these observations are relevant to our own findings with NB and protein-minerals described here. While more experiments are required to integrate and compare the various systems of calcification used here and elsewhere, together, these results strongly support the notion that both fetuin-A and albumin represent major repressors of spontaneous calcification associated with the serum.

The calcification-inhibitory effects associated with fetuin-A and albumin can be attributed to the presence of several calcium and apatite binding sites on these proteins which may presumably sequester calcium and phosphate ions before they bind to the growing crystals [2], [3], [62]–[64], [66], [110], [111]. In addition, the calcium or apatite binding sites may tentatively mediate inhibition by binding onto the growing sites of the nascent crystals and blocking further mineralization. Albumin has been shown to possess as much as thirty calcium-binding sites with three different binding affinities [60] while fetuin-A has been shown to have six calcium-binding sites per molecule with two different binding affinities [65]. The calcium-binding constants associated with the fetuin-A molecule are generally lower than the ones obtained for albumin, an observation which suggests that the binding sites of fetuin-A have a higher affinity for calcium than those of albumin [65]. In addition to the potential influence of calcium-binding sites, the inhibitory effect of these proteins on mineral formation can also be attributed to the presence of specific protein motifs which promote the interaction of the proteins with growing crystals. Indeed, fetuin-A possesses a motif consisting of a cystatin-like domain with several basic amino acids which has been shown to interact with calcium phosphate and apatite crystals [66], [94]. The removal of this motif on fetuin-A by mutational analysis showed that it was responsible for the relatively strong inhibition effect of this protein [66], [94]. While no specific mineral-binding motif has been described for albumin, the interaction of this protein with apatite crystals has been attributed instead to the multiple calcium-binding sites present on this protein [62]-[64]. The possibility that a protein conformation might be associated with the inhibitory effect of albumin was also suggested by the observation that heat-denatured albumin showed increased inhibitory effect compared to the native protein [62], a result that we could not confirm however through our own experiments. Given that these two proteins are present in relatively high amounts in the serum, it is possible that they would play a predominant role in the inhibition effect seen during the culture of NB or during the assembly of NLP in the body. Taken together, these earlier studies suggest that the high inhibitory effect of albumin may be attributed mainly to its relatively high concentration in the serum [62], [74] while the stronger inhibitory effect of fetuin-A may be attributed in turn to its strong interaction with apatite crystals [74], [94].

In the context of biomineralization, perhaps no other protein has given more divergent and even contradictory results in terms of seeding or inhibition than albumin. These discrepancies were more noticeable when studies were done via adsorption of albumin to various solid substrates of well-defined chemical composition. For instance, albumin had earlier been shown to promote the nucleation of calcium phosphate crystals when adsorbed onto a substrate, but to essentially act as an inhibitor of mineral deposition when dissolved in solution [83]. In these experiments, albumin was adsorbed onto ceramics of apatite and calcium phosphate and immersed into a buffer containing high concentrations of calcium and phosphate similar to those contained in the serum [83], [84]. Precipitation of calcium phosphate, verified by SEM and FTIR, was found to be more prominent when albumin was adsorbed on the surface [83], [84]. However, other authors have reported that albumin seemed to exert an inhibitory effect or a lack of nucleating effect irrespective of its being adsorbed to titanium or agarose beads or its being dissolved in solution [102], [105]. The discrepancy seen between these observations has been attributed to the nature of the substrates which may seemingly have produced different protein conformations [83], [84], [149]. As such, the presence of a specific albumin conformation which allows for both firm adsorption to a surface and binding of calcium from the solution is thought to be required for albumin to induce mineralization [83], [149]. In another set of experiments, the presence of albumin in solution has also been described to promote the mineralization of phosphate ceramics [84]. In this case, the presence of a carbonate-buffered solution was found to alter the charge characteristics of albumin and to increase mineralization of phosphate ceramics when compared to solutions where no albumin or carbonate buffer was used [84]. In yet another study, the dual inhibitor-nucleator effect of albumin described here could also be seen in the context of collagen type I-mediated mineralization and was shown to depend on the concentration of albumin used [81]. When albumin was present at low concentrations (below 10 mg/ml) in supersaturated solutions containing 3.6 mM of calcium and 2.7 mM of phosphate ions, it appeared to exert a nucleating effect on collagen mineralization and to lead to increased crystal growth whereas high concentrations (above 10 mg/ml) essentially inhibited mineral formation [81]. Albumin also had an effect on the induction time needed for mineralization to start, extending the period of time for mineral to appear when compared to solutions where albumin was absent [81]. Accordingly, the disparate results may be attributed to several factors which can include the nature of the mineralization assay used, the provenance, purity, and characteristics of the protein used, as well as the different criteria used to describe its mineralizing function [82], [100].

Together, these studies illustrate the complexities as well as the subtleties seen in terms of seeding and inhibition functions that can be attributed to a single protein, all of which may depend on both the conformational changes as well as the concentrations of the same protein found in any given compartment. These inherent attributes of calcification-related proteins make it difficult if not altogether impossible to classify the respective roles of the same proteins during the mineralization process without first considering their physiological context (see also the excellent reviews in refs. [82], [100]).

Since we were not able to produce NB-like minerals using only fetuin-A and/or albumin inoculated into a metastable medium like DMEM, without first heat-inactivating these proteins and adding exogenous calcium and phosphate, it is possible that other serum factors may be involved in the formation of the NB-like particles seen here and in the earlier studies. Lipids had earlier been shown to be associated with NB-like particles derived from the serum (ref. [2] and unpublished observations). Preliminary experiments show that, in addition to proteinaceous factor(s), lipids in the form of membrane or matrix vesicles (MV) may actually be involved. MV are small lipid-bound vesicles released by the vast majority of cells, but in the context of collagen-independent mineralization, they are especially significant in that they are known to be actively released by skeletal cells like osteoblasts, chondrotonblasts, and odontoblasts, and are in turn capable of nucleating the deposition of calcium phosphate in specific areas of the body (see the comprehensive review by Anderson et al. [213]). The release of these vesicles is associated with the mineralization of bones, cartilage, and teeth [213]. In bones, the release of MV is thought to be associated with mineralization away from collagen fibrils in the endochondral plaque [214]. Ectopic calcifications are thought to be associated with the release of MV-like vesicles by vascular smooth muscle cells which experience insults of high concentration of phosphate ions [215], [216]. The mechanism of mineral deposition by matrix vesicles is thought to involve the presence of calcium and phosphate channels in the membrane delineating MV; these channels gradually increase the concentration of calcium and phosphate ions within MV until mineral precipitation ensues [213]. In the context of NB formation, while our earlier studies have failed to provide conclusive evidence for a role of lipids in this process [2], we are now able to extract lipids, in the form of MV, directly from serum samples and use them to seed NLP *in vitro* (data not shown). These same membrane vesicles have earlier been shown to be found in the blood and in most if not all body compartments [213], [217]. In this context, it should be noted that the earlier study by Cisar et al. [44] had in fact shown that lipids like phosphatidylinositol are indeed capable of seeding apatite particles that are morphologically similar to the putative NB, thereby providing a direct link between lipid moieties and the seeding of apatite nanoparticles in a metastable medium like DMEM.

A yet another recently proposed mechanism of mineralization that needs to be considered in the context of NLP formation involves the hydrolysis of polyphosphate present in dense calcium phosphate granules seen in tissues experiencing mineralization [218]. Presumably, this hydrolysis can be catalyzed by alkaline phosphatase [218].

Finally, it is not clear whether the protein-mineral nanoparticles and NLP described here are related in any way to the so-called proteons and proteon-nucleation centers (PNCs) previously shown to represent blood-derived, metallic nanoclusters associated with serum protein fragments such as hemoglobin alpha-chain [219]. It is also not clear whether the more recently described proteon-associated, cell-like nanoforms in the blood that can be produced by hypotonic hemolysis are in any way related to the nanoparticles or their underlying assembly mechanisms described here [220].

Mineralization inside the body is thought to occur through the formation of an amorphous phase of calcium phosphate which gradually converts into the more stable apatite ([221]; see also ref. [116] addressing current controversies surrounding this topic). Our own data indicate that much of this amorphous-crystalline phase transformation can be mimicked through *in vitro* studies of the kind reported here. Our studies indicate that the protein-mineral particles assembled using fetuin-A or albumin are in many respects similar to the particles described earlier in association with NB and calcium granules. Our results point to an orderly and successive morphological conversion from spherical nanoparticles to spindles, and these to films, that are comparable despite the diverse environments in which these same particles and aggregates are found. The binding of proteins and possibly other organic compounds to nascent apatite is what apparently sustains the particles in their spherical conformations, while the growth of apatite crystals will eventually de-repress this same inhibitory influence resulting in the fusion of the round nanoparticles allowing them to coalesce to form spindle and film-like shapes. Whether this general progression of morphologies is restricted to calcium phosphate or may be conceptually extended to other minerals in general remains to be explored.

## Materials and Methods

### Culture of NB from Serum

NB specimens were cultured from FBS or HS as described before [5], [85]. Approval for the use of human samples in this study was obtained from the Institutional Review Board of Chang Gung Memorial Hospital (Gueishan, Taiwan, Republic of China). Written informed consents were signed by the individuals who provided blood samples. Human blood was obtained from healthy volunteers by venipuncture following sterilization of the skin with alcohol. The blood was withdrawn into sterile Vacutainer tubes containing no anticoagulant (Becton, Dickinson & Company, Sparks, MD, USA). Whole blood was centrifuged at 1,500× g for a period of 15 min at room temperature. The supernatant corresponding to HS was retrieved and placed into another tube. The FBS (Biological Industries, Kibbutz Beit Haemek, Israel; PAA Laboratories, Pashing, Austria) and HS used throughout this study were sterilized by filtration through both 0.2-µm and 0.1-µm membranes (Pall Corp., Ann Arbor, MI, USA) prior to use. To culture NB, both sera were diluted into DMEM (Gibco, Carlsbad, CA, USA) to final concentrations ranging from 0.1% to 10%. Culture was performed in 24-well plates with flat-bottom wells and covering lid (Corning, Inc., Corning, NY, USA) using a final volume of 1 ml per well. Culture was also performed in 75-cm^2^ flasks with 0.2-µm vented caps (Corning) using a final volume of 20 ml per flask. Untreated DMEM was used as a negative control. The culture plates and flasks were incubated at 37°C for several months in the humidified atmosphere of a cell culture incubator. *Nanobacterium* sp. strain DSM 5820 was obtained from the German Collection of Microorganisms and Cell Cultures (DSMZ; Braunschweig, Germany). Culture of “nanons”, which was initially called “*Nanobacterium* sp. strain Seralab 901045” [46], [86], was kindly provided by Dr. Didier Raoult (Unité des Rickettsies, Centre National de la Recherche Scientifique UMR 6020, Faculté de Médecine, Marseille, France). Both NB strains DSM 5820 and “nanons” were originally isolated from commercially available FBS used for cell culture purposes [86].

To prepare NB samples for electron microscopy and spectroscopy analyses, the cell culture medium of a flask containing a 1-month-old culture of NB was discarded and the NB sample which consisted of a white precipitate adherent to the flask was scraped using a sterile cell scraper (Corning). The precipitate was resuspended in 1 ml of DMEM and centrifuged at 16,000× g for 15 min at room temperature. The pellet was washed twice with DMEM, HEPES buffer (20 mM HEPES, 1 mM CaCl_2_, 2 mM Na_2_HPO_4_, 0.02% sodium azide, and 0.15 M NaCl, pH 7.4), or double-distilled water using the same centrifugation steps. The NB specimen was resuspended in a small volume of double-distilled water and used for the microscopy and spectroscopy analyses.

### Photography and Spectrophotometry

Images of the 24-well plates used throughout this study were obtained using a scanner operating in the reflective light mode (Scan Maker 8700, MicroTek, Hsinchu, Taiwan) as described earlier [2]. Spectrophotometry readings of 24-well plates were performed at 650 nm using a Spectra Max M2 spectrophotometer (Molecular Devices, Sunnyvale, CA, USA), essentially as described [2]. Throughout this study, photographic and A_650_ turbidity readings referred to as “Day 1” were taken within one hour following the preparation of each 24-well plate.

### Culture of NB-Like Particles from Protease-Treated Serum and Boiled Serum

Stock solutions of porcine pancreas trypsin (Sigma, St-Louis, MO, USA) or bovine pancreas chymotrypsin (Sigma) were prepared in water at a concentration of 5% (w/v). The protease solutions were sterilized by filtration through a 0.2-µm membrane. The NB-like particles depicted in Fig. 2 were prepared by adding trypsin or chymotrypsin into FBS or HS at a final concentration of 0.5% (v/v), followed by incubation at 37°C for 2 hours. A final volume of 1 ml of serum was used. Following incubation, the solution was diluted to final concentrations ranging from 0.1% to 10% (v/v) in DMEM and the mixture was incubated in cell culture conditions for several weeks. Alternatively, this experiment was repeated by treating FBS or HS with trypsin or chymotrypsin that had been boiled at 95°C for 1 hour. As a negative control, the stock solutions of trypsin or chymotrypsin were diluted into DMEM to final concentrations ranging from 0.01% to 3% (v/v) prior to incubation. The DMEM used for these experiments contained 0.02–0.2% sodium azide in order to prevent contamination.

To culture NB-like particles from boiled serum as shown in Fig. 3, HS was first diluted to 25% (v/v) using double-distilled water. FBS and 25% HS solutions were boiled at 95°C for 10, 30, or 120 min. The boiled sera were then diluted into DMEM to final concentrations ranging from 0.1% to 10% (v/v) and the solutions were incubated in cell culture conditions for several weeks. In some instances, stock solutions of 0.25 M CaCl_2_ and NaH_2_PO_4_ (both at pH 7.4) were successively added at a final concentration of 1 mM prior to incubation. The pH of the stock solution of 0.25 M CaCl_2_ was adjusted to 7.4 with 1 M HCl or 1 M NaOH whereas the pH of the NaH_2_PO_4_ solution was adjusted to 7.4 with 0.25 M Na_2_HPO_4_. These solutions were also sterilized by filtration through a 0.2-µm membrane prior to use. Parallel experiments were also conducted by adding aliquots from 0.25 M NaHCO_3_ pre-adjusted to pH 7.4, to the same final concentrations as those of CaCl_2_ and Na_2_HPO_4_. Results were virtually identical compared to experiments without carbonate. For brevity, only the data for the addition of calcium and phosphate are shown in the present study.

### Seeding of NB-Like Particles by Fetuin-A, Albumin, and Serum in Supersaturated Solutions

Stock protein solutions of BSF and HSA were prepared by dissolving the protein in HEPES buffer at a concentration of 10 mg/ml, followed by filtration through 0.2-µm filters. Several lots of proteins were used for the experiments described in this study. For Fig. 4, BSF and HSA were obtained from AppliChem (Boca Raton, FL, USA); for Fig. 5, BSF and HSA were from Sigma; and for Fig. 6, BSF was from Sigma while HSA consisted of a sterile solution used for intravenous injections (Plasbumin®-25; Talecris Biotherapeutics, Inc., Research Triangle Park, NC, USA). The stock solutions of proteins were then diluted into DMEM, individually or in combination, to concentrations varying between 0.7 µg/ml and 40 mg/ml. Other cell culture media obtained from Gibco were used in parallel, including Roswell Park Memorial Institute 1640 or RPMI-1640, F12 medium, medium 199, Glascow minimum essential medium, and Leibovitz L-15 medium. In some experiments, the precipitating reagents CaCl_2_ and NaH_2_PO_4_ were added to final concentrations varying between 0.1 mM to 1 mM. The final solution volume used was 1 ml. The solutions were then incubated in cell culture conditions for several weeks. The concomitant addition of carbonate, as outlined in the previous section, yielded similar results and data for such experimens are not shown here.

To culture NB-like particles from boiled protein solutions as shown in Fig. 7, solutions of BSF purified from FBS (Sigma) or BSA purified from FBS (Sigma) were prepared in HEPES buffer at a final concentration of 25 mg/ml. The protein solutions were filtrated through 0.2-µm membranes prior to use. These protein solutions were boiled at 95°C for 10, 30, or 120 min. The boiled protein solutions were diluted in DMEM at final concentrations ranging from 0.02 mg/ml to 2 mg/ml for boiled BSF and from 0.04 mg/ml to 4 mg/ml for boiled HSA. In some experiments, the precipitating reagents CaCl_2_ and NaH_2_PO_4_ were added successively each at a final concentration of 1 mM to the DMEM solutions containing proteins. The solutions were incubated in cell culture conditions for several weeks.

In order to evaluate the possibility that adsorbed proteins nucleate NB-like particles, BSF (Sigma), BSA (Sigma), or HSA (Talecris) were used in adsorption experiments. Adsorption of the proteins to polystyrene 24-well plates was performed by covering each well with 250 µl of protein solution at concentrations varying between 20 µg/ml to 10 mg/ml. Similarly, 250 µl of FBS and HS were also used at concentrations varying from 0.1% to 10%. The plate was incubated at 4°C overnight. Following incubation, the protein solution was removed and each well was washed twice with 250 µl of DMEM. We verified that the proteins used were adsorbed to the plate by staining the wells with 250 µl of Coomassie blue diluted 1∶5 in double-distilled water (Dye reagent concentrate; Bio-Rad Laboratories, Hercules, CA, USA). After 10 min of incubation, the shift to blue color was monitored directly by visualization of the plate and compared to controls without proteins which did not produce the blue color. Following adsorption of the proteins, 1 ml of DMEM was deposited in each well and the plate was incubated in cell culture conditions for several months. Alternatively, the protein solutions were deposited in each well and left to dry overnight under a laminar flow hood. Each well was washed twice with DMEM. 1 ml of DMEM was pipetted into each well and the plate was incubated in cell culture conditions. The coating agents poly-lysine (Sigma) and octadecyltrichlorosilane (OTS; Sigma) were also used to promote adherence of the proteins to the bottom of each well. For coating with poly-lysine, 250 µl of a 0.5% (w/v) solution of poly-lysine was incubated into each well. The plate was incubated for 5 min and the solution was removed. The plate was then incubated with the protein solution at 4°C overnight or left to dry overnight, followed by the same procedure described above. For coating with OTS, 250 µl of a solution of 0.5% (w/v) OTS was pipetted into each well and dried for 5 min. The protein solutions were then deposited into each well and the plate was processed as described above. Precipitation was monitored regularly by A_650_ turbidity readings, by visual inspection, and by using a cell culture inverted microscope (Diaphot; Nikon, Tokyo, Japan) at a magnification of 400X.

### Estimation of the Number of Apatite Crystals Bound to Each Fetuin-A or Albumin Molecule under Conditions that Produce Optimal Turbidity of Seeded Nanoparticles

The number of apatite crystals bound to each molecule of BSF or HSA was calculated for the experiment described in Fig. 6, under conditions that produced maximal precipitation of seeded nanoparticles following one month of incubation. For instance, when 0.7 mM of precipitating reagents was added to the three different protein solutions, followed by incubation for one month, maximal turbidity was observed at 21 µg/ml for BSF and at 0.4 mg/ml for HSA. An example of the calculation performed is described here for 0.7 mM of precipitating reagents added to DMEM in the presence of BSF at a concentration of 21 µg/ml. We estimated the number of apatite crystals bound to each protein molecule for these conditions by dividing the number of phosphate ions by the number of protein molecules present in the well. Since the molar concentration of phosphate ions present in the well was lower than that of calcium ions, we considered that phosphate would be the limiting factor for the formation of apatite crystals under these conditions. To calculate the number of phosphate ions present in the well, we added the concentration of phosphate ions used (0.7 mM) to the concentration of phosphate already present in DMEM (0.9 mM). This number was then converted to the number of moles of phosphate present in the well which contained 1 ml of solution (1.6×10^−6^ moles/well). This value was multiplied by Avogadro's number (6.023×10^23^ atoms/mole) in order to obtain the number of phosphate ions present in the well (9.64×10^17^). We assumed that all the phosphate ions present in the well would form apatite crystals after prolonged incubation. Thus, the number of phosphate ions present in the well (9.64×10^17^) was divided by the number of phosphate ions present in a single crystal unit of apatite (this latter value was averaged to 63 phosphate ions based on earlier estimations, see ref. 94). The estimated number of apatite crystals (1.53×10^16^) was then divided by the number of BSF molecules present in the well at a concentration of 21 µg/ml (2.64×10^14^ molecules of BSF) in order to obtain the number of apatite crystals bound to each molecule of BSF (58 apatite units/BSF molecule).

### Sodium Dodecyl Sulfate Polyacrylamide Gel Electrophoresis (SDS-PAGE)

NB-like particles containing fetuin-A and/or albumin like the ones shown in Fig. 8 were prepared by diluting BSF (Sigma) at a final concentration of 20–160 µg/ml in DMEM or HSA (Talecris) at a final concentration of 0.2–1.6 mg/ml in DMEM. The precipitating reagents CaCl_2_ and NaH_2_PO_4_ were then added each at a final concentration of 3 mM. A final volume of 1 ml of DMEM was used. Incubation was done in cell culture conditions for 1 month. The particles were then pelleted by centrifugation at 16,000× g for 15 min at room temperature. The pellet was washed twice with HEPES buffer using the same centrifugation steps. The particles were resuspended in 50 µl of 50 mM EDTA. Each sample was mixed with the 5X “loading buffer” (0.313 M Tris-HCl pH 6.8, 10% SDS, 0.05% bromophenol blue, 50% glycerol, 12.5% β-mercaptoethanol) to obtain a final concentration of “loading buffer” of 1X in a volume of 20 µl. 50 mM EDTA was used to dilute the samples. The protein solutions were heated at 95°C for 5 min and were subsequently loaded on a 10% SDS-polyacrylamide gel. For Fig. 8A, BSF-NLP were prepared by using DMEM (final volume of 1 ml) containing BSF (Sigma) at 20 µg/ml (lane 1), 40 µg/ml (lane 2), 80 µg/ml (lane 3), and 160 µg/ml (lane 4), followed by addition of CaCl_2_ and NaH_2_PO_4_ each to 3 mM and incubation in cell culture conditions for 1 month. Following incubation, the particles were pelleted as described above and washed twice with DMEM. The pellet was resuspended in 50 µl of 50 mM EDTA and 4 µl of each sample was loaded in the lanes described above. For Fig. 8B, HSA-NLP were prepared in a similar manner by using DMEM containing HSA (Sigma) at 0.2 mg/ml (lane 1), 0.4 mg/ml (lane 2), 0.8 mg/ml (lane 3), and 1.6 mg/ml (lane 4). For Fig. 8C, BSF-HSA-NLP were prepared similarly by using DMEM containing both proteins at the concentrations mentioned above. Gel electrophoresis was performed using a mini-gel system (Hoefer, Holliston, MA, USA). The gels were stained with Coomassie blue as described earlier [2].

### Protein Quantification

To quantify proteins, a standard curve was prepared by diluting a stock solution of BSA (Sigma) in double-distilled water at various concentrations varying from 10 to 50 mg/ml. Four volumes of each protein solution were mixed with one volume of dye reagent concentrate (Bio-Rad Laboratories) using a final volume of 1 ml of solution. The solutions were mixed and the optical density was monitored with a spectrophotometer (Molecular Devices) at a wavelength of 595 nm. A “blank” solution containing only the dye reagent diluted in double-distilled water as described above was used to subtract the optical density seen without proteins. A graph of optical density at 595 nm was plotted against the concentration of proteins from the standards. The samples with unknown concentration of proteins were processed the same way as the standard solutions. The protein concentration of the unknown was evaluated based on the optical density of the protein solutions and interpolation from the graph prepared. The solutions of unknown concentration were diluted until the optical density value obtained was within the linear part of the graph. The values of proteins mentioned represented average of experiments performed in triplicates.

### Preparation of Calcium Granules from Serum

Calcium granules were prepared as described earlier for serum pellets [3]. Briefly, the granules shown in this study (labeled as “Calcium Granules”) were prepared by adding sterile solutions of either 0.25 M CaCl_2_ and/or 0.25 M Na_2_HPO_4_ (both at pH 7.4) to FBS (2.5 ml) at final concentrations of either 48 mM CaCl_2_ (used for Figs. 9G, 10D, 12D, 13D, 14D, and 15G), 24 mM Na_2_HPO_4_ (Figs. 9H, 10E, 12E, 13E, 14E, and 15H), or 2 mM of both CaCl_2_ and Na_2_HPO_4_ (Figs. 9I, 10F, 12F, and 14F). Calcium granules were also prepared in HS (2.5 ml) by diluting 2 mM of both CaCl_2_ and Na_2_HPO_4_ (Figs. 13F and 15I). The ion solutions were filtered through both 0.2-µm and 0.1-µm membranes prior to use and were added in a drop-wise manner with vigorous shaking in order to avoid spontaneous precipitation. Treated sera were incubated at room temperature overnight, followed by centrifugation at 16,000× g for 1 hour, and washing steps using HEPES buffer and the same centrifugation steps. The granules were resuspended in 100 µl of HEPES buffer and were used for the various microscopy and spectroscopy analyses.

### Electron Microscopy

For scanning electron microscopy (SEM), washed particles, calcium granules, and NB specimens were resuspended in double-distilled water. A small aliquot of the sample was deposited on formvar carbon-coated grids (Electron Microscopy Sciences, Fort Washington, PA, USA). The excess liquid was removed with an absorbent paper and the grids were dried overnight under a laminar flow hood. Prior to observation, the specimens were coated with gold for 90 sec. SEM observations were conducted using a SEM S-5000 field-emission scanning electron microscope (Hitachi Science Systems, Tokyo, Japan).

For transmission electron microscopy (TEM), washed particles and NB samples were deposited on formvar carbon-coated grids and were dried overnight as described above. All TEM observations were performed without staining. Commercially available calcium carbonate (CaCO_3_, A.C.S. grade reagent, purity 99.6%, Mallinckrodt Baker, Inc., Phillipsburg, NJ, USA), calcium phosphate tribasic (Ca_3_(PO_4_)_2_, Kanto Chemical Co., Tokyo, Japan) and HAP (buffered aqueous suspension, 25% solid, Sigma) were diluted into DMEM or double-distilled water and processed like the other samples as controls.

For thin-sections, washed particles and NB samples were dehydrated with two washes of 100% ethanol. The samples were incubated with Epon 812 resin (Electron Microscopy Sciences) with gentle end-to-end agitation overnight at room temperature. The samples were then centrifuged at 16,000× g for 15 min and incubated at 72°C for 2 days to allow resin polymerization. Thin-sections were prepared using a Leica Ultracut UCT microtome (Leica Microsystems GmbH, Wetzlar, Germany). Thin-sections were deposited on formvar carbon-coated grids. TEM observations and electron diffraction patterns were performed with a JEOL JEM-1230 transmission electron microscope (JEOL, Tokyo, Japan) operated at 120 keV.

### Energy-Dispersive X-Ray Spectroscopy

Washed particles, calcium granules, and NB specimens were resuspended in double-distilled water and deposited on formvar carbon-coated grids. The grids were dried overnight in a laminar flow hood. The samples were observed with a SEM S-3000N scanning electron microscope (Hitachi Science Systems) and the energy-dispersive X-ray spectroscopy (EDX) analysis was performed using an EMAX Energy EX-400 EDX device (Horiba, Tokyo, Japan). Each sample was irradiated for 30 sec and data acquisition was performed with the EMAX software (Horiba) in point mode analysis. Three different areas of each sample were analyzed to ensure homogenous readings.

### Nanoparticle Sizing by Dynamic Laser Scattering

Sizing of mineral nanoparticles was performed using DLS according to established protocols [222], [223]. Briefly, the particles were prepared by adding calcium and phopshate ions into DMEM (final volume of 1 ml) each at a concentration of 1 mM. Alternatively, particles were also prepared by adding 1 mM of calcium and phopshate ions each into DMEM containing 2 mg/ml of either BSF (Sigma) or HSA (Sigma). The samples were prepared directly in disposable plastic cuvettes and were shaken vigorously prior to reading. Some samples were incubated for various periods of time varying from 5 min to several days before measurement. Measurements consisted of an average of 10 individual measurements taken at an interval of 10 sec. Each measurement was performed in triplicates using the Malvern Zetasizer Nano-Series (Malvern Instruments Ltd., Malvern, Worcestershire, UK). A laser consisting of a helium-neon lamp with a wavelength of 633 nm was used and the measurements were performed at an angle of 90° from the sample.

### Fourier-Transformed Infrared Spectroscopy

Washed and dried samples were mixed with potassium bromide (Sigma) to obtain a ratio of 1∶100 (w/w). The samples were compressed with a hand press to form a thin transparent pellicle. FTIR spectra were acquired with a Nicolet 5700 FTIR spectrometer (Thermo Fisher Scientific, Waltham, MA, USA) equipped with a deuterated triglycine sulfate (DTGS) detector. The spectra were obtained at a resolution of 4 cm^−1^ and at wavelengths ranging between 4,000 cm^−1^ to 400 cm^−1^. Each spectrum represented an average of 32 consecutive scans. For comparison, the commercially available controls of CaCO_3_, Ca_3_(PO_4_)_2_, and HAP described earlier were diluted and washed in either double-distilled water (shown in Fig. 13J, K, L, respectively) or DMEM (data not shown), followed by processing for FTIR analysis. The use of DMEM produced dampening of signals and significant depressions/troughs that could not be explained at this time.

### Micro-Raman Spectroscopy

Aliquots of washed particles and NB samples were processed for micro-Raman spectroscopy as described before [2]. Briefly, the samples were resuspended in double-distilled water, deposited on glass slides, and dried overnight. Micro-Raman spectra were obtained using the inVia Raman confocal microscope (Renishaw, Stonehouse, UK) equipped with a charge-coupled device (CCD) detector. A laser beam of 633 nm operated at 17 mW was used as the excitation source. Controls of CaCO_3_, Ca_3_(PO_4_)_2_, and HAP were diluted and washed in either double-distilled water (shown in Fig. 14J, K, L, respectively) or DMEM (data not shown), followed by processing for micro-Raman analysis. In this case, the use of DMEM produced increased carbonate signals for the Ca_3_(PO_4_)_2_ sample which may have originated from prolonged contact with CO_2_ from air.

### Powder X-Ray Diffraction Spectroscopy

X-ray diffraction spectroscopy was performed as described earlier [2]. Briefly, washed particles and NB specimens were deposited on glass slides and dried overnight. X-ray diffraction was performed using a D5005 X-ray diffractometer (Bruker AXS, Madison, WI, USA) with a source of X-ray consisting of a copper tube operating at 40 kV. X-ray diffraction spectra were searched against the database of the Joint Committee on Powder Diffraction and Standards (JPCDS) in order to identify the chemical formula of the crystalline compound under study.
